# Variant-specific deleterious mutations in the SARS-CoV-2 genome reveal immune responses and potentials for prophylactic vaccine development

**DOI:** 10.3389/fphar.2023.1090717

**Published:** 2023-02-07

**Authors:** Md. Aminul Islam, Shatila Shahi, Abdullah Al Marzan, Mohammad Ruhul Amin, Mohammad Nayeem Hasan, M. Nazmul Hoque, Ajit Ghosh, Abanti Barua, Abbas Khan, Kuldeep Dhama, Chiranjib Chakraborty, Prosun Bhattacharya, Dong-Qing Wei

**Affiliations:** ^1^ Advanced Molecular Lab, Department of Microbiology, President Abdul Hamid Medical College, Karimganj, Bangladesh; ^2^ COVID-19 Diagnostic lab, Department of Microbiology, Noakhali Science and Technology University, Noakhali, Bangladesh; ^3^ Department of Biochemistry and Molecular Biology, Shahjalal University of Science and Technology, Sylhet, Bangladesh; ^4^ Department of Statistics, Shahjalal University of Science and Technology, Sylhet, Bangladesh; ^5^ Department of Gynecology, Obstetrics and Reproductive Health, Faculty of Veterinary Medicine and Animal Science, Bangabandhu Sheikh Mujibur Rahman Agricultural University, Gazipur, Bangladesh; ^6^ Department of Bioinformatics and Biological Statistics, School of Life Sciences and Biotechnology, Shanghai Jiao Tong University, Shanghai, China; ^7^ Zhongjing Research and Industrialization Institute of Chinese Medicine, Zhongguancun Scientific Park, Nayang, Henan, China; ^8^ Division of Pathology, ICAR-Indian Veterinary Research Institute, Bareilly, Uttar Pradesh, India; ^9^ Department of Biotechnology, School of Life Science and Biotechnology, Adamas University, Kolkata, West Bengal, India; ^10^ COVID-19 Research @KTH, Department of Sustainable Development, Environmental Science and Engineering, KTH Royal Institute of Technology, Stockholm, Sweden; ^11^ Peng Cheng Laboratory, Shenzhen, Guangdong, China

**Keywords:** COVID-19, SARS-CoV-2, deleterious mutation, unique mutation, delta variant, omicron variant, immune response, vaccine designing

## Abstract

**Introduction:** Coronavirus disease 2019 (COVID-19), caused by SARS-CoV-2, has had a disastrous effect worldwide during the previous three years due to widespread infections with SARS-CoV-2 and its emerging variations. More than 674 million confirmed cases and over 6.7 million deaths have been attributed to successive waves of SARS-CoV-2 infections as of 29th January 2023. Similar to other RNA viruses, SARS-CoV-2 is more susceptible to genetic evolution and spontaneous mutations over time, resulting in the continual emergence of variants with distinct characteristics. Spontaneous mutations of SARS-CoV-2 variants increase its transmissibility, virulence, and disease severity and diminish the efficacy of therapeutics and vaccines, resulting in vaccine-breakthrough infections and re-infection, leading to high mortality and morbidity rates.

**Materials and methods:** In this study, we evaluated 10,531 whole genome sequences of all reported variants globally through a computational approach to assess the spread and emergence of the mutations in the SARS-CoV-2 genome. The available data sources of NextCladeCLI 2.3.0 (https://clades.nextstrain.org/) and NextStrain (https://nextstrain.org/) were searched for tracking SARS-CoV-2 mutations, analysed using the PROVEAN, Polyphen-2, and Predict SNP mutational analysis tools and validated by Machine Learning models.

**Result:** Compared to the Wuhan-Hu-1 reference strain NC 045512.2, genome-wide annotations showed 16,954 mutations in the SARS-CoV-2 genome. We determined that the Omicron variant had 6,307 mutations (retrieved sequence:1947), including 67.8% unique mutations, more than any other variant evaluated in this study. The spike protein of the Omicron variant harboured 876 mutations, including 443 deleterious mutations. Among these deleterious mutations, 187 were common and 256 were unique non-synonymous mutations. In contrast, after analysing 1,884 sequences of the Delta variant, we discovered 4,468 mutations, of which 66% were unique, and not previously reported in other variants. Mutations affecting spike proteins are mostly found in RBD regions for Omicron, whereas most of the Delta variant mutations drawn to focus on amino acid regions ranging from 911 to 924 in the context of epitope prediction (B cell & T cell) and mutational stability impact analysis protruding that Omicron is more transmissible.

**Discussion:** The pathogenesis of the Omicron variant could be prevented if the deleterious and persistent unique immunosuppressive mutations can be targeted for vaccination or small-molecule inhibitor designing. Thus, our findings will help researchers monitor and track the continuously evolving nature of SARS-CoV-2 strains, the associated genetic variants, and their implications for developing effective control and prophylaxis strategies.

## 1 Introduction

The ongoing COVID-19 caused by SARS-CoV-2 has wreaked havoc on global economies, businesses, communities, and public health due to widespread infections in humans (Hoque et al., 2020; Islam et al., 2021; Jakariya et al., 2022; Rakib et al., 2022). Causingflu-like symptoms and nearly 2%–5% mortality, the COVID-19 pandemic worsened due to continuously emerging SARS-CoV-2 variants from time to time, contributing to the surge in infections and deaths in multiple waves (Dhama et al., 2022; Jacobs et al., 2023; Islam et al., 2022a; Islam et al., 2022b).

Similar to other RNA viruses, SARS-CoV-2 is prone to mutations that produce new variants, creating difficulties in developing effective antiviral drugs and vaccines against this virus (Ahmed et al., 2020; Jacobs et al., 2023; Sakib et al., 2021; Wang et al., 2020). Over 2 years, the SARS-CoV-2 virus has evolved through multiple new mutations and various genetic variants, such as Alpha (B.1.1.7) with seven, Beta (B.1.351) with nine, Gamma (P.1) with 12, Delta (B.1.6, B.1.6.2) with 17, Omicron (B.1.1.529) and Neocov variant with 32 new mutations in the spike protein gene (Ahmed et al., 2021; Chakraborty et al., 2022a; Chakraborty et al., 2022b; Chakraborty et al., 2022c; Chakraborty et al., 2022d; Chakraborty et al., 2022e). These variants have spread to various regions of the world, and among them, variants of concern (VOCs) such as Delta, Omicron, and their sub-lineages have caused significant risk to public health (Hossain et al., 2021; Islam et al., 2021, Islam et al., 2022a). However, the number of fatalities caused by various SARS-CoV-2 variants fluctuates considerably (Islam et al., 2022b; Islam et al., 2022c; Islam et al., 2022d). So far, only a few comprehensive studies have been conducted, incorporating all SARS-CoV-2 variants (Davies et al., 2021; Imai et al., 2021; Walensky et al., 2021). Each variant of SARS-CoV-2 evolves with greater pathogenicity, infecting and evading the immune system of the host, leading to vaccine breakthrough infections, re-infections *via* overpowering vaccine efficacy and antibodies-based therapies (Roy et al., 2022a; Chakraborty et al., 2022b; Roy et al., 2022b). Eight of the 23 mutations from the original Wuhan-Hu-1 strain (Accession NC_045512, version NC_045512.2) that make up B.1.1.7 are in the spike protein, in which N501Y, spike deletion 69–70 del, and P681H are the three mutations that are considered to have the most biological impact (Yang et al., 2021). In addition to D614G, B.1.351 contains a cluster of mutations (242–244 del and R246I) in the N-terminal domain, three mutations (K417N, E484K, and N501Y) in the receptor-binding domain (RBD), and one mutation (A701V) near the furin cleavage site. Among the three significant mutations that the spike protein RBD carries, E484K is situated in a loop region away from direct hACE2 (human angiotensin-converting enzyme 2) contact, while mutations in P.1: N501Y and K417T interact with human ACE2 (hACE2). (Faria et al., 2021; Hossain et al., 2021; Chandran et al., 2022). Compared with the first strain (alpha strain) of SARS-CoV-2, the Delta variant B.1.617.2 has 23 mutations. Twelve such mutations have been found in the spike protein, including T19R, L452R, T478K, D614G, P681R, and D960N (Shiehzadegan et al., 2021; Asghar et al., 2022).

To prevent SARS-COV-2 infections, a few COVID-19 vaccines utilizing distinct vaccine platforms have been developed (Hossain et al., 2021). In addition, vaccination campaigns and booster shots are underway in majority of nations to provide the populace with protective immunity. Some drugs, including recent oral antiviral drugs nirmatrelvir/ritonavir and molnupiravir, and therapies have been found effective and used for emergency purposes. However, an effective vaccine to tackle the menace of emerging variants of SARS-CoV-2, particularly such as Delta and Omicron, is still awaited (Aleem et al., 2022; Barouch, 2022). Designing effective vaccines against SARS-CoV-2 variants is a very challenging issue as it is required to develop mutation-proof, variant-specific, and universal vaccines to prevent the spread of COVID-19 (Gong et al., 2022; Park et al., 2022). In this regard, investigating SARS-COV-2 variants and unique mutations is essential for developing effective anti-COVID-19 drugs and vaccines (Rakib et al., 2021; Jakariya et al., 2021).

Therefore, in the present study, we analyzed the mutation patterns of 10,531 SARS-CoV-2 genomes of 12 variants from around the world in terms of frequency, type, the ratio of synonymous to non-synonymous mutations, and zone analysis. Based on the most significant mutations, we focused primarily on the Delta and Omicron variants. The impact of deleterious mutations on B- and T cell responses has also been investigated to identify the specific mutations responsible for the downregulation of the host immune system. The structural conformation of the most concerning mutations, such as mutations of spike proteins, was also determined. The results of this study will aid the scientific community in the development of variant-specific, more accurate, effective, and successful vaccines, as well as provide possible indications for the regions of deleterious variant viral proteins in the Omicron variant. Therefore, in the present study, we analyzed the mutation patterns of 10,531 SARS-CoV-2 genomes of 12 variants from around the world in terms of frequency, type, the ratio of synonymous to non-synonymous mutations, and zone analysis. Based on the most significant mutations, we focused primarily on the Delta and Omicron variants. The impact of deleterious mutations on B- and T cell responses has also been investigated to identify the specific mutations responsible for the downregulation of the host immune system. The structural conformation of the most concerning mutations, such as mutations of spike proteins, was also determined. The results of this study will aid the scientific community in the development of variant-specific, more accurate, effective, and successful vaccines, as well as provide possible indications for the regions of deleterious variant viral proteins in the Omicron variant.

## 2 Materials and methods

### 2.1 Genomic data collection and filtering

The first SARS-CoV-2 whole genome sequence (WGS) was deposited on 5 January 2020, in GenBank (accession number: NC_045512.2). Currently, the total number of submitted sequence numbers is 127,108,83 (accordingly, Global Initiative on Sharing All Influenza Data (GISAID), 20 August 2022) (https://www.gisaid.org/) (Khare et al., 2021).

**FIGURE 1:**
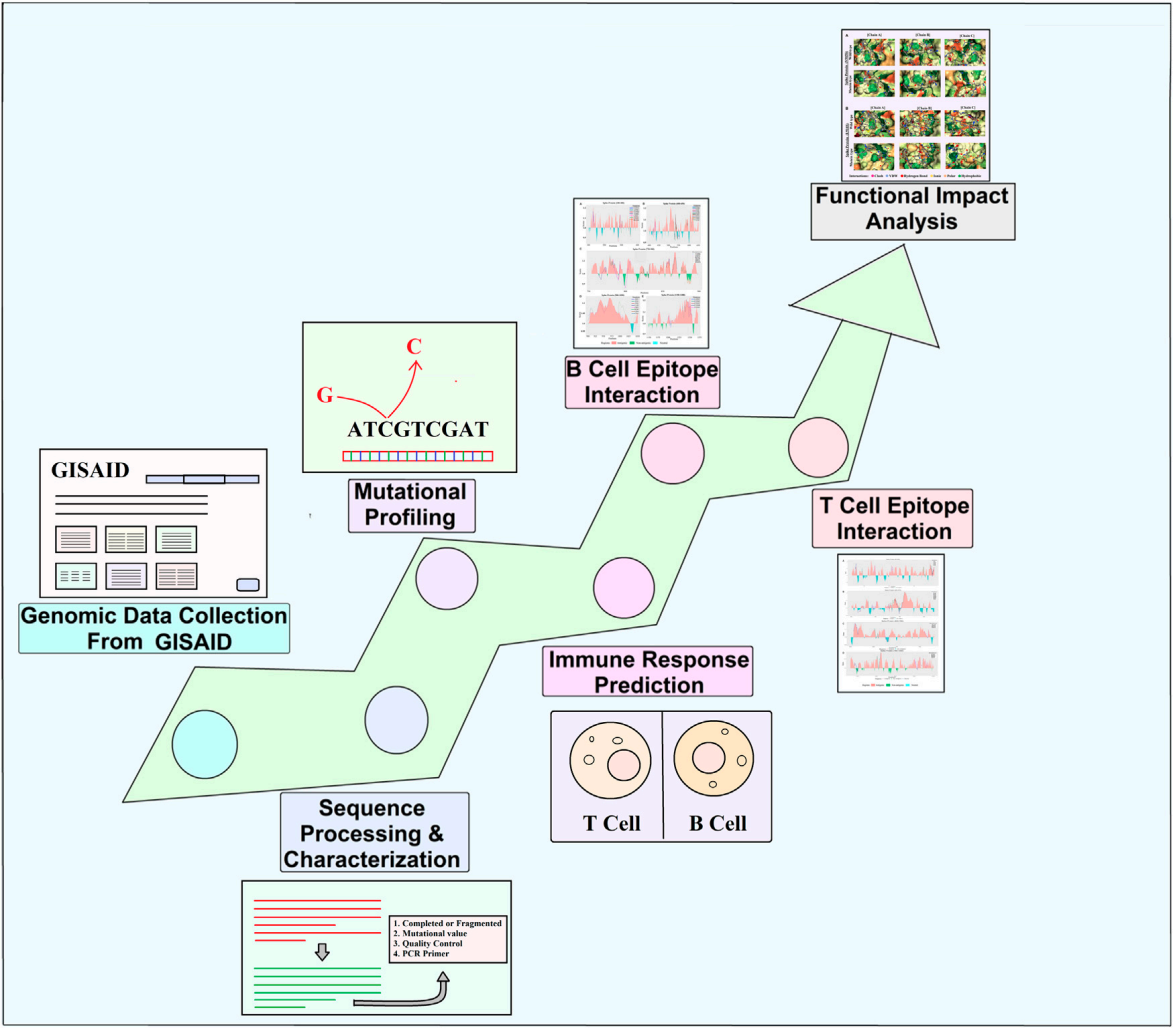
Schematic diagram representing several stages starting from genomic data collection to analysis.

**FIGURE 2:**
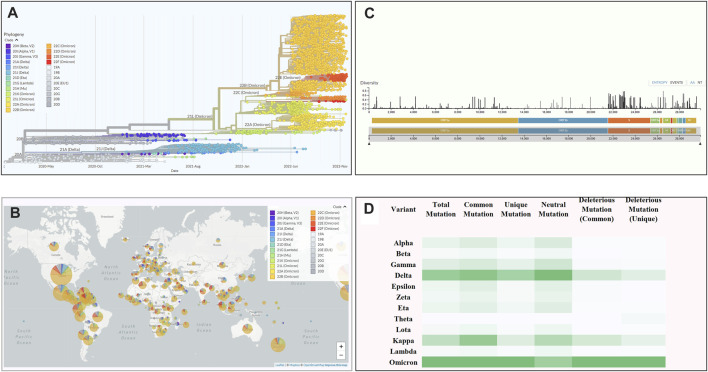
An overview of the scenario of coronavirus. **(A)** Phylogenetic tree of SARS-CoV-2 depicting variant emergence from January 2020 to January 2022. **(B)** A world map depicting the frequency of variants occurring across the world. **(C)** The total number of mutations occurring across the whole genome of SARSCoV-2. The length of the bar determines the diversity of mutations at a specific position on the genome. (D) The total number of mutations along specific types like common, unique, neutral, and deleterious across all the variants of SARS-CoV-2. (Analysed by Nextstrain: https://nextstrain.org).

In this study, we filtered 214,459 sequences from the whole extracted GISAID dataset based on specific criteria (all of the complete genome sequences retrieved including both death and alive p with high coverage read, patient status, and collection date from the human host). It can be noted that the collected sequences retrieved from dead patients do not influence the whole analyzed dataset. Only complete genomes with a size greater than 29,000 bp were selected, and those with low coverage, possessing >5% of N, were filtered out. Finally, the filtered process ultimately resulted in 10,531 complete genomes of SARS-CoV-2 for this study, which ranged from January 2020 to January 2022 (Figure 2) (Supplementary Table S9). Pyfasta (https://github.com/brentp/pyfasta) was used to split the total genome into six separate files. The entire procedure used in this study is presented in Figure 1.

### 2.2 Mutation analysis

Sequence analysis, alignment, and clustering were performed using NextClade (https://clades.nextstrain.org/), an advanced tool for SARS-CoV-2 sequence analysis (Aksamentov et al., 2021). Mutational frequency count was executed using Python script (uploaded to GitHub). Error-free sequence data were normalized (filtered to reduce data redundancy and eliminate undesirable characteristics) using Python script and advanced Excel options. The most frequent mutations and their deleterious impact were further analyzed using PredictSNP (https://loschmidt.chemi.muni.cz/predictsnp/) (Bendl et al., 2014), PolyPhen 2 (http://genetics.bwh.harvard.edu/pph2/) (Adzhubei et al., 2013), SIFT (https://sift.bii.a-star.edu.sg/) (Ng, 2003), PROVEAN (http://provean.jcvi.org/index.php) (Choi & Chan, 2015), and the I-Mutant Suite (http://gpcr2.biocomp.unibo.it/cgi/predictors/I-Mutant3.0/I-Mutant3.0.cgi) (Capriotti et al., 2005). Deleterious or non-synonymous mutations are critically analyzed and cross-checked using these tools. To define deleterious mutation, a threshold value of -2.5 was determined to ensure highly balanced accuracy (Choi & Chan, 2015). Therefore, mutations having a value smaller than -2.5 were identified as deleterious.

### 2.3 Prediction of immune responses

#### 2.3.1 B cell epitope prediction

The antibody epitope prediction tool (http://tools.iedb.org/bcell/) from IEDB (Immune Epitope Database) was used to detect the B cell antigenic regions. In this tool, a semi-empirical method named “Kolaskar & Tongaonkar antigenicity” was used (Kolaskar & Tongaonkar, 1990) as the analyzed method for B cell antigenic prediction as the method ensures 75% accuracy. The antigenic region refers to the epitope that is most likely to bind to B cells whereas the non-antigenic region shows lower or no affinity to bind to B cells. Each mutation sequence was derived using Python script, which is available from GitHub on request (Islam et al., 2022e; Islam et al., 2023). Based on the antigen propensity score collected from the tool, linear B cell epitope area graphs of wild sequences were constructed using ggplot2 (version 3.3.5), ggh4x (version 0.1.2.1.9), and cowplot (version 1.1.1) in the R statistical environment (version 3.6.1). Graphs are presented as antigenic (>threshold value) and non-antigenic (<threshold value) regions based on a threshold value of 1 as the average score for most of the sequence is around 1. Mutations having increased antigenic scores are likely to bind B cells, therefore, enabling the host immunity to act to sort the fight. But the opposite case refers to the mutations that are becoming immune to the host B cell. Codes generated for the graphs can be accessed from GitHub upon request. Mutations are marked as line graphs with a point mark on the mutation position. The significant mutation was marked by comparing the antigenic propensity score of mutated proteins with that of wild-type proteins. If the score of the mutated proteins dropped in the antigenic region, it was marked as significant.

#### 2.3.2 T Cell epitope prediction

Wild type sequenced for each protein was then used to perform T Cell epitope prediction with the “CD4 T Cell immunogenicity prediction tool” of IEDB (http://tools.iedb.org/CD4episcore/) (Paul et al., 2015; Dhanda et al., 2018). The threshold value of the combined score was set to 50%. The results give immunogenicity scores of several predicted immunogenic segments for a particular peptide. However, for more accuracy combined scores were taken into concern as the score not only includes immunogenicity scores but also scores from the seven allele method (an optimized method for prediction of HLA responses) (Dhanda et al., 2018). The mutations occurring in these predicted immunogenic segments were then used to perform with the same tool to observe the change in combined score due to that particular mutation. According to the result, a higher value indicates a lower tendency to provoke an immune response. Therefore, the mutations which caused an increase in the combined score beyond 50%, were marked as the most significant.

### 2.4 Effects of missense mutation on protein stability and protein-protein binding affinity

Mutations likely to suppress the immune response for both B- and T cells are selected for the next level of analysis to demonstrate their functional impacts. Most of the data, except spike protein, were collected with the COVID-3D database (http://biosig.unimelb.edu.au/covid3d/). For the spike protein of the delta variant (PDB ID:7jji), the “DynaMut” database (http://biosig.unimelb.edu.au/dynamut/) (C. H. Rodrigues et al., 2018) was used to find ΔΔG to identify the stabilizing effect of the mutations."Δ Vibrational Entropy Energy”, was also determined to identify the impact on structural flexibility, and deformation and fluctuation analysis. “mCSM-PPI2" (http://biosig.unimelb.edu.au/mcsm_ppi2/) (C. H. M. Rodrigues et al., 2019) was used to predict the affinity changes and differences in the interaction between the wild-type and the mutant for both delta and omicron. The effects of missense mutation on protein stability for omicron were analyzed with “mCSM Stability” (https://biosig.lab.uq.edu.au/mcsm/) (Pires et al., 2014). To perform protein and/or gene functional analysis, the 3D structure of spike protein was collected from the Protein Data Bank (https://www.rcsb.org/).

### 2.5 Normalization, data validation, and machine learning

In order to overcome the biases in the various factors or platforms in mutation-based studies normalization is a crucial pre-processing step. A sample-wise normalization is a typical approach in intra-study analysis. Numerous established sample-wise normalization techniques have been created and used, such as simple standardization (standardize to zero mean and unit variance). This data was subjected to standard normalization or standardization prior to validation under machine learning, which confirmed independent and unbiased findings of the mutational structure and at the same time standardized each sample to a mean of zero and a unit variance.

The overall analysis was validated using machine learning and Bio-python. We used the following set of different classifiers, which includes probabilistic ones: Logistic Regression, Linear Discriminant Analysis, Support Vector Machines (SVM) (Cortes & Vapnik, 1995), and Neural Networks (Jantzen, 1998). We used a specific performance measure under the ROC curve, which is the proportion of correctly classified samples. The classes were balanced so that the accuracy metrics worked correctly.

We trained and turned the classifiers on the SARS-CoV-2 mutational databases (Supplementary Table S7). To avoid overfitting, five-fold cross-validation was performed. Specifically, a dataset was split into five approximately equal parts (or folds), of which four parts were used for training and the fifth part for validation. This procedure was repeated five times with different parts used for validation each time. The performance measure is an average of the values computed at each iteration. While the dataset is usually split into folds randomly, we created folds such that all mutations in the same protein fell into the same fold. This was done to avoid over fitting in the situation where we train and test a classifier on the same protein.

## 3 Results

### 3.1 Mutational analysis of SARS-CoV-2 variants

Through a comprehensive mutational analysis of 10,531 complete genomes (The number of sequences had been retrieved in a sequential way mentioned in the Genomic data collection and filtering portion of the methodology Section 2.1 and neither these sequences nor the partial sequences under different variants were compromised by any factor or biased) of SARS-CoV-2, we detected 16,954 common and unique mutations (which were continuously filtered according to the mutation score, and only a specific amount of highly deleterious mutations were focused for further mutational analysis) compared to the Wuhan-Hu-1 reference strain (Accession NC_045512, VersionNC_045512.2). Based on the mutational spectra, we found 12 variants of SARS-CoV-2, including 1,076 genomes from Alpha, 686 from Beta, 1,350 from Gamma, 1884 from Delta, 805 from Epsilon, 461 from Zeta, 1,468 from Eta, 133 from Theta, 305 from Lota, 207 from Kappa, 209 from Lambda, and 1947 from Omicron variants. The Omicron variant showed a comparatively higher mutation rate (Figure 2D).

Among these variants, the Delta variant (B.1.617.2) showed 4,468 mutations, of which 1,204 were deleterious having a value less than the threshold (−2.5). Moreover, among the detected mutations in the Delta variant, 66% were unique, and not reported in other variants. By contrast, the highest number of mutations (*n* = 6,307) were found in the Omicron variant in which 4,178 (67.8%) were predicted to be unique while 1,092 (26.14%) were identified as deleterious (Table 1).

**TABLE 1:** Comparative view of different types of mutations in 12 variants of SARS-CoV-2.

Variant	Pango lineage	Total retrieved sequences	Total mutations	Common mutations	Unique mutations	Deleterious mutations (common)	Deleterious mutations (unique)
Alpha	B.1.1.7	1076	1102	648	454	145	96
Beta	B.1.351	686	451	279	172	3	42
Gamma	P.1	1350	1427	812	615	173	133
Delta	B.1.617.2	1884	4468	1513	2952	361	844
Epsilon	B.1.427 B.1.429	805	1002	604	398	139	92
Zeta	P.2	461	726	461	265	108	62
Eta	B.1.525	1468	811	504	307	124	64
Theta	P.3	133	161	109	52	23	14
Lota	B.1.526	305	438	291	147	59	30
Kappa	B.1.617.1	207	1218	871	347	209	114
Lambda	C.37	209	367	224	143	58	40
Omicron	B.1.1.529	1947	6307	2129	4178	563	1092

**FIGURE 3:**
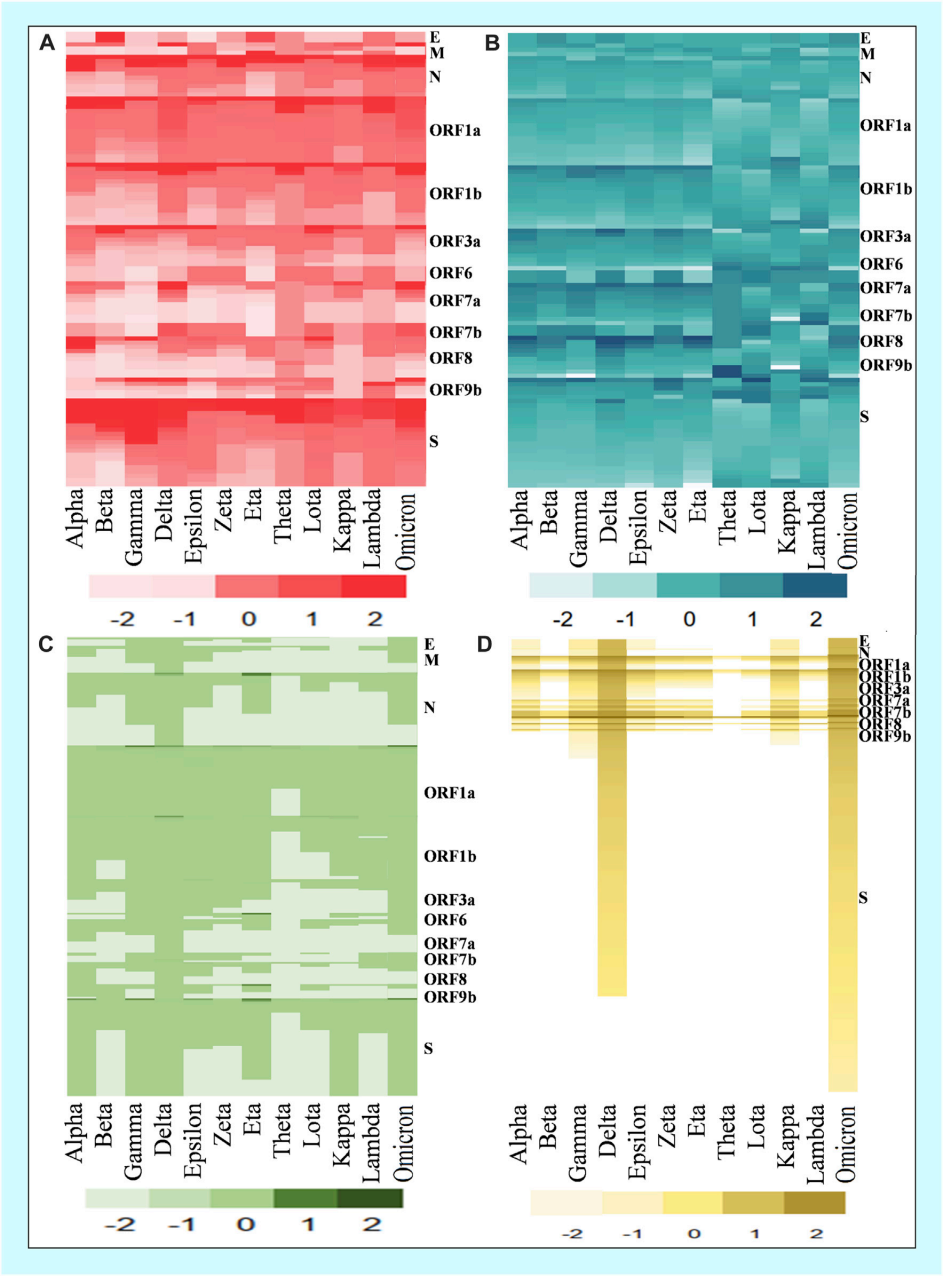
Comparison of common and unique deleterious mutation patterns in different variants. **(A)** Frequency of top common deleterious mutations of different proteins in twelve variants. **(B)** Deleterious mutation score of common mutations. **(C)** Frequency of top unique deleterious mutations of different proteins in twelve variants. **(D)** Deleterious mutation score of unique mutations.

In the mutational analysis, the highest number of mutations were detected in the S protein while the E protein contained the lowest (Figure 3). The mutation rate of the Omicron variant’s S protein was significantly higher than that of other variants. Amidst 876 mutations detected in the spike protein of the Omicron variant, 364 were common, 512 were unique, 412 were neutral, and 187 were deleterious. Further, the deleterious mutations included 187 common deleterious and 256 unique non-synonymous mutations. The Delta variant, on the other hand, had the second-highest number of mutational patterns, with 53 being unique deleterious mutations, whereas Kappa was found as the third-highest variant possessing 44 unique deleterious mutations. Conversely, the other variants such as Alpha, Gamma, Eta, Theta, Lota, Zeta, etc. had more neutral mutations in the S protein region than Omicron, Delta, and Kappa variants (Table 2).

**TABLE 2:** Comparison of mutations in the spike protein among 12variants of SARS-CoV-2.

Variant	Total mutation	Common mutation	Unique mutation	Neutral mutation	Deleterious mutation (common)	Deleterious mutation (unique)
Alpha	162	100	62	155	4	3
Beta	69	48	21	62	3	4
Gamma	215	119	96	200	8	7
Delta	678	326	352	560	65	53
Epsilon	125	92	33	118	5	2
Zeta	101	70	31	95	5	1
Eta	136	84	52	129	4	3
Theta	31	21	10	14	2	15
Lota	75	54	21	70	2	3
Kappa	421	295	126	316	61	44
Lambda	71	48	23	66	2	3
Omicron	876	364	512	412	187	256

### 3.2 Comparative analysis of the deleterious mutations

A deleterious mutation can be referred as a mutation for which the protein compound of a gene is not produced, or does not function, or interferes with normal function. Moreover, impairment of regulatory functions can be a possible outcome of deleterious regulatory agents. Accordingly, the functional impact of mutations was analyzed using PROVEAN (Protein Variation Effect Analyzer), which predicts the effect of amino acid (aa) substitution on the overall function of a protein. The lower scores for mutant (<−2.5) in PROVEAN, were marked as the most deleterious. In this study, the lowest score of Omicron was –11.248, found in the C3408S, while Delta showed the lowest score –12.869, was found in W55C. Interestingly, these three deleterious mutations were found in the ORF8 region of the SARS-CoV-2 genome. We further compared the mutation patterns between Omicron and Delta variants, which revealed that unique-deleterious mutations of these variants were significantly stable in the protein configuration (Table 3). By comparing all mutations of the SARS-CoV-2 variants, we found that mutations in the Omicron variants were more deleterious than the other 12 variants. The non-synonymous score of the Omicron variant was higher for most structural proteins and/or genes.

**TABLE 3:** Comparison of the deleterious mutations between Omicron and Delta variants of SARS-CoV-2 in their different genes.

Gene	Omicron (O, o)		Delta (Δ, δ)		Gene	Omicron (O, o)		Delta (Δ, δ)	
	Position	Score	Position	Score		Position	Score	Position	Score
E	R61L	−1.733	R61L	-2.8	ORF1b	C1367T	-10.71	H962I	-10.31
N	Q306T	−3.878	W55C	-12.869		Y470S	-9.75	C928W	-10.31
	G164S	−2.918	G99S	-3.861		Y1944T	-9.397	C942F	-10.31
	N150T	−2.645	P162L	-3.655		C143T	-8.549	C939F	-10.26
ORF1a	C3408S	−11.248	C2989K	-11.183		G343C	-8.378	G670L	-9.667
	W3004Y	−11.153	W4096L	-11.111		R1600N	-8.654	G674I	-9.667
	G2815L	−11.044	C4370F	-10.248		D1815T	-8.43	G550L	-9.667
	P4211S	−10.398	W3481L	-10.121		V345E	-8.289	P618I	-9.667
	Y3364C	−10.051	C2445S	-9.618		L428V	-8	Y916G	-9.663
	P2870S	−9.499	C2851S	-9.319		M557H	-8.8	Y822G	-9.663
	Y2301C	−9.404	G3795L	-9.316		G1022T	-8.627	D675V	-8.7
	L2298S	−9.305	G3809L	-9.316		T267N	-7.533	N694F	-8.7
	P3015L	-9.21	G3372I	-9.316		M915S	-7.398	Y537A	-8.7
	Y4280H	-9.11	F2834P	-9.293		V344E	-6.965	Y665A	-8.7
	G3546T	−9.05	Y4013P	-9.216		P1645S	-6.729	D456I	-8.4
	S2493Y	−8.185	Y4013G	-9.183		T453A	-6.533	G328C	-8.3
	D2627C	−8.143	P3642I	-9.042		R712C	-6.366	Y737T	-8.229
	S3264D	−7.948	C2913H	-8.534		C2551S	-6.206	Y819A	-8.167
	S3195N	−7.901	G2987L	-8.531		L1088T	-5.751	G2436C	-8.138
	N1576D	−7.817	C3867I	-8.416		V1012N	-5.751	N619I	-8.043
	D1600C	−7.804	C2984A	-8.387		N2694T	-5.708	G662L	-8.033
	G2258Y	−7.746	Y3811A	-8.385		H1897S	-5.583	Y1759C	-7.875
	F3554T	−7.657	G4244F	-8.385		E720Y	-5.09	P821A	-7.731
ORF3a	W193T	−7.276	G187C	-7.248		E1623A	-5.026	Q923F	-7.731
	G188L	−6.6	G172S	-4.457		C688V	-5.003	P528S	-7.73
	T32E	-5.505	T221K	-4.276		S583T	-4.971	Y971L	-7.502
	T229T	-4.276	N257Y	-4.257		T984E	-4.958	G940T	-7.502
	S162K	-3.886	K235T	-3.276		V2501Y	-2.89	D295Y	-7.419
	K61L	-3.6	S58I	-3.276		G2510T	-2.877	D842A	-7.396
	I62T	-3.39	A103P	-3.229		L342R	-2.767	P970C	-7.278
	V256D	-2.886	I47T	-3.067	ORF9b	P39T	-8.478	L52P	-7
ORF7a	E41T	-6	Q57R	-2.629		R47E	-5	I45T	-5
	Y97E	-5.825	Q38K	-2.629		L64T	-4	V76F	-4.87
	K2Y	-5.684	M19T	-5.571		A29S	-3.304	L14F	-4
	H47I	-4.667	H3Y	-3.071		V15T	-3	N35S	-3.43
	R78Y	-4.333	C58F	-7.333	S	D1199Y	-10.528	C301R	-9.89
	T57H	-4.333	C67F	-7.333		N824S	-10.076	N919W	-9.35
	V82Y	-4.667	G42D	−7		F318R	-9.813	C301L	-7.9
ORF7b	F19S	−7	W29C	−13		G496S	-9.424	T478K	-7.062
ORF8	C90T	−10.389	I10N	−5.389		Q498R	-9.0665	L452R	-6.066
	C25V	−10.389	Q23H	−4.722		Y505H	-8.709	R158G	-5.071
	G96C	−6.611	F120A	−4.056		N501Y	−8.3515	T19R	-4.075
	T80Y	−4.667	P70L	−4.056		Q594H	−7.994	P681R	-3.08
	H40T	−3.556	R115C	−3.722		N764K	−7.6365	D950N	-2.084
	H40S	−3.167	G8V	−3		N856K	−7.279	E484A	-1.09
	G77T	−2.944	D119V	−2.917		L981F	−6.9215	E484K	-0.09

Further, The Omicron variant showed a higher degree of deleterious mutations in ORF1a, ORF1b, ORF7a, ORF 8, and ORF 9b (Table 2). According to PROVEAN, –2.5 was the threshold value for deleterious mutation. Therefore, mutations with a score less than –2.5 were considered deleterious. Consequently, our findings indicate a negative correlation between the severity of a deleterious mutation with its score.

**FIGURE 4:**
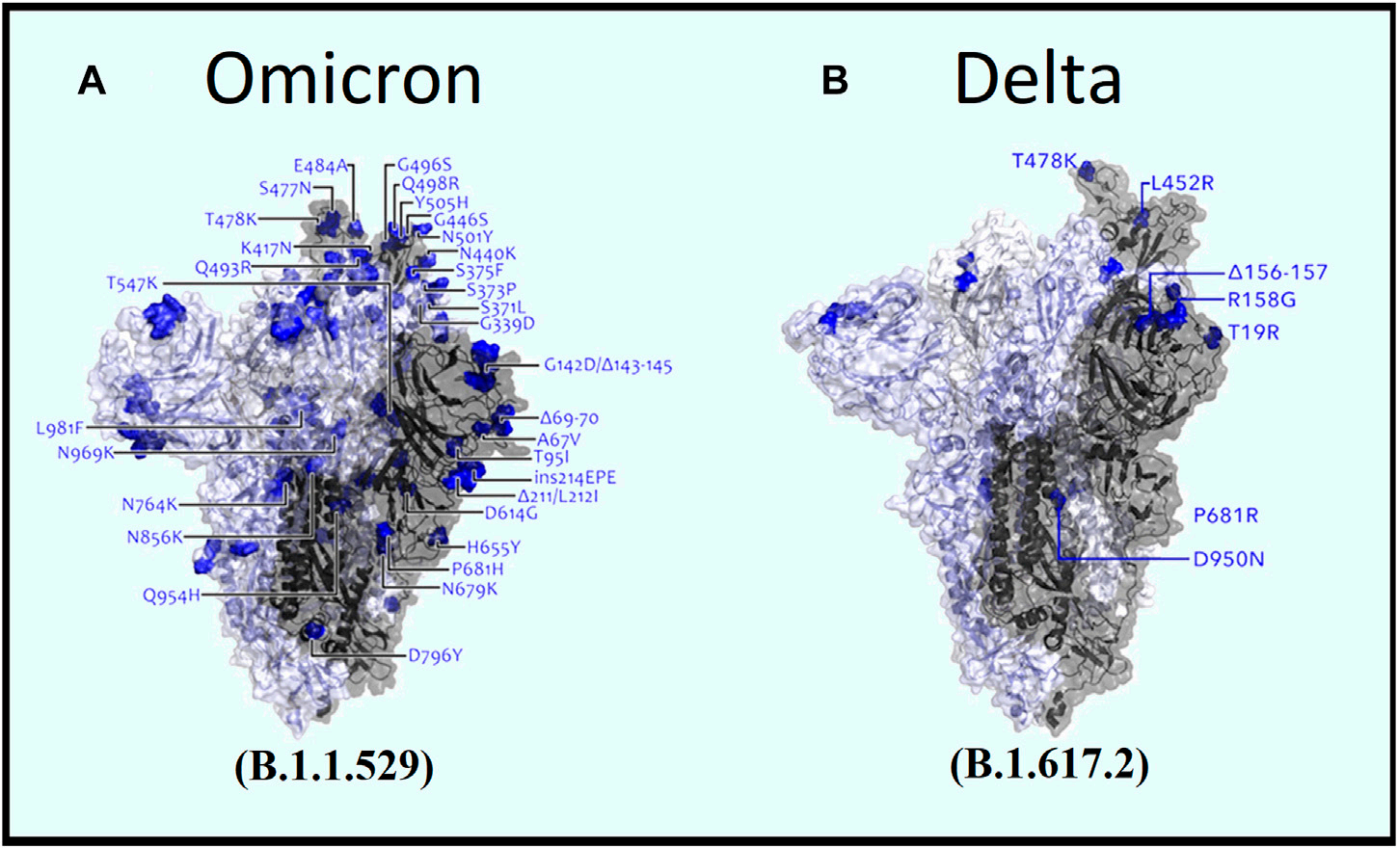
Comparative view of spike mutations within **(A)** Omicron and **(B)** Delta variants (Image source: Modified from COG-UK Mutation Explorer: http://sars2.cvr.gla.ac.uk/cog-uk).

By analyzing the common mutations among the 12 SARS-CoV-2 variants, our analysis found that the Omicron variant possessed the highest number of mutations in spike protein (Figures 4A, B), whereas the Delta variant possessed the lowest number (Table 3). While the Omicron variant showed an overall higher frequency of common mutations in N, ORF1a, ORF1b, ORF7, and ORF7b of the S protein, Alpha, Gamma, and Delta variants were exposed to numerous unique mutations (Figure 3A). Among these mutations, the Omicron variant had the highest number of unique mutations. Accordingly, the deleterious/non-synonymous mutation scores varied among variants (Figures 3B, D). A predominant score zone was observed for the Delta variant. The S protein of this variant (Omicron) was more virulent because of its immense unique-deleterious mutation score (Table 3).

### 3.3 B–cell epitope prediction

**FIGURE 5:**
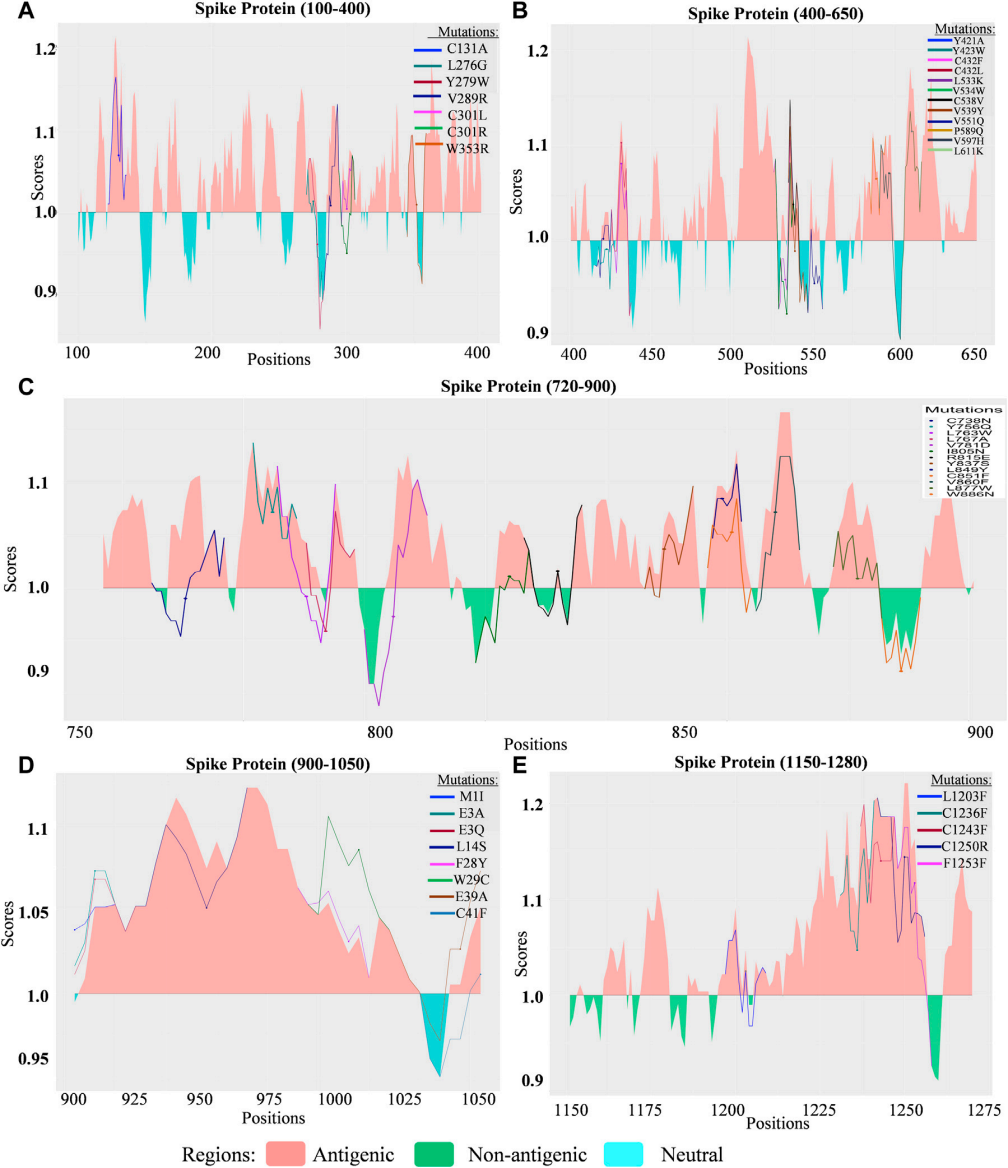
Comparison of the B cell epitope for spike (S) protein of SARS-CoV-2 Delta variant. **(A)** B cell epitope prediction score from 100 to 400 amino acids (aa) of the S protein. **(B)** B cell epitope prediction score from 400 to 650 aa of the S protein. **(C)** B cell epitope prediction score from 720 to 900 aa of S protein. **(D)** B cell epitope prediction score from 900 to 1050 aa of the S protein. **(E)** B cell epitope prediction score from 1150 to 1280 aa of the S protein.

The probability of altered proteins becoming B–cell epitopes (up and down-regulation of B cell immunological response) for specific detrimental mutations in the Delta variant was visualized using the “ggplot2” package of the R statistical environment (Figures 3, 4; Figure 5). The threshold value used to define the region was set to 1.00. The upper portion of the threshold value can be referred to as antigenic regions that are most likely to be an epitope for B cells, whereas the lower portion showed non-antigenicity for B cells.

The deleterious mutations for which the antigenic response decreased were considered significant, and an increase in the antigenic response represented unchanged, neutral, or insignificant mutations. The mutations for which the B cell response rose significantly in the antigenic portions were extremely beneficial to the host immune system. By contrast, a decrease in the scores in the antigenic region indicates a threat to the host immune system.

#### 3.3.1 Delta variant

##### 3.3.1.1 ORF1a

Out of 666 deleterious mutations detected in ORF1a, 281 had lower antigenic score. To present these on the graph, we further predicted T Cell epitopes. Mutations that passed both analyses (n = 37 mutations) were presented in five segments featuring positions 2200–2648, 2700–3190, 3300–3500, 3700–4000, and 4000–4300 (Supplementary Figure S1). Among these mutations, L2218D and F2598N, L2948T, L3754G, F4034Q, L4234S, and V4242W (Supplementary Figure S1) appeared to have the greatest impact on lowering antigenic scores.

##### 3.3.1.2 ORF1b

Among 262 deleterious mutations detected, 92 had a lower antigenic score for B cells. Based on the significance level in both B- and T Cell epitope prediction, the Y894M and L898D (Supplementary Figure S1) mutations were significant in both contexts.

##### 3.3.1.3 ORF3a

Out of 47 deleterious mutations detected, 18 occurred in the B cell epitope region of the ORF3a.Among theseI35T and Y107H (Supplementary Figure S1) mutations were found to be significant in B- and T Cell epitope prediction.

##### 3.3.1.4 ORF6

Of the 10 deleterious mutations detected in ORF6, I37T had a lower score.

##### 3.3.1.5 ORF7a

Of the 30 deleterious mutations found in ORF7a, 13 significant mutations were found. Six of these were the most significant, including I4T, V24F, C58F, C67F, V74F, and V82A. The C67F mutation occurring in the antigenic region had a lower score than the threshold value, indicating the region was a non-antigenic portion. Among the most significant mutations, V82A decreased the likelihood of being a B cell epitope and had a higher mutation count of 1,831 (Supplementary Figure S1). Similarly, the I4T mutation remained significant in both contexts.

##### 3.3.1.6 ORF7b

A total of eight deleterious mutations such as M1I, E3A, E3Q, L14S, F28Y, W29C, E39A, and C41F were analyzed (Supplementary Figure S1). Among these, L14S was the most significant mutation observed in the antigenic region, while C41F was found in the non-antigenic region. A positive antigenic response was detected due to six different mutations (M1I, E3A, E3Q, F28Y, W29C, and E39A) in ORF7b.

##### 3.3.1.7 ORF8

Out of the 22 deleterious mutations detected, five were found to be significant. The C37F mutation occurred in the antigenic region with a mutation count of 38 and lowered the likelihood of binding to B cells (Supplementary Figure S1). Furthermore, the I10N, C102F, C102Y, and F120A mutations occurred in the antigenic portion and contributed to the negative antigenic response. However, none of the mutations dragged the antigenic region into a non-antigenic region.

##### 3.3.1.8 ORF9b

Among the 22 deleterious mutations detected in ORF9b, five occurring in the antigenic regions may contribute to lowering the likelihood of binding to B cells. These five mutations (P3S, A11S, L14F, I45T, L52P, and V76F), along with others, are presented in Supplementary Figure S1. Among these, P3S and V76F attribute the antigenic region to be non-antigenic. However, L52P was significant in both B- and T cells.

##### 3.3.1.9 Envelope protein

Three deleterious mutations were identified in the envelope protein. Among these, V58F was the most significant, as it decreased the score and lowered the immune response of B cells in the antigenic region (Supplementary Figure S1). The other two mutations were observed to increase the antigenic score, making the envelope more susceptible to the adaptive immune system (Supplementary Figure S1).

##### 3.3.1.10 Membrane glycoprotein

Of the three deleterious mutations detected in membrane protein, mutations at residue position 82 were found to be significant, as they lowered the antigenic response of the antigenic portion. As observed, the replacement of isoleucine with threonine had a much greater effect than serine. In addition to this mutation, threonine had a higher mutational count of 1,864 (Supplementary Figure S1).

##### 3.3.1.11 Nucleocapsid proteins

Deleterious mutations are presented in the context of B cell responses. Three mutations (e.g., K248M, R203M, and P168S) were highly significant out of 16 deleterious mutations found in the nucleocapsid. K248M and P168S mutations occurred in antigenic regions and contributed to lowering the immune response to non-antigenic regions, whereas R203M occurred in the non-antigenic portion with a high mutation count of 1,868 (Supplementary Figure S1).

##### 3.3.1.12 Spike (S) protein

Significant mutations detected in Sprotein were divided into five sections (Figures 5A–E). Out of 116 mutations identified, 48 mutations were deleterious, occurring in the antigenic region and lowering the score. Thirteen mutations, including C301R (Figure 5A), Y423W, L533K, V539Y, V551Q (Figure 5B), C738N, L763W, V781D (Figure 5C), V915W, V915S, L916S, I923W, and A944R (Figure 5D) were found to drag the antigenic portion to a non-antigenic region of the spike protein. These mutations had the highest occurrence compared with other mutations. For example, V539Y, C738N, and L763W mutations were found to have occurred 16-times while A944R mutation was found 15-times, Y423W for 13-times, V915W, L916S, and I923W 12-times and V915S occurred 3-times.

#### 3.3.2 Omicron variant

**FIGURE 6:**
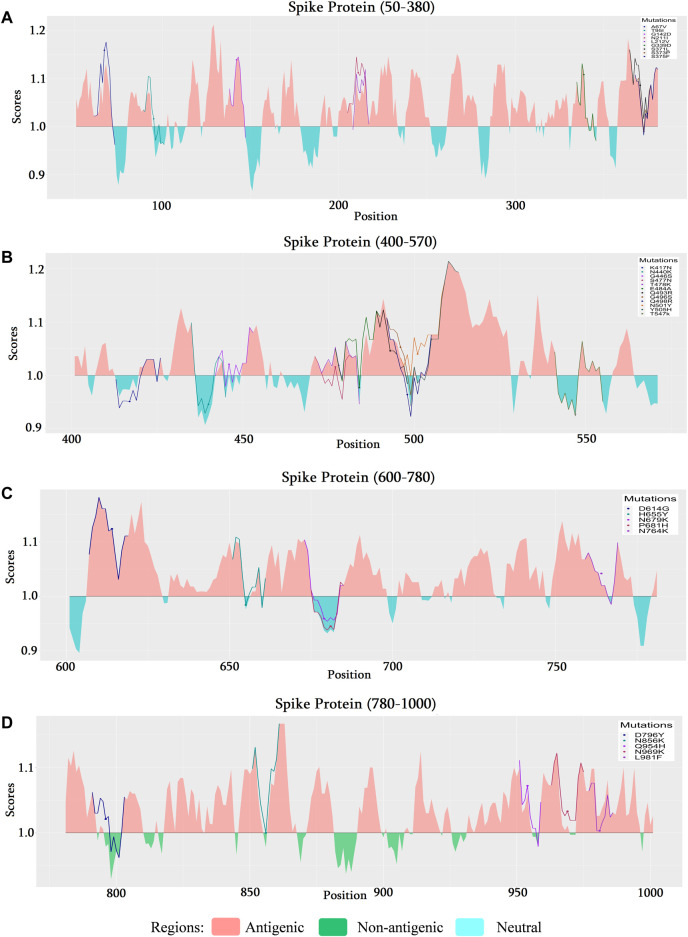
Comparison of the B cell epitope for spike (S) protein of SARS-CoV-2 omicron variant. **(A)** B cell epitope prediction score from 50 to 380 amino acids (aa) of the S protein. **(B)** B cell epitope prediction score from 400 to 570 aa of the S protein. **(C)** B cell epitope prediction score from 600–780 to 900 aa of S protein. **(D)** B cell epitope prediction score from 780 to 1000 aa of the S protein.

As mutational analysis and B cell epitope prediction on delta variant directs the most significance towards spike protein, the analysis narrowed down to explore mutations of omicron on spike protein too. The mutational analysis of the top 100 mutations found most deleterious are selected for further analysis (Supplementary Table S8). Among these A67V, G142D, N211I, L212V, G339D, S371L, S375F, G446S, S477N, T478K, Q493R, G496S, Y505H, D614G, N764K, Q954H, N969K, L981F mutations found to be occurring in antigenic regions of spike protein (Figure 6). Its is a noticeable result that along with the other mutations of delta variant, additional mutations G339D (Figure 6A), S477N (Figure 6B), Q493R (Figure 6B), and Y505H (Figure 6B), occurring in RBD region of spike protein, shows significance in the context of B cell epitope prediction.

### 3.4 T Cell epitope prediction

CD4 T Cell immunogenicity prediction tool gives scores of segments from the peptides which can be identified as immunogenic based on a threshold value (50%). Most of the significant T Cell epitope mutation scores were predicted as less than 50 (Supplementary Table S1). An increment of the combined score for a mutant was taken into concern as it depicts susceptibility towards survival into host immunogenic conditions. Envelope protein mutations V58F and R61L were observed to have a significant score (<50%) in the two epitopic segments (Supplementary Table S1). It was also predicted that the combined score increase is noticeable in the epitope region covering positions 51–65 (50% in this case). However, mutations exhibited a decreased score in the region 56–70, which indicated greater immunogenicity of T Cell epitopes. Among the 281 mutations that lowered B cell epitope potency, 50 mutations were found to significantly lower T Cell epitope potency by crossing the threshold. Therefore, the F2598N, L2948T, L3754G, F4034Q, L4234S, and V4242W mutations were found to be the most significant in both B- and T Cell epitope analyses (Supplementary Table S1).

Amidst the identified 92 mutations from B cell epitope prediction, 25 were found to lower the immune potency of T cells. while 11 mutations suppressed T Cell responses. The L898D and Y894M mutations were most significant for B- and T cells. For ORF3a, the I35T and Y107H mutations appeared to cross the threshold. ORF6 and ORF8 did not show many similarities to the expected result, suggesting that the mutations are safe for the host. For ORF7a, ORF7b, and ORF9b, the mutations I4T, L14S, and L52P showed the expected significance by crossing the threshold. In T Cell epitope prediction V911Y, V915S, V915W, L916S, A924Q, and I923W increased the combined score over the threshold values. However, V915W, V915S, L916S, and I923W were the most significant mutations in both analyses (Supplementary Table S1).

As, the analysis suggested, additional six mutations of omicron variant A67V, G142D, S371L, S373P, S375F, and L981F occur in the positions that were T Cell responsive in the wild variant. Among the six A67V, G142D, and L981F are seen (Supplementary Table S4) to induce the increase of combined score. L981F is the most important in this context as it crosses the threshold value indicating that the region is becoming less responsive towards CD4 T cells.

### 3.5 Effects of missense mutations on protein stability and protein-protein binding affinity

#### 3.5.1 Delta variant

Physical characteristics of two mutations (V58F and R61L) of the envelope protein were collected from the COVID-3D database. However, both mutations were destabilizing, along with decreased molecular flexibility (Supplementary Table S1). Both I82S and I82T mutations of membrane protein, as well as P168S mutation of nucleocapsid protein, are destabilizing and contribute to increased molecular flexibility of spike protein (Supplementary Table S2). The I35T mutation had a more destabilizing effect on ORF3a than Y107H with increased molecular flexibility (Supplementary Table S2).

**FIGURE 7:**
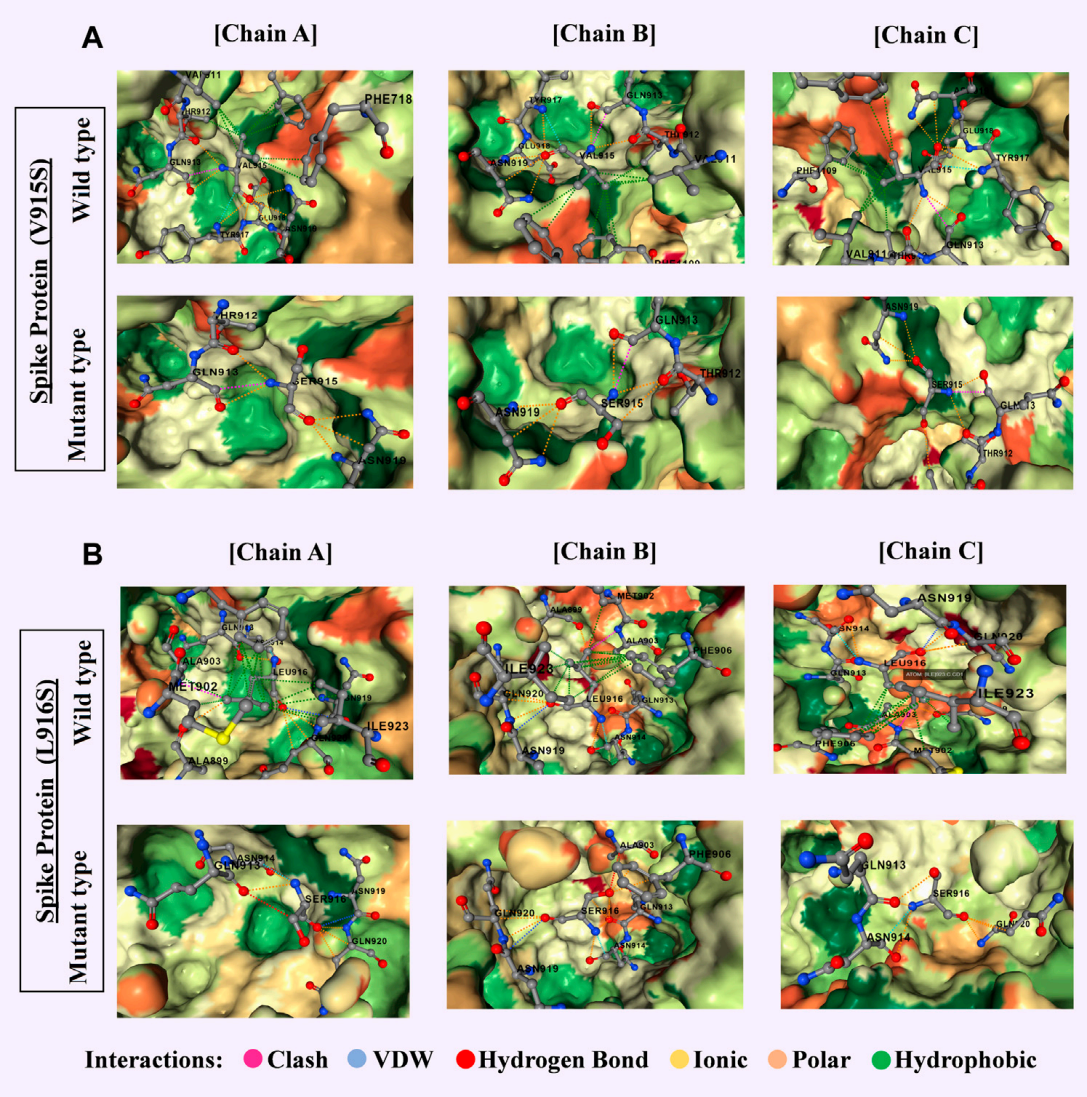
Most significant two mutations of spike proteins of delta variant with a comparative overview of molecular interaction **(A)** V915S-chain A-absence of 9 hydrophobic bonds, 2 polar bonds, and 1 Vander Waals bond in the mutein. Chain B- the absence of 9 hydrophobic bonds, 1 polar bond, and 1 Vander Waals bond in the mutein. Chain C- absence of 9 hydrophobic bonds, 1 polar bond, and 1 Vander Waals bond in the mutein. **(B)** L916S-chain A-the absence of 12 hydrophobic bonds in the mutein. Chain B- absence of 17 hydrophobic bonds, increase of 3 polar bonds and 1 hydrogen bond, decrease of clash in the mutein. Chain C- the absence of around 10 hydrophobic bonds, 1 carbonyl bond, and 1 clash in the mutein. Clashes are defined as unfavorable interactions where atoms are too close together.

The I4T mutation in ORF7a, being destabilized and increased molecular flexibility, showed significance in B and T Cell epitope prediction too. The L14S mutation of ORF7b was also significant, as it was found to be a destabilizing mutation that increases molecular flexibility (Supplementary Table S2). A detailed graphic overview of the molecular interactions of these mutations is shown in Figure 7.

#### 3.5.2 Omicron variant

Except for N440K, Q493R, and Q498R, all other 12 mutations (G339D, S371L, S373P, S375F, K417N, G446S, S477N, T478K, E484A, G496S, N501Y, Y505H) on receptor binding domain (RBD) were found as destabilizing indicating that these mutations are most likely to go through further several mutations (Supplementary Table S6). Among these, G339D (Figure 6A), S477N, and Q493R occurring in the B cell epitope region and Y505H occurring in the non-epitope region (Figure 6B) induce less likeliness towards B cell as suggested B cell epitope prediction section. These destabilizing mutations are of concern as further mutations on the same position could turn the protein less immunogenic and more specific toward the host receptors.

**FIGURE 8:**
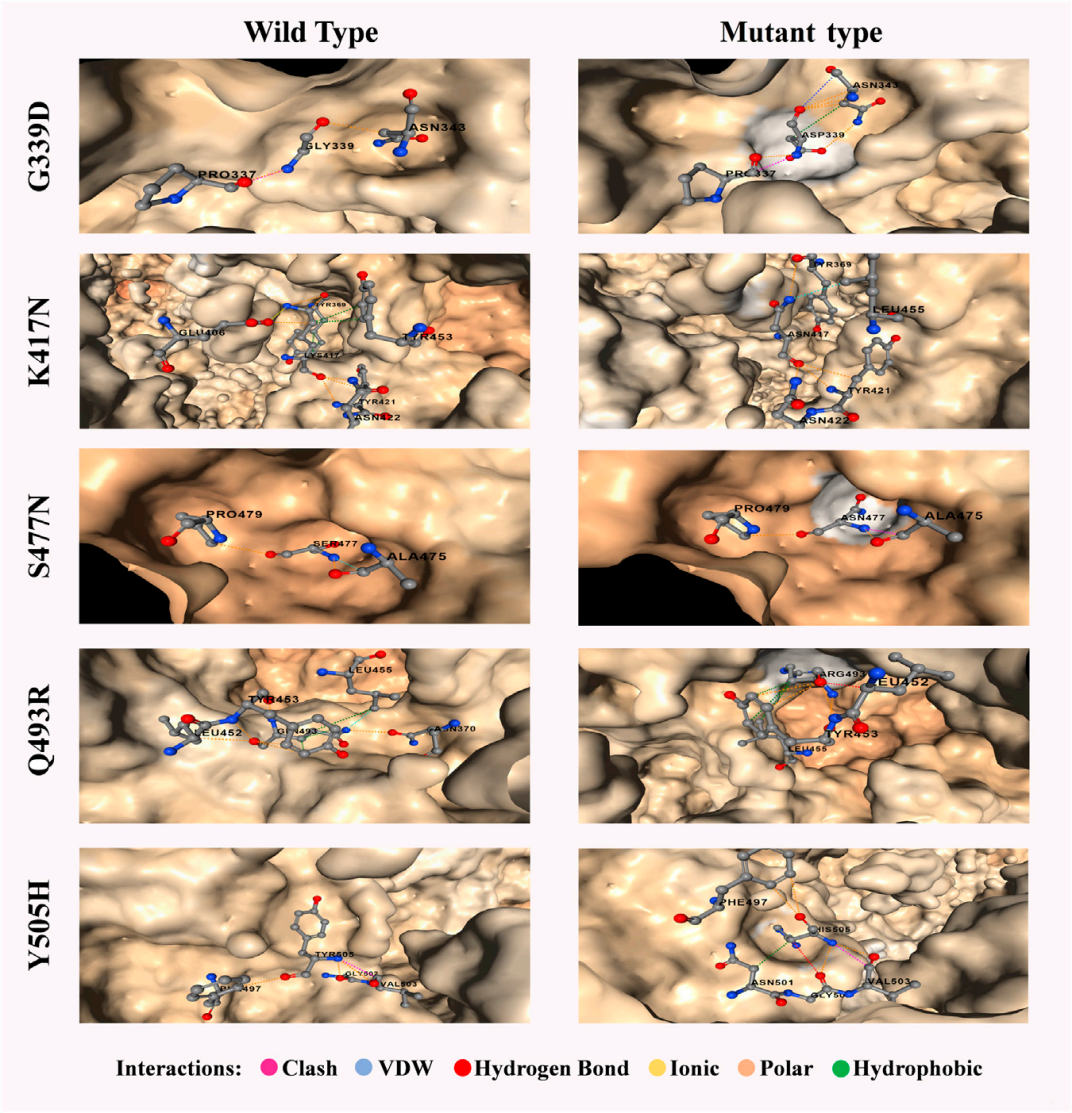
Most significant five mutations of spike proteins of the omicron variant with a comparative overview of molecular interaction. A wild variant of G339D has 1 clash and 2 polar bonds whereas the mutein has an additional 3 polar, 1 hydrophobic and 1 van der waals bonds. Accordingly, wild variant of K417N has 6 polar, 1 ionic and 5 hydrophobic bond whereas the mutein has an additional 2 polar and 1 van der waals bond but lacks ionic and hydrophobic bonds. Wild variant of S477N has 2 polar and 1 van der waals bond whereas the mutein lacks van der waals bond but has an additional 1 clash. Wild variant of Q493R has 6 polar, 1 van der waals, and 3 hydrophobic bond whereas the mutein lacks van der waals bond and 2 polar bonds but has an additional 1 hydrogen bond. Finally, Y505H has 3 polar and 1 clash which results in additional 1 polar, 1 hydrogen, 1 van der waals, and 1 hydrophobic bonds with the lackings of the clash. Clashes are defined as unfavorable interactions where atoms are too close together.

The increasing affinity of protein-protein binding (PPB) phenomena could be an issue to focus as this may enable the spike protein to bind host receptor with higher affinity. Therefore, in this context, G339D, N440K, S477N, Q498R, and N501Y (increase affinity in chain C) mutations could be used to explore in the future as each of these mutations contributes to increasing PPB affinity (Supplementary Table S5). It is a must to mention that both G339D (Figure 6A) and S477N (Figure 6B) also have significant respect for the B cell epitope prediction tool. A comparison of the molecular interaction of these mutations with the wild variant could ease the mind with a proper understanding of the impact of mutations on the protein-protein interaction (Figure 8).

### 3.6 Machine learning validation

Logistic regression, linear discriminant analysis, and artificial networks have similar kind of results aligned with each other in the context of deleterious and neural mutation perspectives (Table 4).

**TABLE 4:** Prediction of deleterious mutation depending on four classifiers: Logistic Regression, Linear Discriminant Analysis, Artificial Neural Network, and Support Vector Machine.

	True values								
		Logistic regression		Linear discriminant analysis		Artificial neural network		Support vector machine	
		*D	*N	*D	*N	*D	*N	*D	*N
Predicted Values	Deleterious	29	31	29	24	32	23	25	38
	Neutral	6	95	6	100	3	101	10	86

*D, Deleterious mutation *N, Neutral mutation.

Whereas the result from the support vector machine classifier slightly differed from the result of rest of the models. However, all the models ensure at least 75% accuracy compared to the deleterious tool prediction and scenario. Therefore, in that context, it can be stated that machine learning models can validate the results at least 75% of cases and artificial neural network can validate the result up to 85% which is enough to maintain a balanced error-free mutational analysis (Table 5).

**TABLE 5:** Comparative analysis of classification quality (accuracy) for classifiers trained without transformation (in initial feature space) and with transformation (in transformed space from the use of a neutral network) on mutational data.

	Accuracy	Kappa	Sensitivity	Specificity	Auroc	Mcnemar’s test *p*-value
LR	0.8151	0.0694	0.8286	0.6984	0.5635	<0.001
LDA	0.8214	0.0742	0.8286	0.8065	0.5675	<0.001
ANN	0.8559	0.0498	0.9143	0.7855	0.5499	<0.001
SVM	0.7597	0.1134	0.4286	0.7968	0.7513	<0.001

As it is evident from Tables 4, 5, the classifiers can reach, on average, 80.5% accuracy in the initial feature space and 74.5% sensitivity can be attained most of the time. For Logistic Regression, Linear Discriminant analysis the classification in transformed feature space leads to more accurate predictions. The significance level is undoubtedly confirmed over the test (*p* < 0.0001). Therefore, the deleterious mutations can be confirmed to a most significant percentage maintaining all the machine learning validation. The ROC curve estimated from the result section portraited a more transparent and visually vivid explanation with precise interpretation (Supplementary Figure S4).

## 4 Discussion

Since the first emergence of SARS-CoV-2 in late December 2019, multiple genomic variants have emerged, among which the Delta variant has been declared a ‘variant of concern’ (VOC) until November 2021 due to distinct characteristics (Araf et al., 2022; Mohapatra et al., 2022). The first Omicron variant (B. 1.1. 529) was identified on 09 November 2021 from a clinical sample that possessed comparatively higher mutations than other variants. The VOC was attributed to higher transmissibility with a severe disease course, decreased treatment efficacy, and many other concerning features by the Centers for Illness Control and Prevention (CDC) (https://www.cdc.gov/coronavirus/2019-ncov/index.html). However, The World Health Organization designated the Omicron variantB.1.1.529 as a VOC on 26 November 2021. A recent study reported that protection against the Omicron variant was only moderately conferred by the primary COVID-19 vaccination and prior SARS-CoV-2 infections (Chin et al., 2022). Although protection against Omicron infection was greatly enhanced by booster dosage, it gradually reduced with time (Andeweg et al., 2022). Mutation in the SARS-CoV-2 is a continuous process leading to multiple variant introductions. Though the latest SARS-CoV-2 variant’s infectivity, prevalence, and severity are still unknown, investigations along with genomics analysis should be an ongoing process to get every detail to recommend efficient ways to prevent the upcoming surge (Araf et al., 2022).

New variants of SARS-CoV-2 had been observed during the pandemic. According to Majumdar and Niyogi. (2020), mutations in the ORF3a protein of SARS-CoV-2infectionis associated with a high mortality rate. Previous research has indicated that the D614G mutation of SARS-CoV-2 plays a role in the severity and mortality of COVID-19 patients, along with other factors, especially age and co-morbidity (Cong et al., 2020; Dutta et al., 2020; Grubaugh et al., 2020).

In this study, we filtered out 16,954 unique and common mutations from 10,531 sequences for 12 strains (Table 1). These unique mutations being present in a variant could be used to explain why that particular variant is much more transmissible than the previous one. For example, among 100 mutations of omicron in spike protein around 30 mutations were found to be unique (Supplementary Table S8) giving one of the clue that why this variant is more transmissible than delta. However, the presence of a destabilized common mutation in a new variant could be detrimental for future variants as these mutations tend to mutate over time. Based on these detrimental impacts, we characterized the mutations in neutral and deleterious sections. The mutations that is contributing to the detrimental impact on the host are therefore defined as deleterious and on the contrary, the other is defined as neutral having no significant impact on the host. All the deleterious mutations and their score predicted from some specific mutational analysis tools were further validated by four machine learning models: Logistic Regression, Linear Discriminant Analysis, Artificial Neural Network and Support Vector Machine (Tables 4, 5). At most 85% match score had been achieved by Artificial Neural Network while the lowest similarity scenario was achieved by a Support Vector Machine (Table 5). The significance level is confirmed over the test at its best (*p* < 0.0001).

Analysis of the unique mutation frequency of each variant indicated that the Omicron and Delta variants showed more frequent deleterious mutations. Surface glycoproteins also showed the notable feature of a unique mutation. Overall, the Omicron variant covered 512 unique mutations in its S protein, whereas Delta, Alpha, Gamma, and Kappa variants covered 352, 62, 96, and 126 unique mutations, respectively (Table 2).

The Omicron variant changes the structural pattern of the spike protein more rapidly than expected (Figure 8), which can be well explained by its rapidly transmissible nature. Various studies have focused on mutations in spike proteins (Duan et al., 2020; Huang et al., 2020; Xie et al., 2020). Therefore, mutations that occur in the surface glycoprotein may be of concern (Islam et al., 2020).

In addition, the neutral and deleterious mutation scores (Supplementary Table S7) of different variants were analyzed. Neutral or synonymous mutations were observed among all variants (Lucas et al., 2008; Shah et al., 2020) in their spike proteins (Table 2). The highest number of neutral mutations was observed in the Omicron variant, whereas in Alpha, Gamma, Epsilon, Eta, and Kappa variants, 155, 200, 118, 129, and 316 neutral mutations, respectively (Table 1). Accordingly, more deleterious mutations were observed in the spike protein, indicating the attempts of the spike protein to break the immune defense system of the host (Table 2).

In this study, we took a computational approach to analyze deleterious mutations among common and unique mutations in the variants. Accordingly, we have compared all the mutations in different variants by some specific markers (deleterious score, average deleterious score, average deleterious mutations per sequence of different variants, and mutations severely affecting the stability of the protein). After considering all of the factors it was estimated that the degree of deleterious mutations was far higher in the Omicron variant compared to other variants. While Omicron variants showed the highest mutation score pattern for both common and unique mutations, the Delta variant showed the second-highest pattern. A comparative analysis of the top 184 unique-deleterious mutations in Omicron revealed it as one of the most transmissible variants (Table 3), containing more deleterious mutations than that of Delta. The Omicron variant showed a high degree of deleterious mutations in S, ORF1a, ORF7a, ORF 8, and ORF 9b. This result indicated that the effective and non-synonymous mutations scaled up the Omicron variant to be more virulent and transmissible. The Delta variant, originating in India had already claimed numerous lives in the previous year, while the Omicron variant had been shown to infect more included as hospital cases (Sigal et al., 2022).

The spike protein of the Omicron variant showed a high frequency of deleterious mutations (Table 3). The deleterious mutation in this protein was cross-checked using four tools (Predict SNP, SIFT, PolyPhen2, and PROVEAN). With a high deleterious score, the surface glycoprotein of the Omicron variant confirmed its more virulent and transmissible nature. The heatmap of unique mutation scores of different proteins within different variants indicated that the Omicron variant had the highest possible deleterious mutation in its spike protein compared with the other variants (Figure 3). Moreover, other protein portions of the Omicron variant showed more non-synonymous mutations responsible for protein function alteration and immune escape mechanisms (Figure 8 and Supplementary Table S4).

From these observations, we can conclude that mutations of the omicron variant in the spike protein have the greatest impact. V915S and L916S are both destabilizing mutations that occur with a serine residue. Both have been shown to lower the likelihood of binding with B- (Figure 5D) and T cells (Supplementary Table S1), increase molecular flexibility, and decrease affinity (Supplementary Table S3). In ORF3a (I35T, Y107H), both mutations are of concern considering B and T cells (Supplementary Figure S1), although I35T is the most destabilized with increasing molecular flexibility (Supplementary Table S2). ORF7a (I4T), ORF7b (L14S), and ORF9b (L52P) proteins have one destabilizing mutation (Supplementary Table S2) that increases the likelihood of binding with B (Supplementary Figure S1) and T cells (Supplementary Table S1) and also increases molecular flexibility (Supplementary Table S2). ORF1a and ORF1b proteins give concerned mutation lists considering both B- (Supplementary Figure S1) and T Cell (Supplementary Table S2) epitope prediction. Mutations in membrane protein (I82T), nucleocapsid protein (K248M and P168S), ORF6 (I37T), and ORF8 (C37F, I10N, C102F, C102Y, and F120A) were significant for lowering B cell epitope potency (Supplementary Figure S1). Mutations in the envelope (V58F) were also significant for B cell epitope prediction (Supplementary Figure S1) while providing mixed results for T Cell epitope prediction (increase in score in one peptide but decrease in other peptide portion) (Supplementary Table S2) Asghar et al., 2022. These results from our study corroborated the results of many of the previous research.

In comparison, 10 mutations (W353R, Y421A, Y423W, C432F, C432L, L533K, V534W, C538V, V539Y) were found to be significant in RBD region ranging from 319 to 541 (Kumar et al., 2022) of the spike protein of delta variant in context of B cell epitope prediction (Figures 5A, B) whereas in omicron variant’s spike protein additional four mutations (G339D, S477N, Q493R, and Y505H) (Figures 6A, B) were enlisted confirming the significance level of omicron spreading.

Although the T Cell epitope prediction context does not give much of a clue about the deleterious effects of mutations on the RBD region, both variant stability, and protein-protein binding affinity gives some interesting aspect. As seen for the omicron variant most destructive mutations that affect the protein stability (Supplementary Table S6) reside between the residues 339–505 featuring mutations G339D, S371L, S373P, S375F, K417N, G446S, S477N, T478K, E484A, G496S, N501Y, Y505H, each of which are in RBD. But in the case of the delta, these effects were observed by mutations V911Y, V915S, V915W, V916S, I923W, and A924Q which is in the heptad region (HR1) and ranged from 902 to 952 (Xu et al., 2004). For protein-protein binding affinity, the most significant value is also observed between 911–924 residues in the case of the delta variant (Supplementary Table S3) and 339–505 residues for the omicron variant (Supplementary Table S5).

While several studies have shown that the Alpha variant is associated with an overall higher case fatality rate than the original lineages (Brainard et al., 2022), only a few studies indicated Delta and Omicron variants (Challen et al., 2021; Nyberg et al., 2022). In a cohort study in the Netherlands, van Gils et al. (2022) reported that vaccines were less neutralized by the Omicron variant even after booster dosing. According to Zhang et al. (2022) two-dose mRNA-1273 and BNT162b2 vaccines were able to neutralize against Alpha, Beta, Gamma, and Delta variants but after 6 months, antibodies declined for BNT162b2. Both the two vaccines, mRNA-1273 and BNT162b2 were found weakly active against Omicron for neutralizing antibodies.

Despite few antiviral drugs and vaccines available on the market to control the spread of infections, new SARS-CoV-2 variants continue to emerge, making it difficult to combat without complete immunization (M. Hossain et al., 2021; Sakib et al., 2021). The heatmap in our study (Figure 3) revealed that the Omicron variant had more common mutations in comparison with other variants, where most of the common mutations of the Omicron variant are deleterious (Table 1), causing alterations in protein function. We analyzed the association of host immune response with each deleterious mutation of the Omicron variant (Figure 6) as it is a variant of concern and revealed important outcomes. The deleterious mutations of the Omicron variant were cross-checked and the B cell responses to the different deleterious mutations of different proteins were analyzed (Figure 6). Most of the deleterious mutations were responsible for the downregulation of the immune response, especially the mutations in the ORF1a, ORF3a, ORF7a, ORF8, and ORF9b regions (Supplementary Figure S1; Supplementary Table S1). The spike protein had the greatest impact on suppressing the host adaptive immune response (Figures 5, 6; Supplementary Tables S1, S4). This indicates a comparative graphical analysis of the special immunosuppressive capability of all proteins, especially within the spike protein for the Omicron variant. The specific mutations responsible for the highest immunosuppressive nature were further cross-checked with their respective mutation frequencies suggesting they occurred in a repeated manner which makes them too virulent to challenge humanity for their existence.

## 5 Conclusion

The presence of unique and common deleterious mutations in delta and omicron suggests the reason for their aggressive virulence nature which has compelled the human being to face an epidemic in this modern era. Observing all the mutations of both delta and omicron variants, our study concludes that among all proteins spike protein is the most significant for both delta and omicron variants. In the delta, the significance has been drawn towards the heptad region as the most significant mutations are V911Y, V915S, V915W, L916S, I923W, and A924Q. Whereas, for omicron, the attention moves towards mutations G339D, K417N, S477N, Q493R, and Y505H which reflects the RBD region. This explains that although the delta variant is more deadly, omicron has its obvious ability to spread faster.

## 6 Future prospects

The prediction of the epitope of B cells and T cells does not wholly ensure the clinical conditions as it’s based on a computational method. It’s a guide for researchers to study specific mutations. Further study could be explored on the mutations to determine the effect on the epitopic region. The changes in molecular interactions are needed to study further to find out the chemistry of the mutein that increases the sustainability of an emergence variant.

## Data Availability

The datasets presented in this study can be found in online repositories. The names of the repository/repositories and accession number(s) can be found in the article/Supplementary Material.

## Supplementary material

SUPPLEMENTARY TABLE S1 T cell epitope prediction score for all the mutations of different proteins of SARS CoV-2 genome (Delta Variant).

SUPPLEMENTARY TABLE S2 Effects of missense mutations on different deleterious mutations of several proteins (Delta Variant).

SUPPLEMENTARY TABLE S3 Effects of missense mutations on different deleterious mutations of Spike Proteins (Delta Variant).

SUPPLEMENTARY TABLE S4 T cell epitope prediction score for all the mutations of different proteins of the SARS CoV-2 genome (Omicron Variant).

SUPPLEMENTARY TABLE S5 Effects of missense mutations on protein-protein binding affinity.

SUPPLEMENTARY TABLE S6 Effects of missense mutations on protein stability.

SUPPLEMENTARY TABLE S7 Learning (left) and Testing (right) dataset for machine learning validation.

SUPPLEMENTARY TABLE S8 All neutral and deleterious mutations for Delta and Omicron variant.

SUPPLEMENTARY TABLE S9 All of the SARS-CoV-2 Whole Genome Sequences list.
