# Supplementary material for: Variant-specific deleterious mutations in the SARS-CoV-2 genome reveal immune responses and potentials for prophylactic vaccine development
Source: Front Pharmacol. 2023 Feb 7;14:1090717. doi: 10.3389/fphar.2023.1090717 (PMC9941545; doi:10.3389/fphar.2023.1090717)
Supplement: Supplementary file 1 [file DataSheet2.pdf]

SUPPLEMENTARY TABLE S9

Sequences Derived From GISAID

Data Availability

GISAID Identifier:	EPI_SET_221230br
doi:	10.55876/gis8.221230br

All genome sequences and associated metadata in this dataset are published in GISAID's EpiCoV database. To view the contributors of each individual sequence with details such as accession number, Virus name, Collection date, Originating Lab and Submitting Lab and the list of Authors, visit 10.55876/gis8.221230br

Data Snapshot

- EPI_SET_221230br is composed of 220,652 individual genome sequences.
- The collection dates range from 2020-01-08 to 2022-01-31;
- Data were collected in 155 countries and territories;
- All sequences in this dataset are compared relative to hCoV-19/Wuhan/WIV04/2019 (WIV04), the official reference sequence employed by GISAID (EPI_ISL_402124). Learn more at https://gisaid.org/WIV04.
