# Supplementary material for: Variant-specific deleterious mutations in the SARS-CoV-2 genome reveal immune responses and potentials for prophylactic vaccine development
Source: Front Pharmacol. 2023 Feb 7;14:1090717. doi: 10.3389/fphar.2023.1090717 (PMC9941545; doi:10.3389/fphar.2023.1090717)

Machine Learning Validation

Logistic Regression

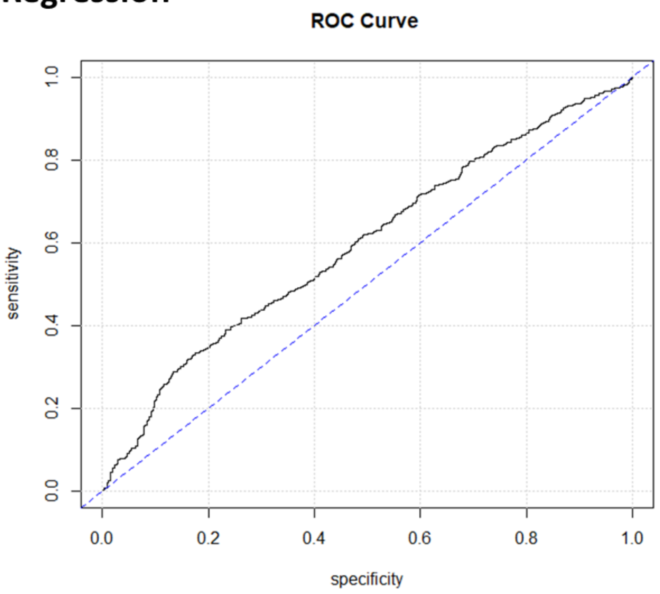

Linear Discriminant Analysis

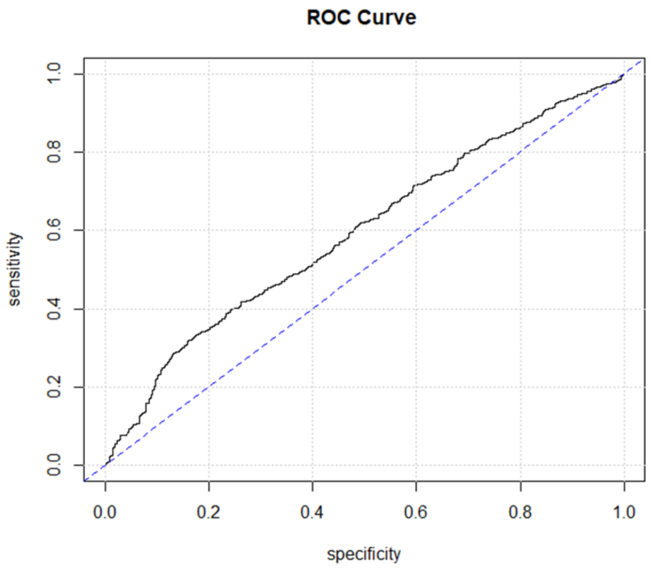

Artificial Neural Network

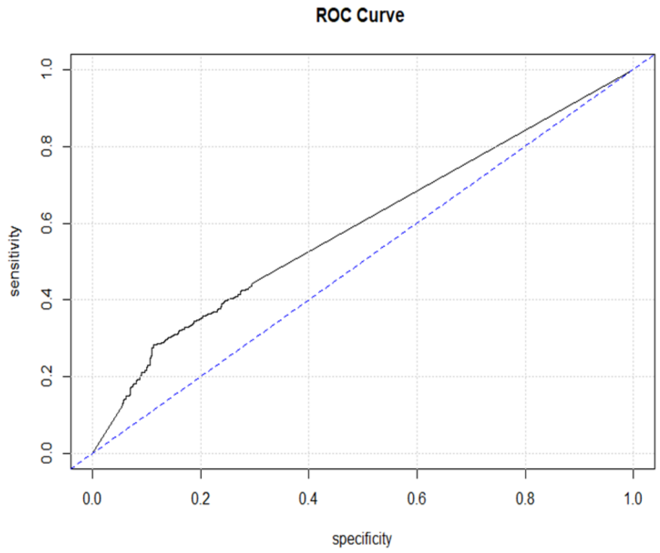

Support Vector Machine

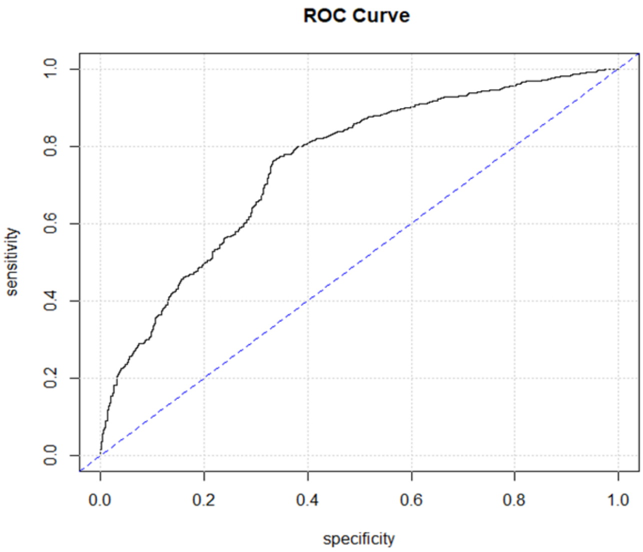

Supplement: Supplementary file 2 [file Image4.pdf]
