# Supplementary material for: Variant-specific deleterious mutations in the SARS-CoV-2 genome reveal immune responses and potentials for prophylactic vaccine development
Source: Front Pharmacol. 2023 Feb 7;14:1090717. doi: 10.3389/fphar.2023.1090717 (PMC9941545; doi:10.3389/fphar.2023.1090717)
Supplement: Supplementary file 3 [file Image2.pdf]

Effect of missense mutation on protein-protein interaction for Delta variant

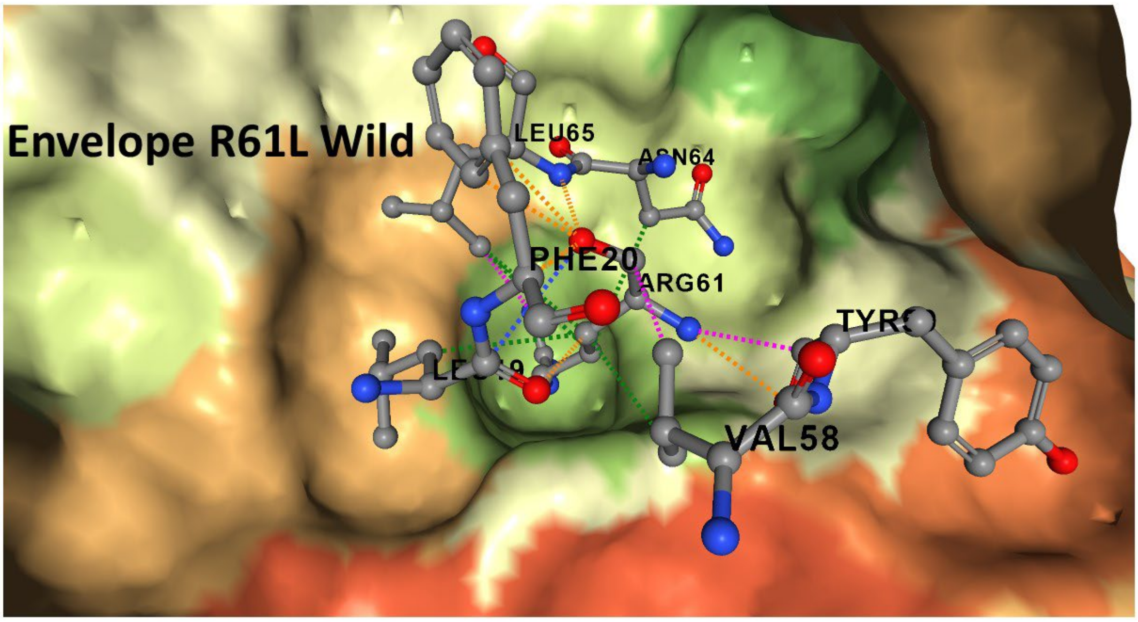

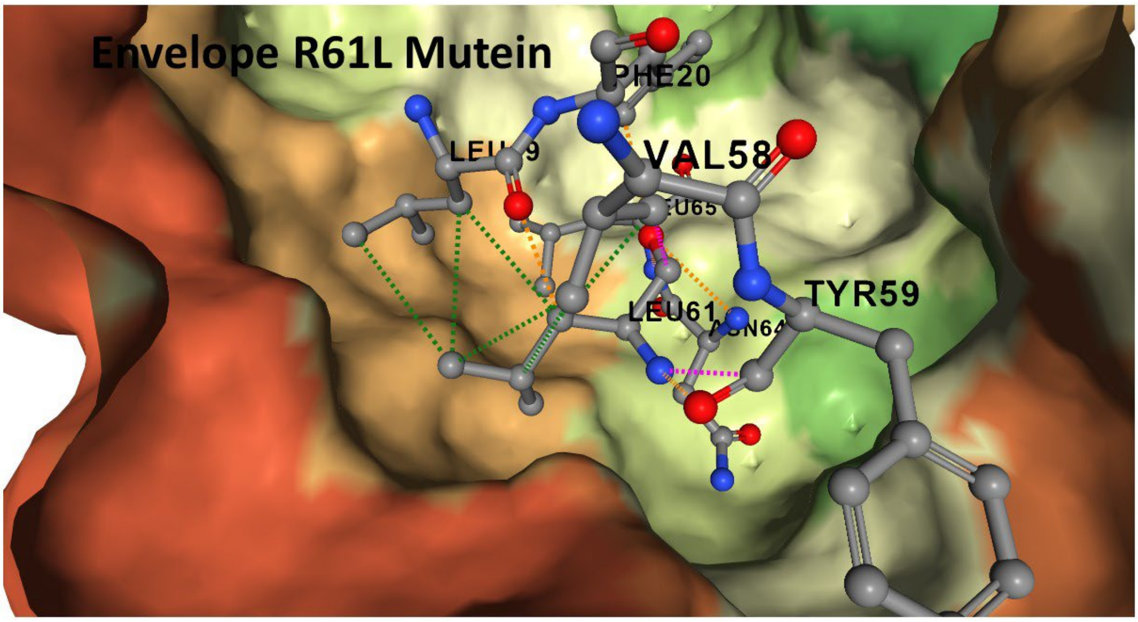
Envelope R61L Mutein
PHE20
VAL58
TYR59
LEU61

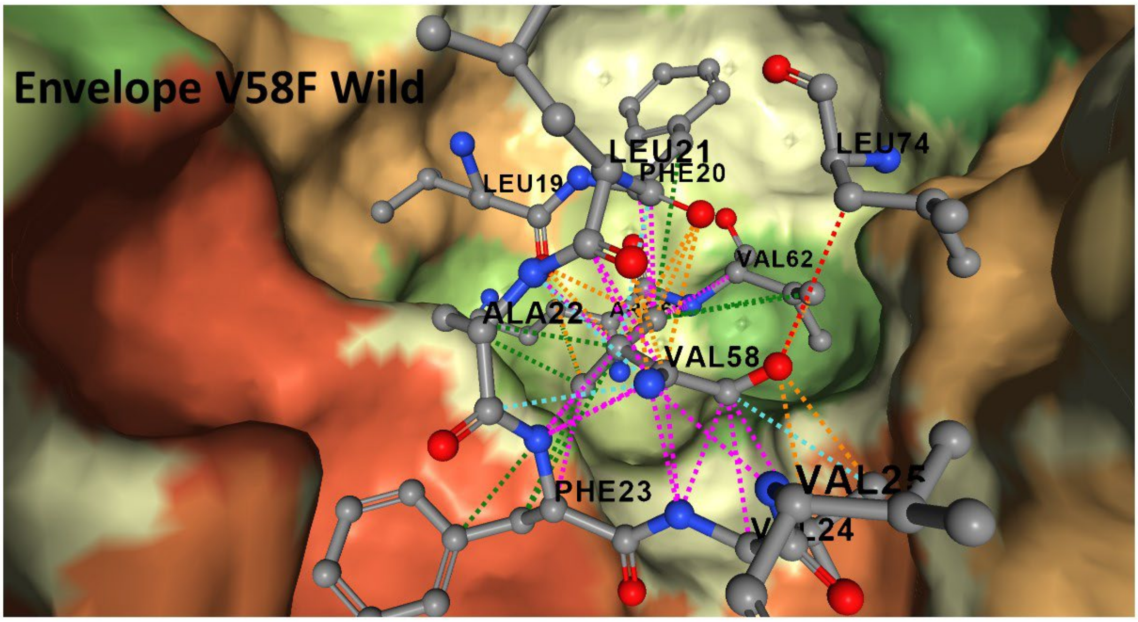
Envelope V58F Wild
LEU21
PHE20
LEU19
LEU74
VAL62
ALA22
VAL58
PHE23
VAL24

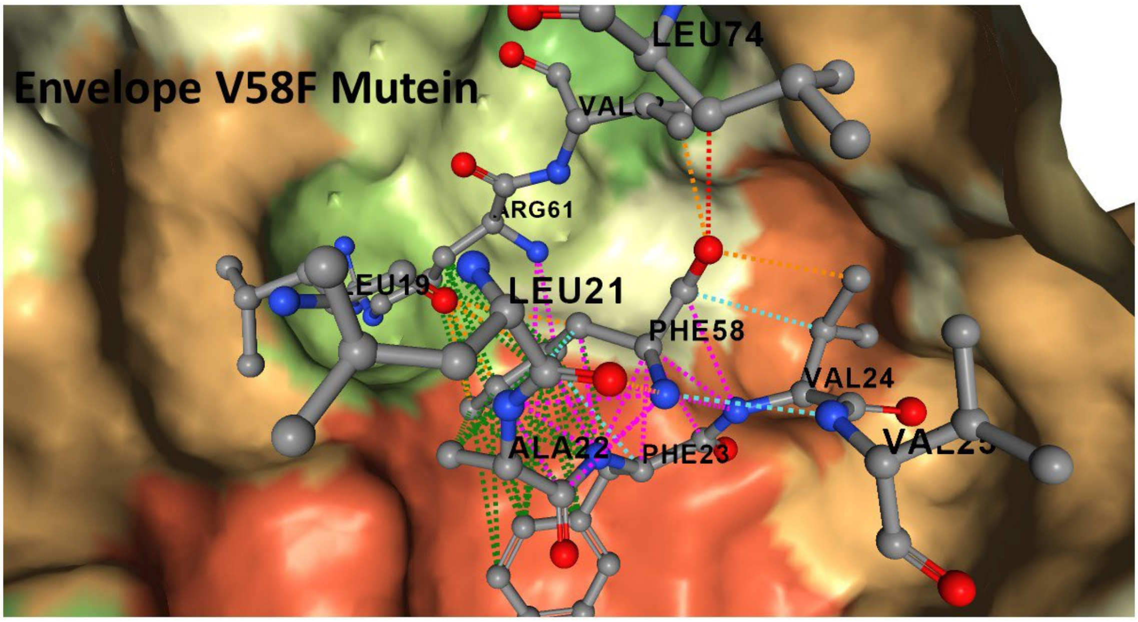
Envelope V58F Mutein
LEU74
ARG61
LEU21
PHE58
VAL24
ALA22
PHE23

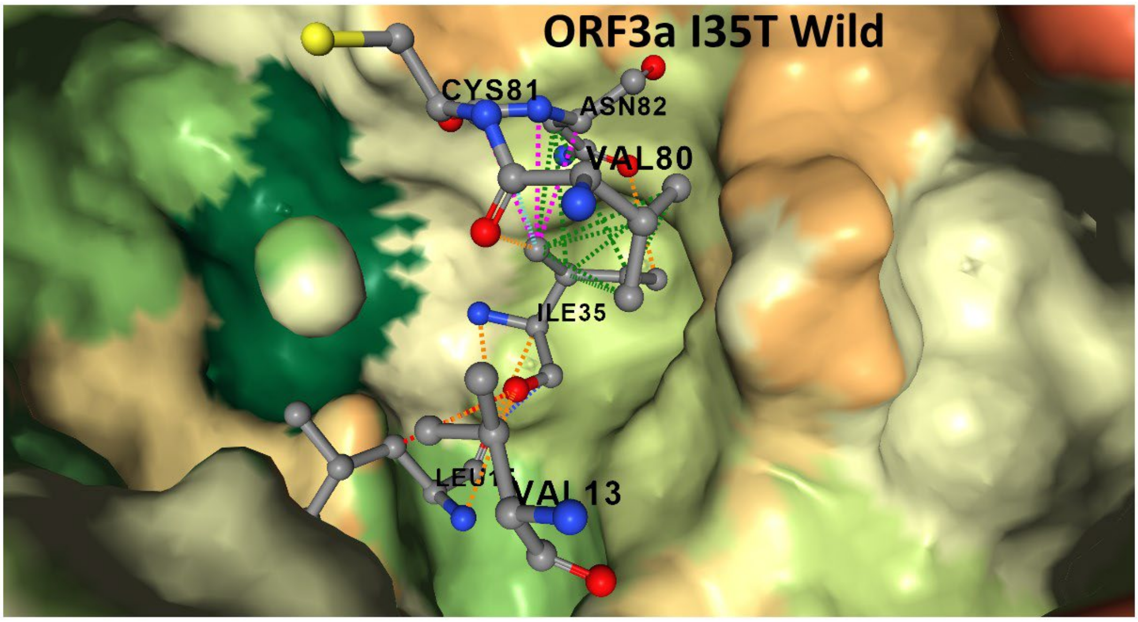
ORF3a I35T Wild
CYS81
ASN82
VAL80
ILE35
VAL13

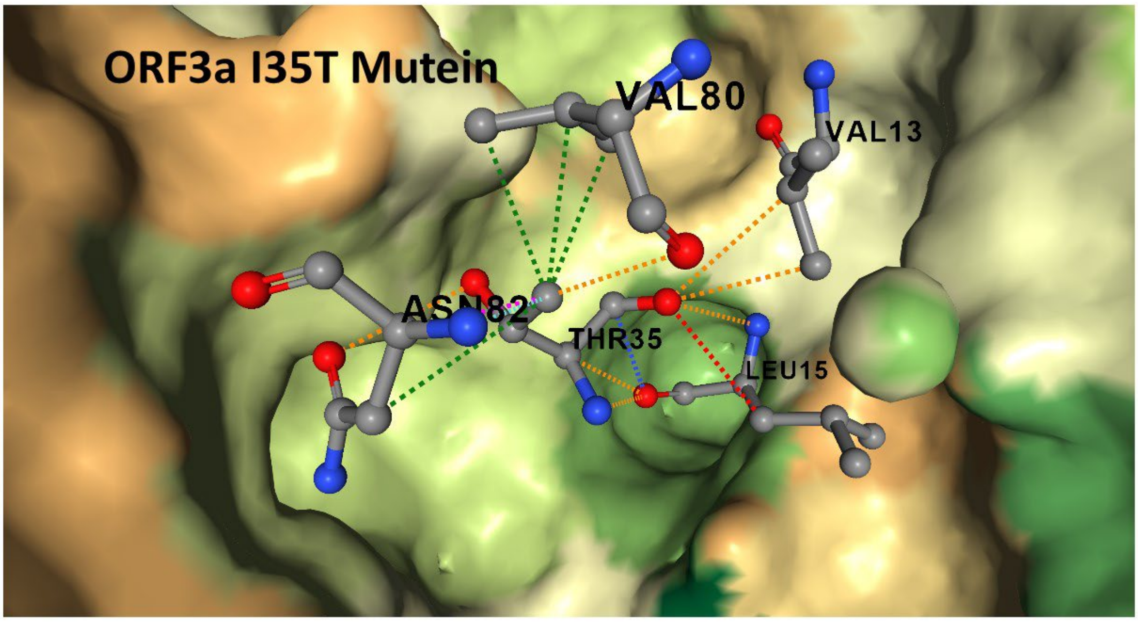
ORF3a I35T Mutein
VAL80
VAL13
ASN82
THR35
LEU15

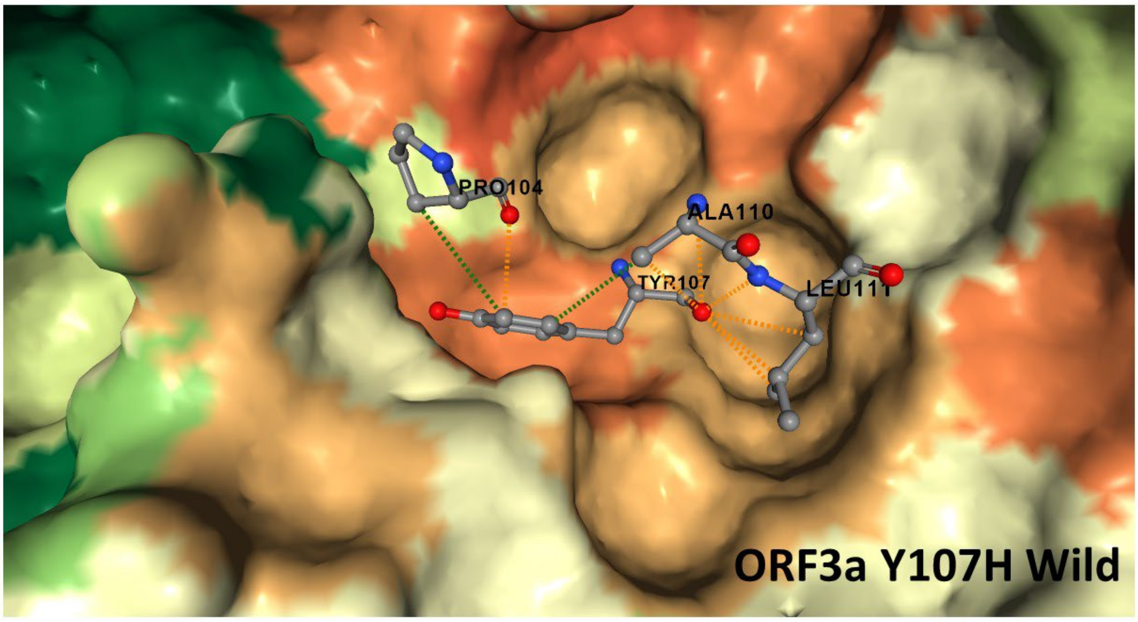
PRO104
ALA110
TYR107
LEU111
ORF3a Y107H Wild

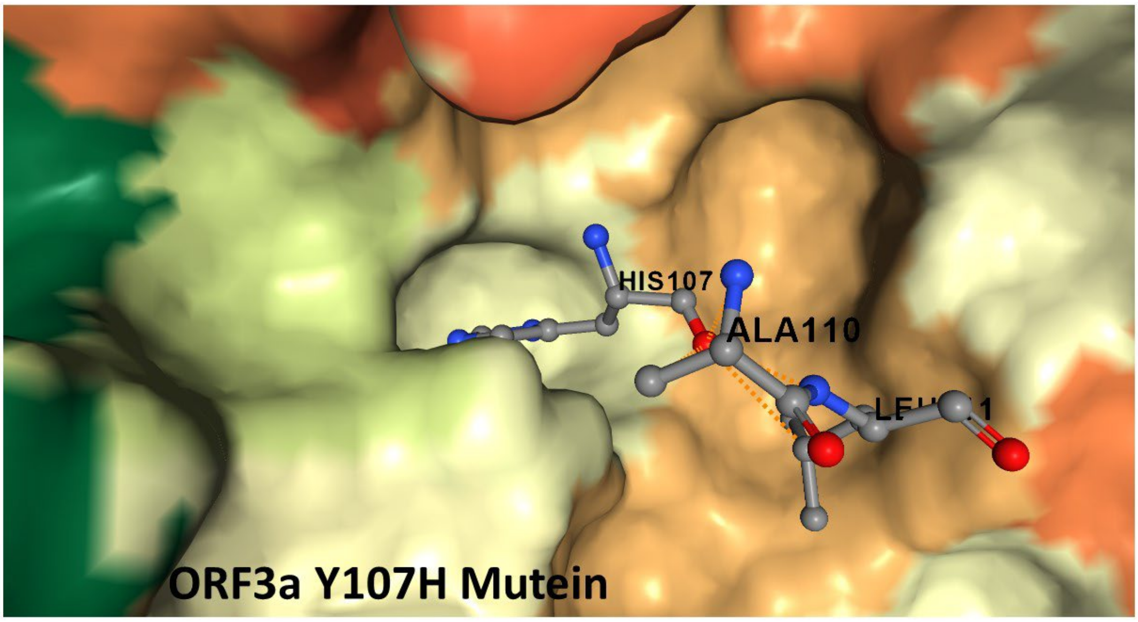
HIS107
ALA110
ORF3a Y107H Mutein

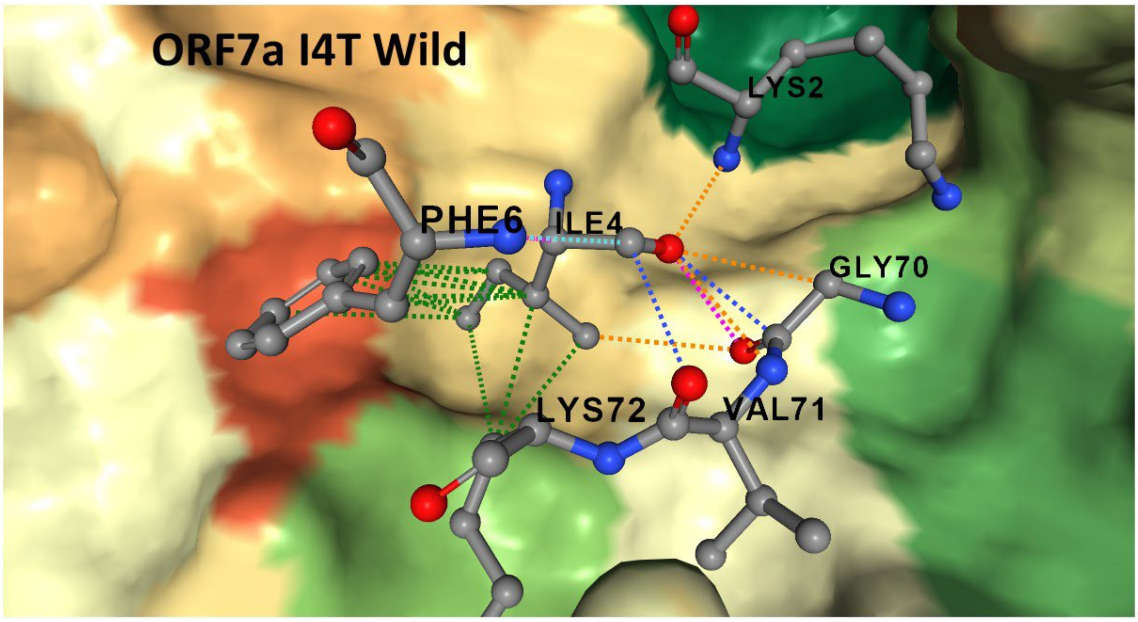
ORF7a I4T Wild
LYS2
PHE6
ILE4
GLY70
LYS72
VAL71

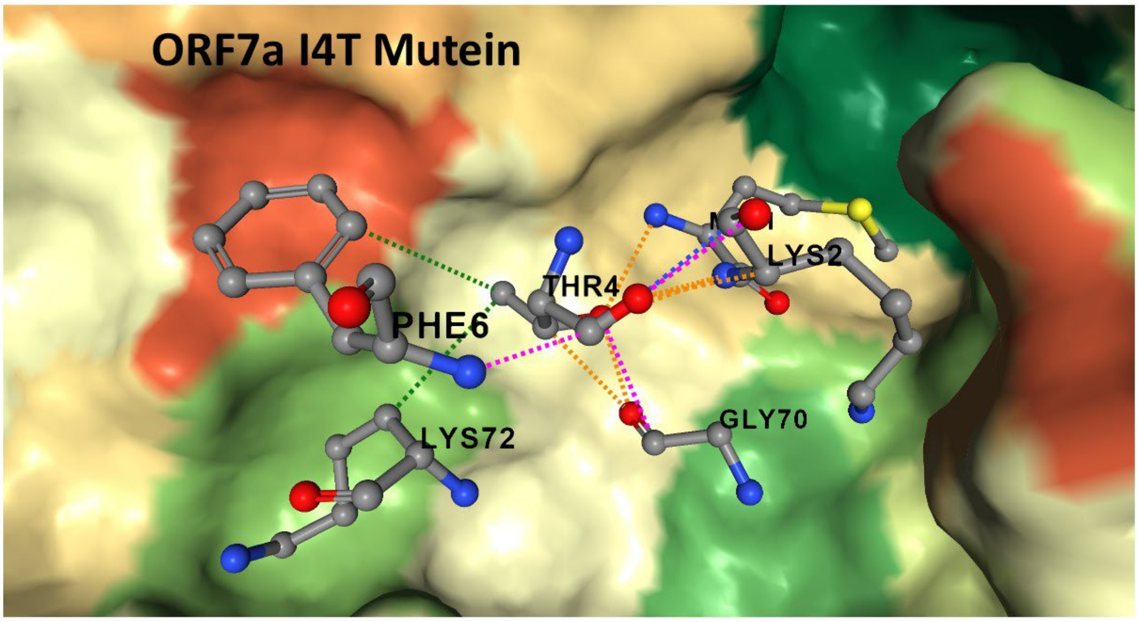
ORF7a I4T Mutein
PHE6
THR4
LYS2
LYS72
GLY70

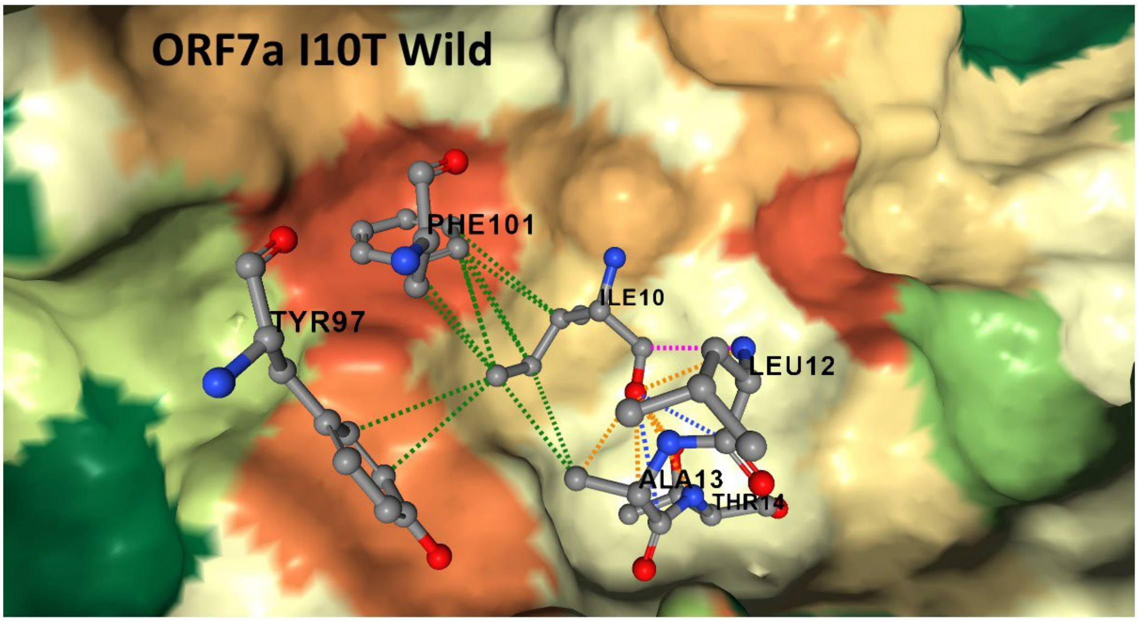
ORF7a I10T Wild
PHE101
TYR97
ILE10
LEU12
ALA13
THR14

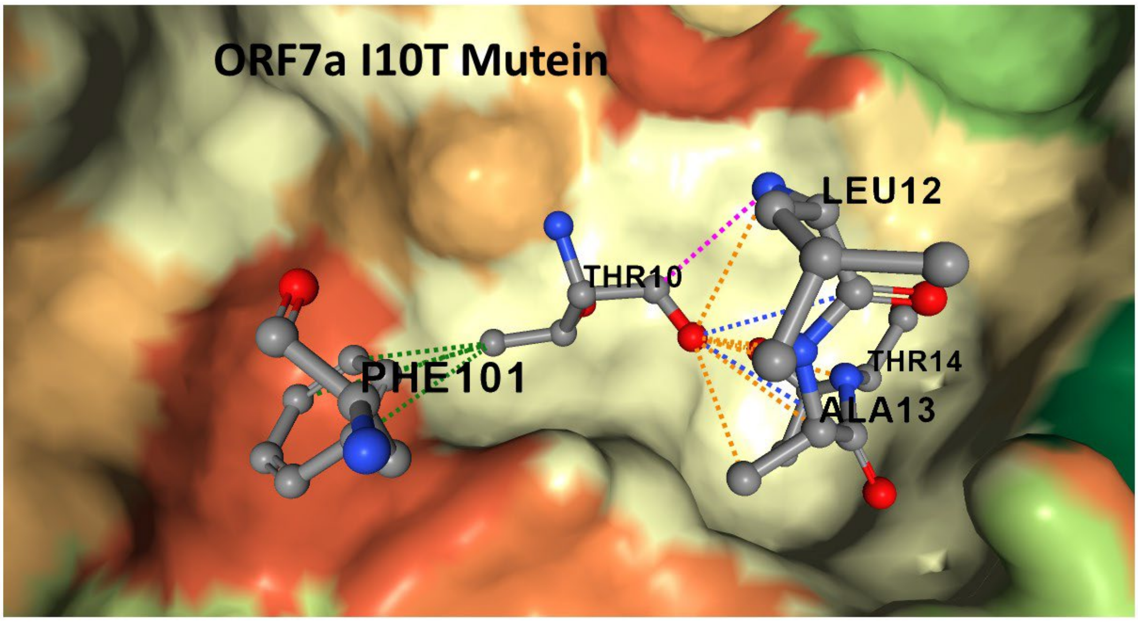
ORF7a I10T Mutein
LEU12
THR10
PHE101
THR14
ALA13

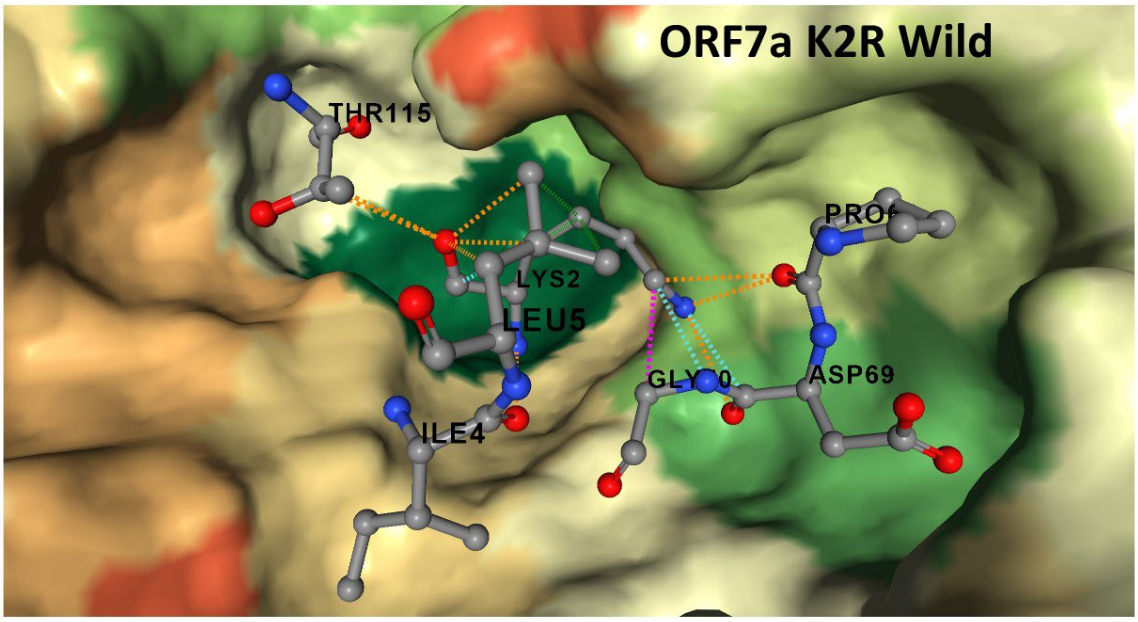
ORF7a K2R Wild
THR115
LYS2
LEU5
ASP69
ILE4

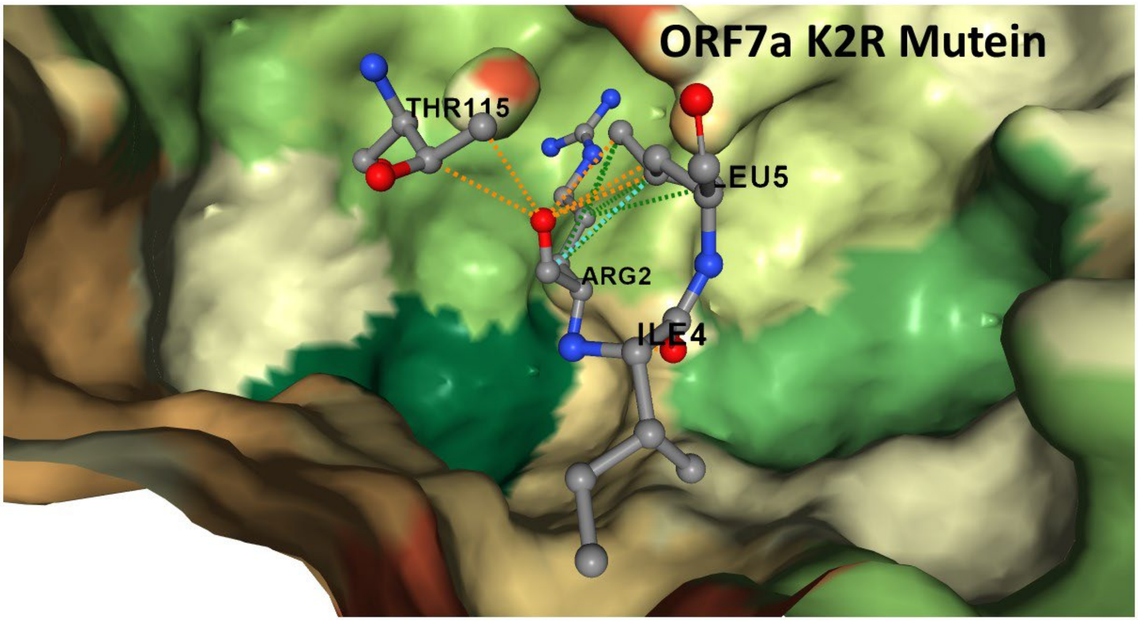
ORF7a K2R Mutein
THR115
LEU5
ARG2
ILE4

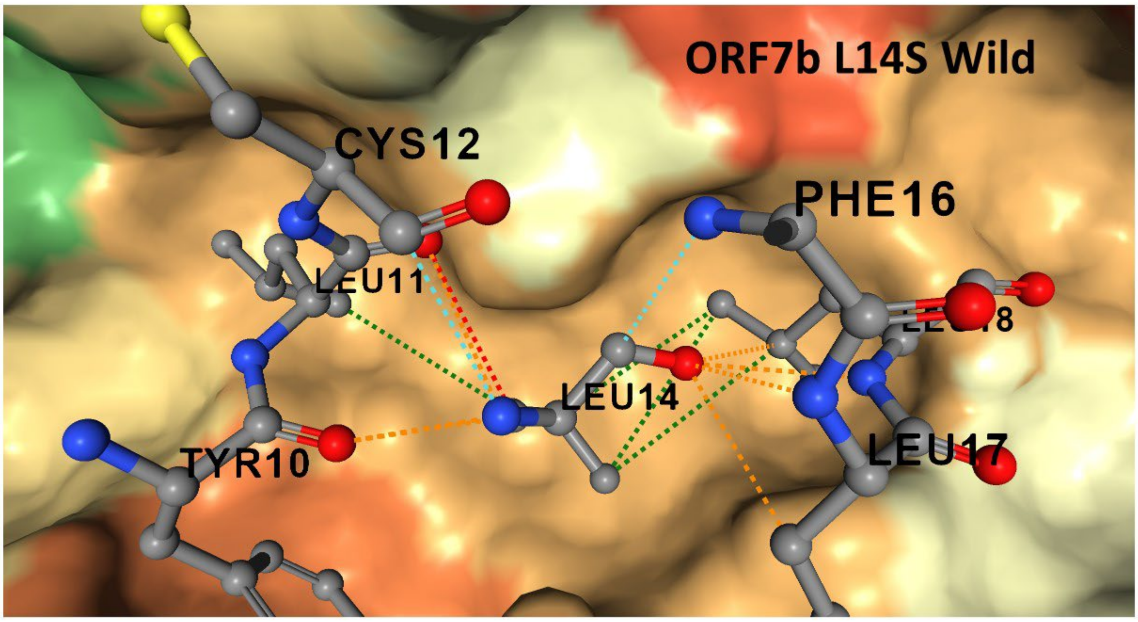
ORF7b L14S Wild
CYS12
PHE16
LEU11
LEU14
TYR10
LEU17

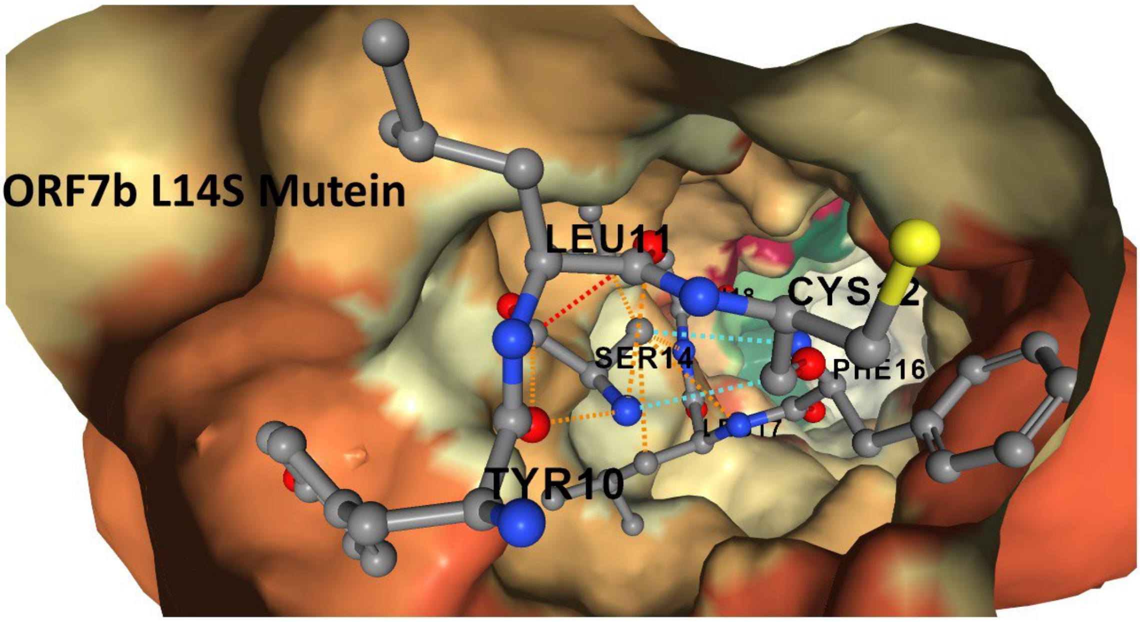
ORF7b L14S Mutein
LEU11
CYS12
SER14
PHE16
TYR10

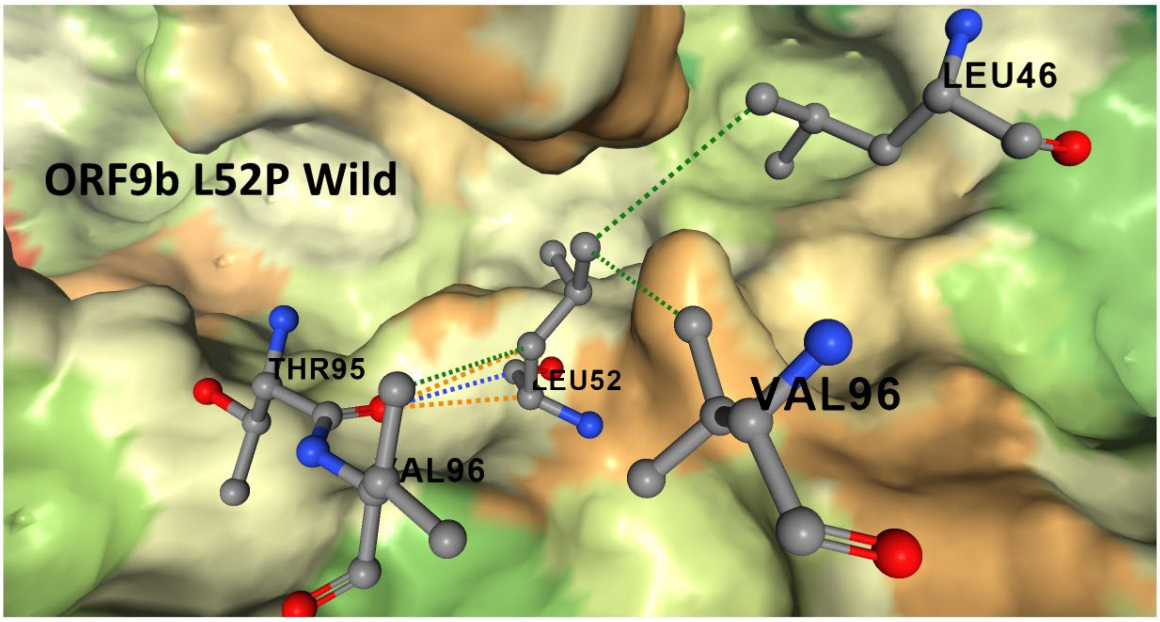
ORF9b L52P Wild
LEU46
THR95
LEU52
VAL96
AL96

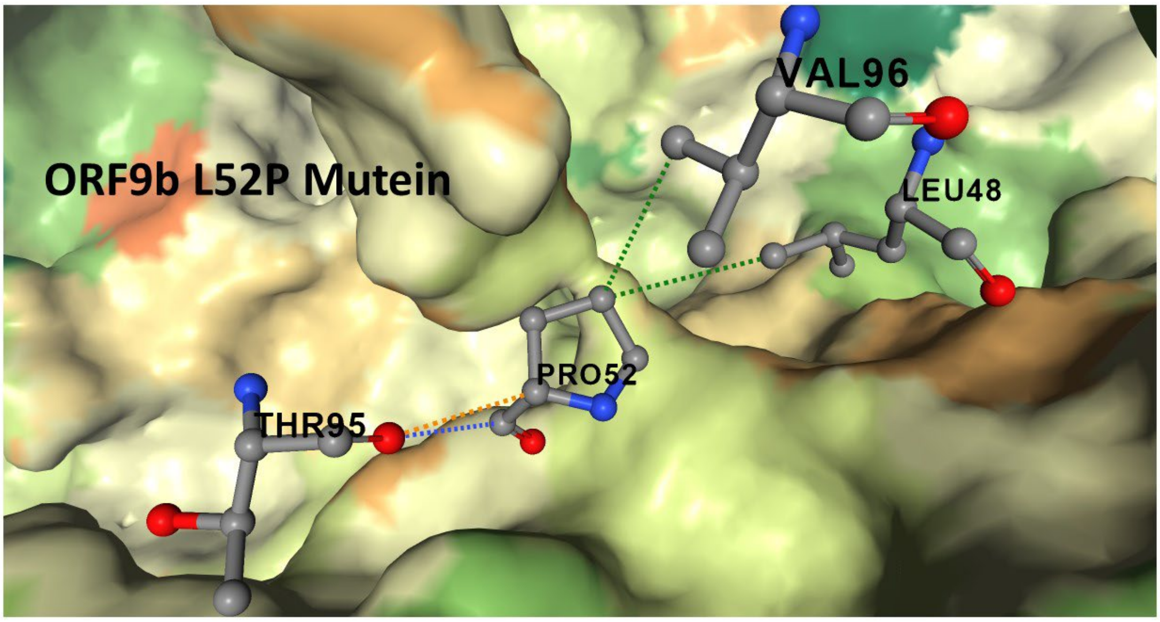
ORF9b L52P Mutein
VAL96
LEU48
PRO52
THR95

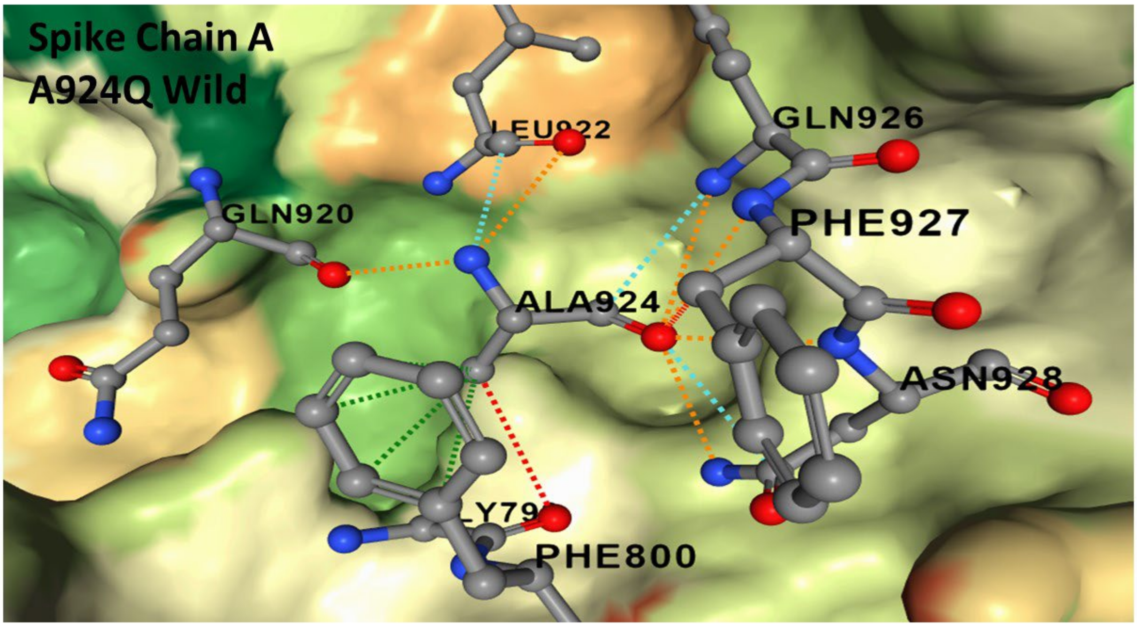
Spike Chain A
A924Q Wild
LEU922
GLN926
GLN920
PHE927
ALA924
ASN928
PHE800

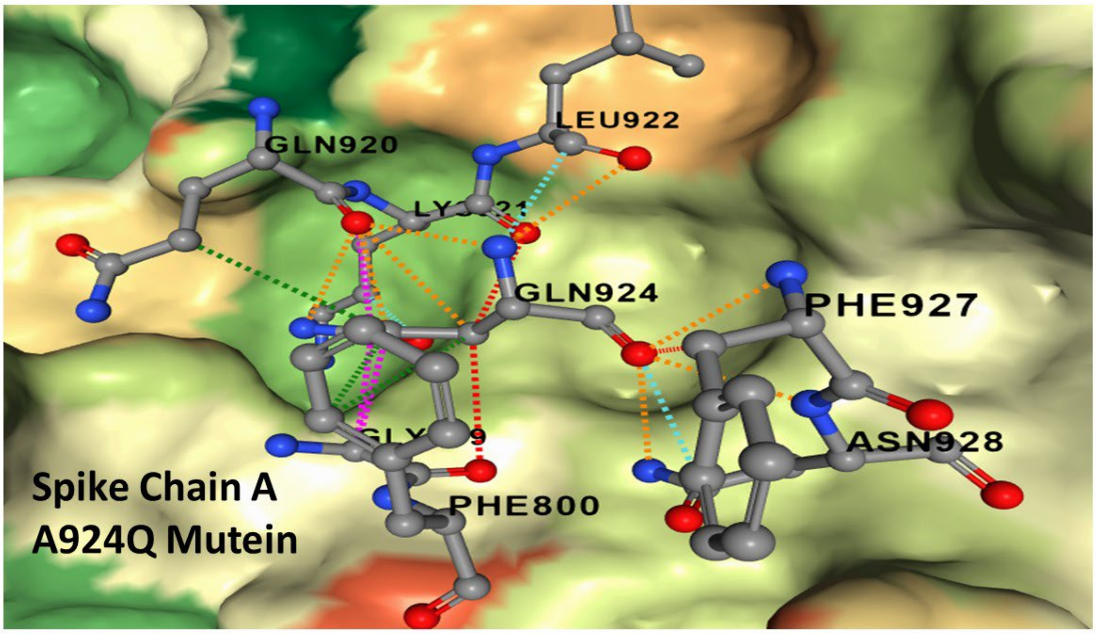
LEU922
GLN920
GLN924
PHE927
ASN928
PHE800
Spike Chain A
A924Q Mutein

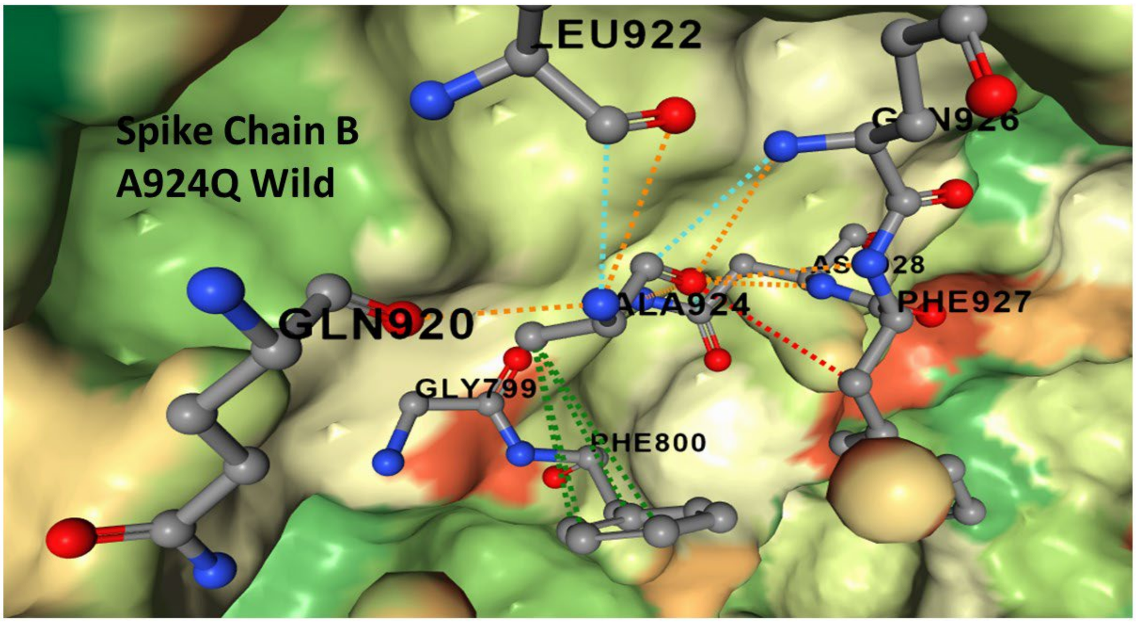
LEU922
Spike Chain B
A924Q Wild
GLN920
ALA924
PHE927
GLY799
PHE800

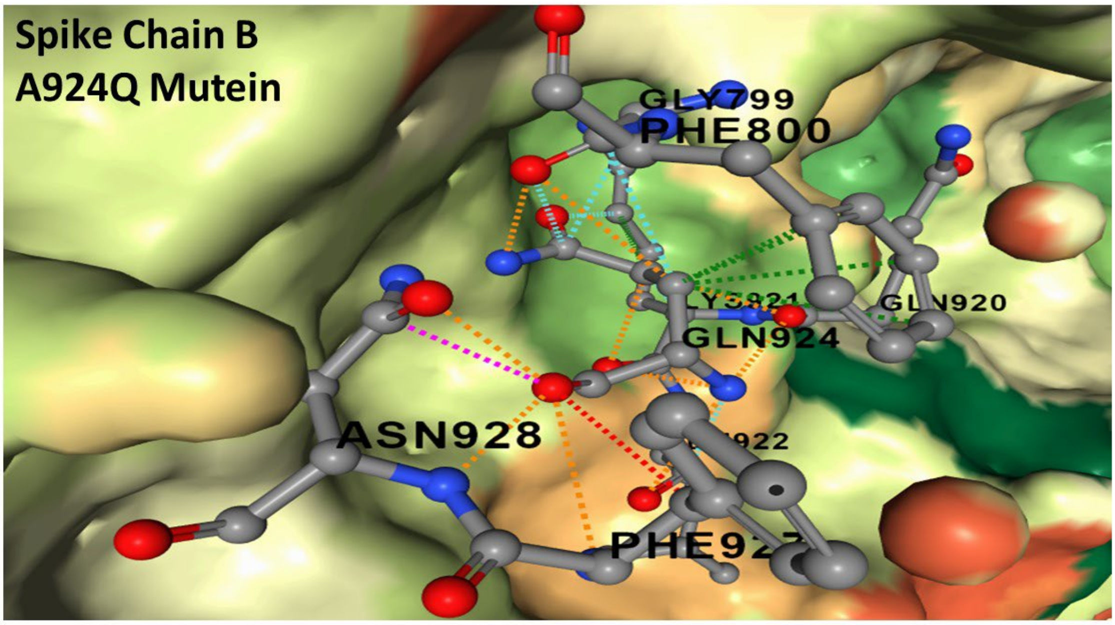
Spike Chain B
A924Q Mutein
GLY799
PHE800
GLN920
GLN924
ASN928
PHE927

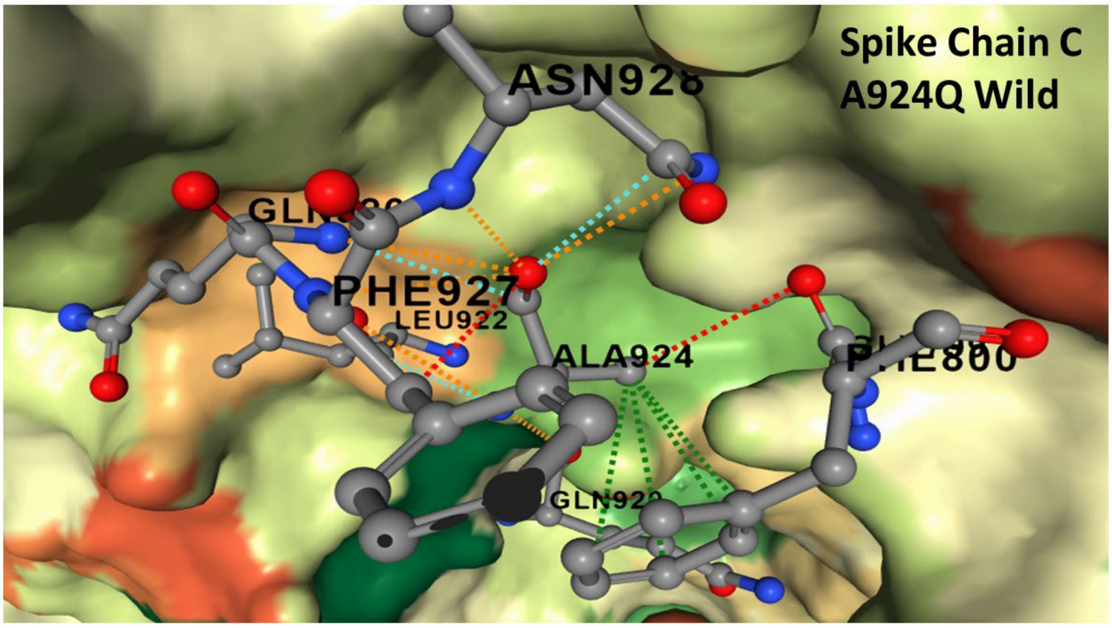
Spike Chain C
A924Q Wild
PHE927
LEU922
ALA924
PHE800

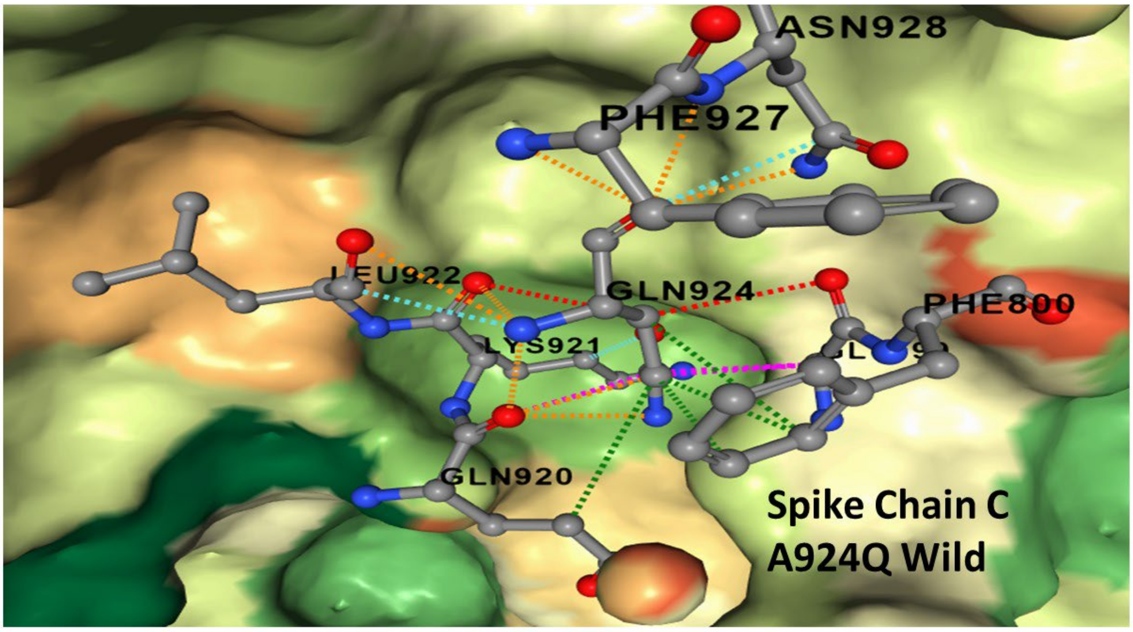
ASN928
PHE927
LEU922
GLN924
PHE800
LYS921
GLN920
Spike Chain C
A924Q Wild

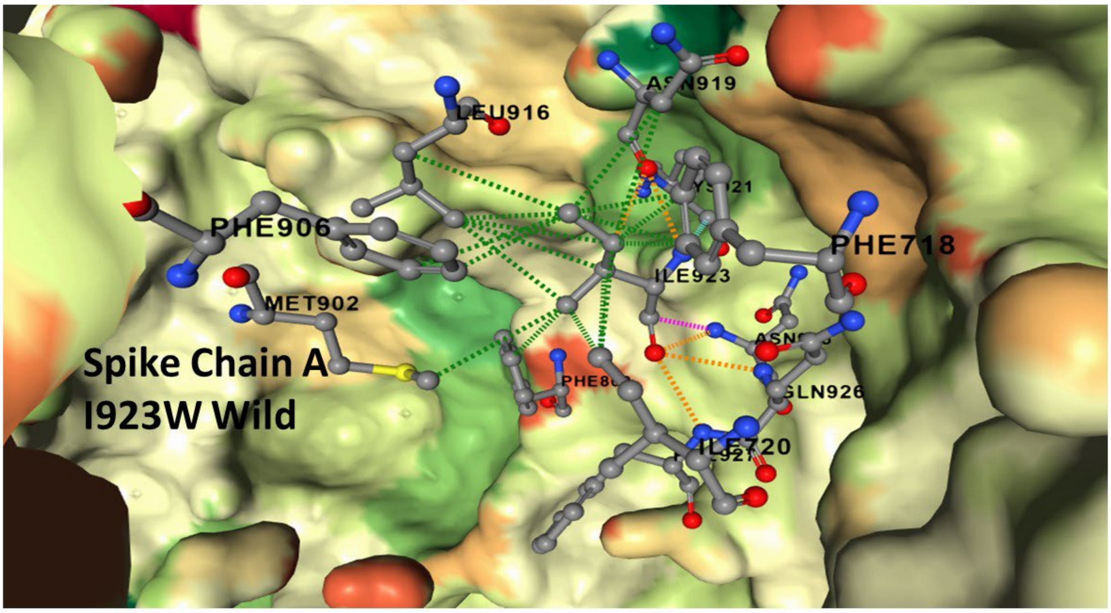
LEU916
PHE906
PHE718
ILE923
MET902
Spike Chain A
I923W Wild
GLN926
ILE720

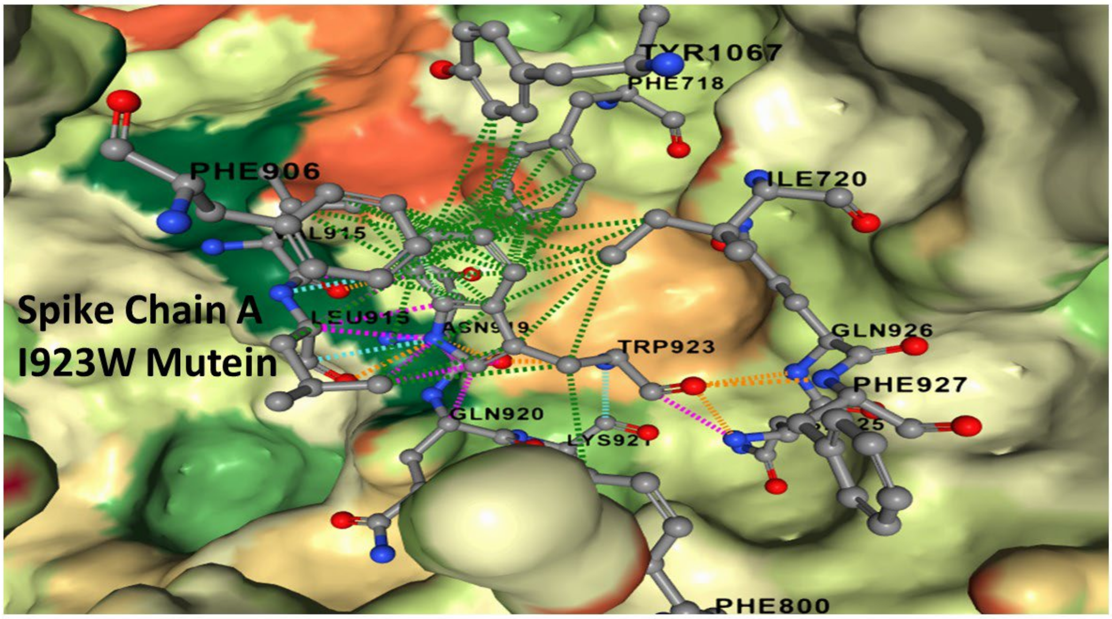
TYR1067
PHE718
PHE906
ILE720
Spike Chain A
I923W Mutein
TRP923
GLN926
PHE927
GLN920
LYS921
PHE800

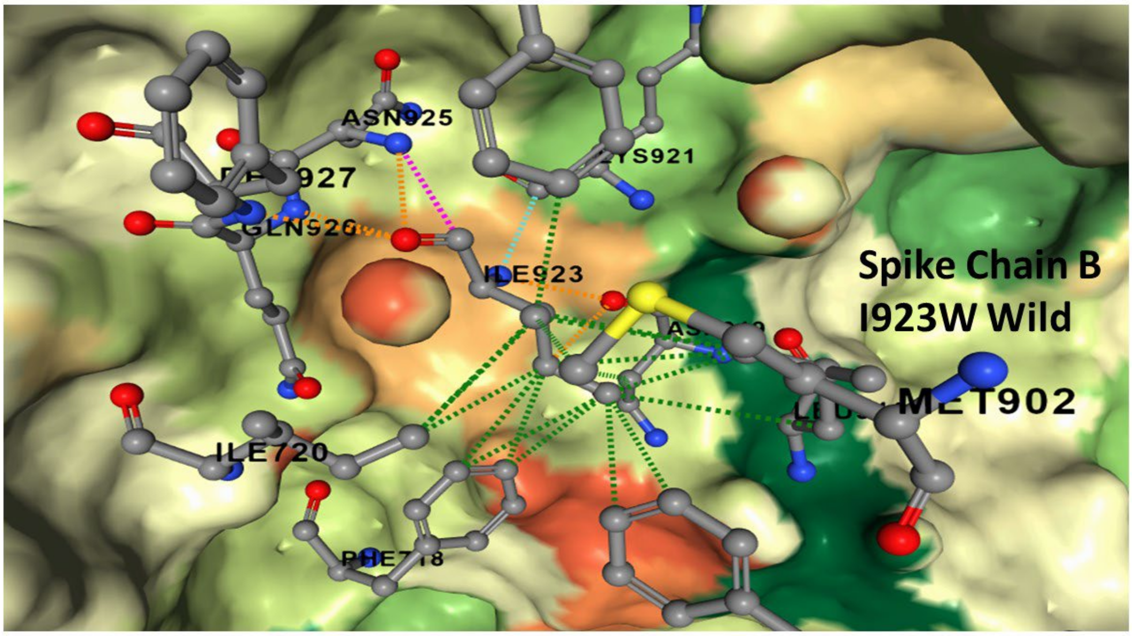
ASN925
ILE923
Spike Chain B
I923W Wild
MET902
ILE720
PHE718

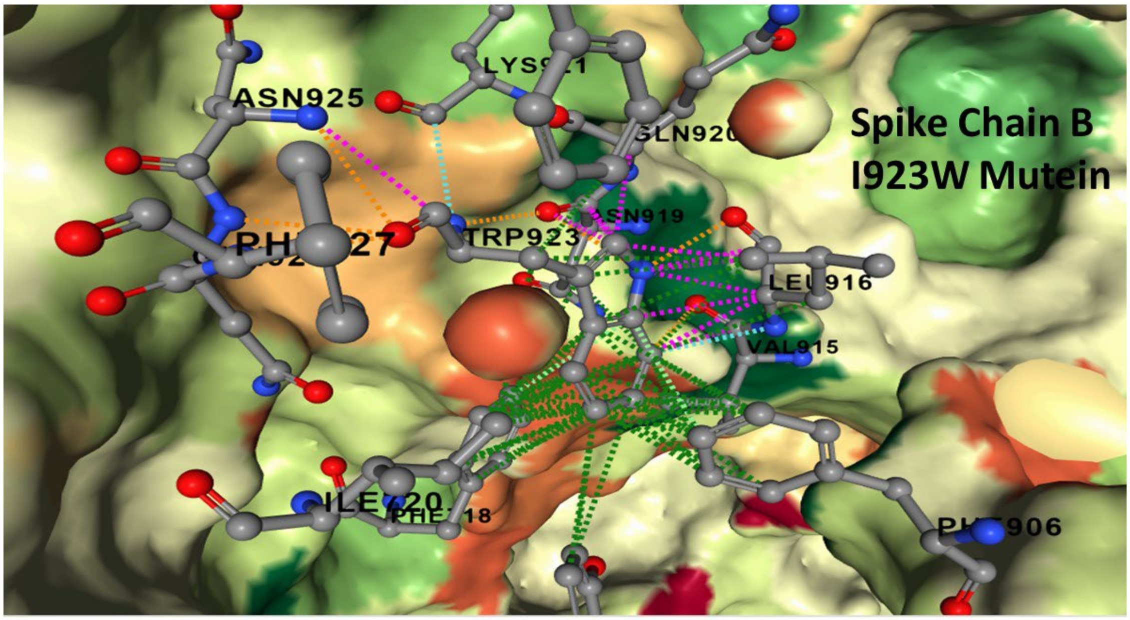
ASN925
GLN920
Spike Chain B
I923W Mutein
ASN919
TRP923
LEU916
VAL915
ILE720
PHE906

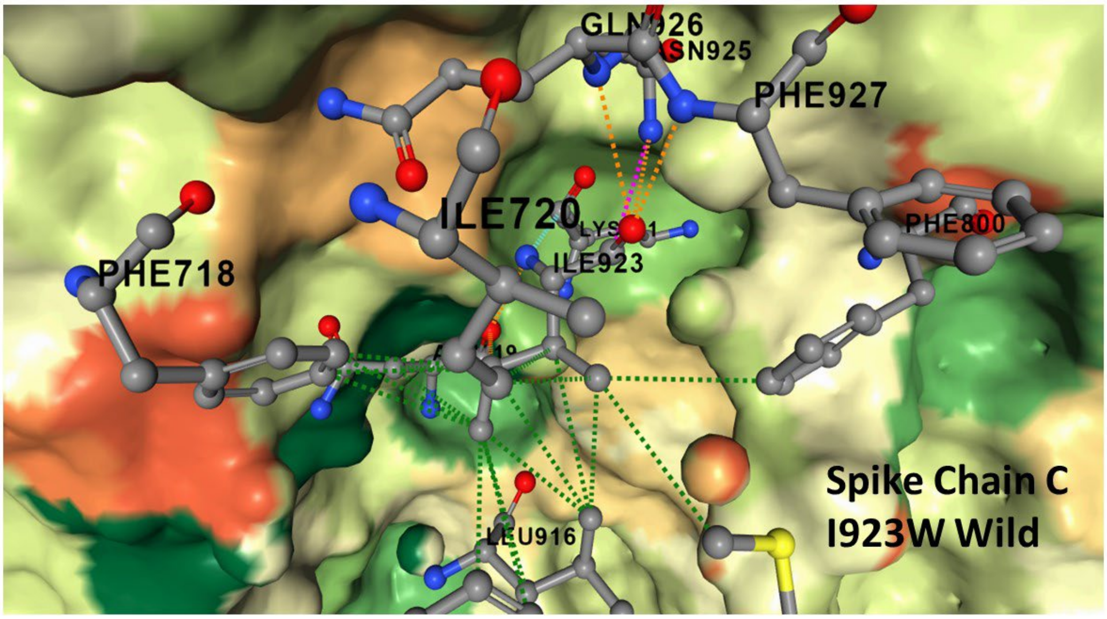
ASN925
PHE927
ILE720
ILE923
PHE800
PHE718
LEU916
Spike Chain C
I923W Wild

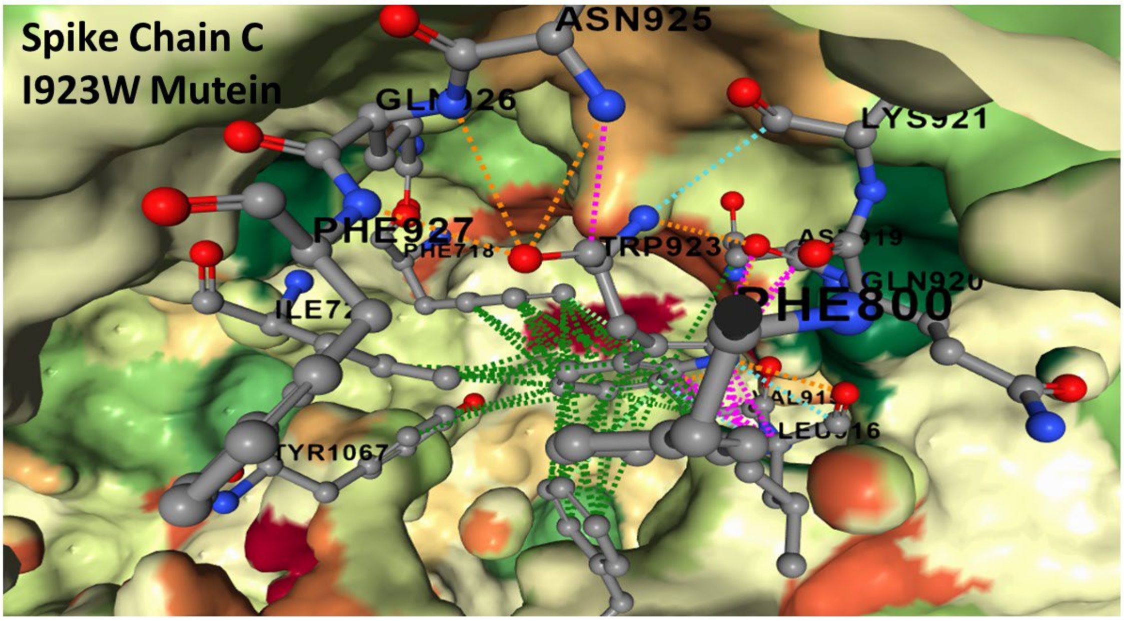
Spike Chain C
I923W Mutein
ASN925
LYS921
PHE927
TRP923
PHE800
GLN920
TYR1067

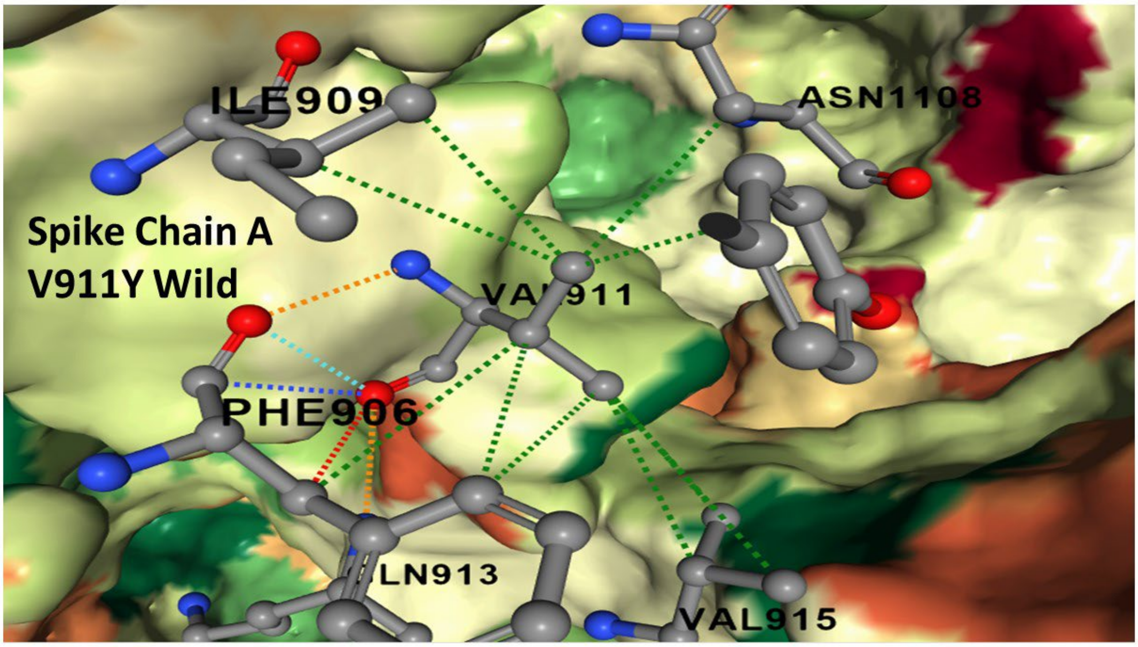
ILE909
ASN1108
Spike Chain A
V911Y Wild
PHE906
VAL915

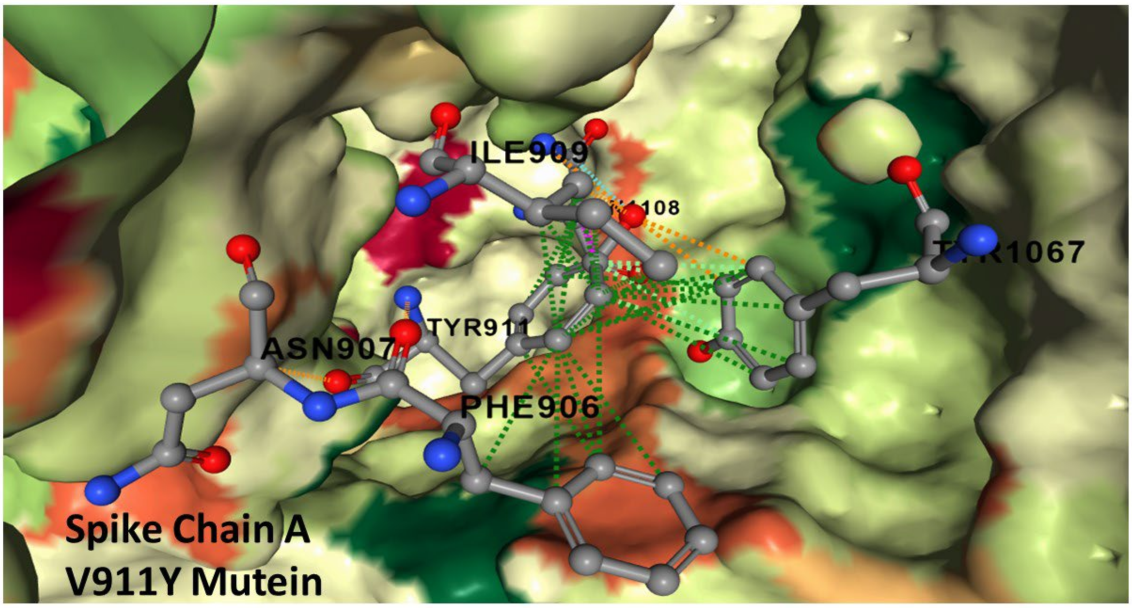
ILE909
TYR911
ASN907
PHE906
Spike Chain A
V911Y Mutein

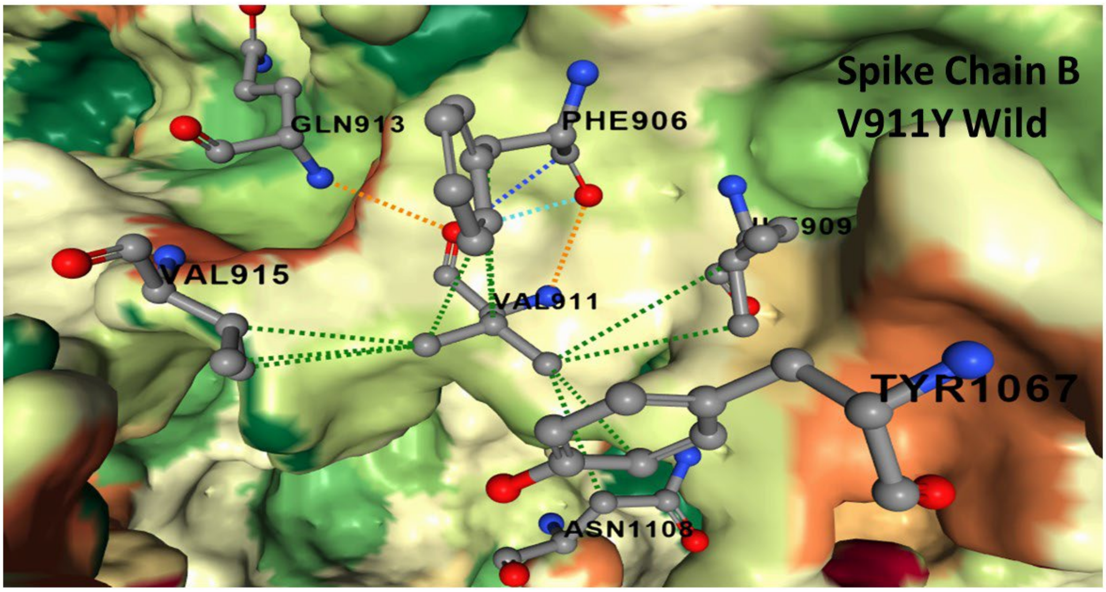
GLN913
PHE906
Spike Chain B
V911Y Wild
VAL915
VAL911
TYR1067
ASN1108

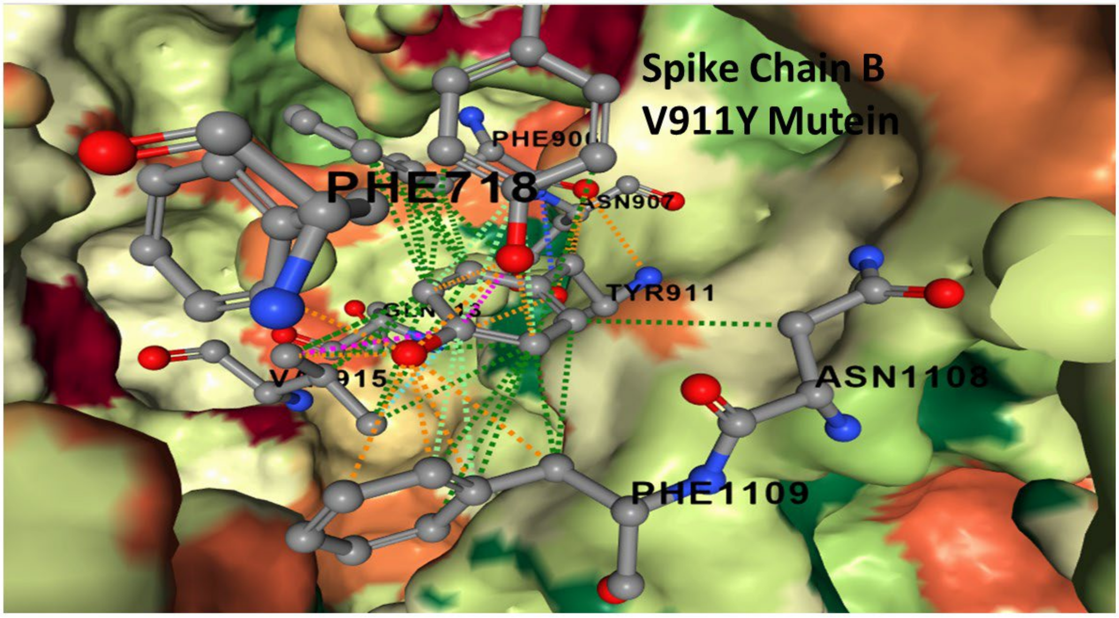
Spike Chain B
V911Y Mutein
PHE718
ASN907
TYR911
ASN1108
PHE1109

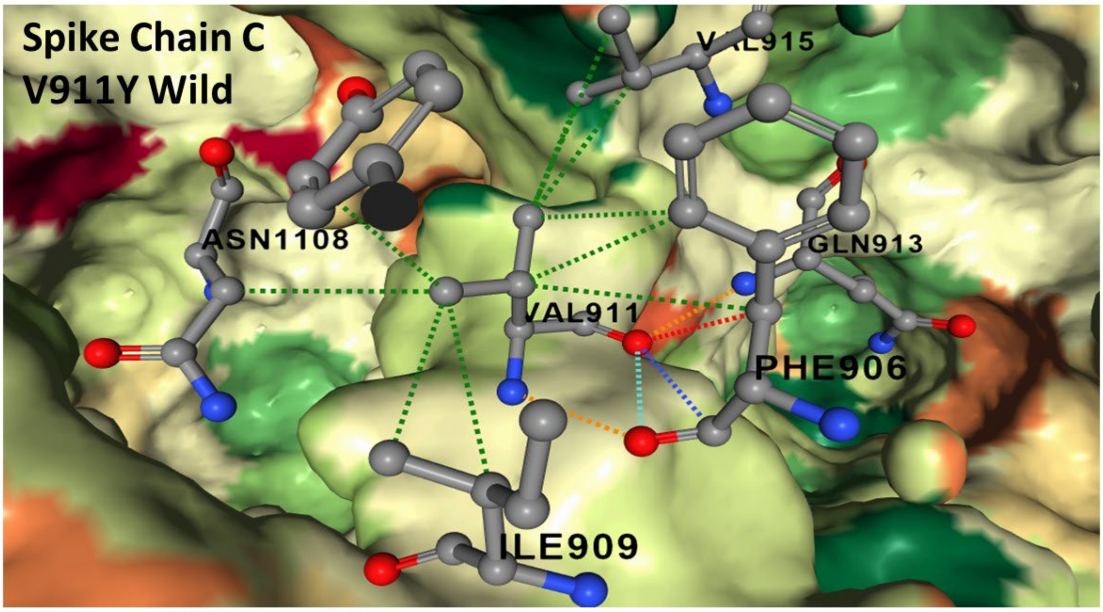
Spike Chain C
V911Y Wild
ASN1108
GLN913
VAL911
PHE906
ILE909

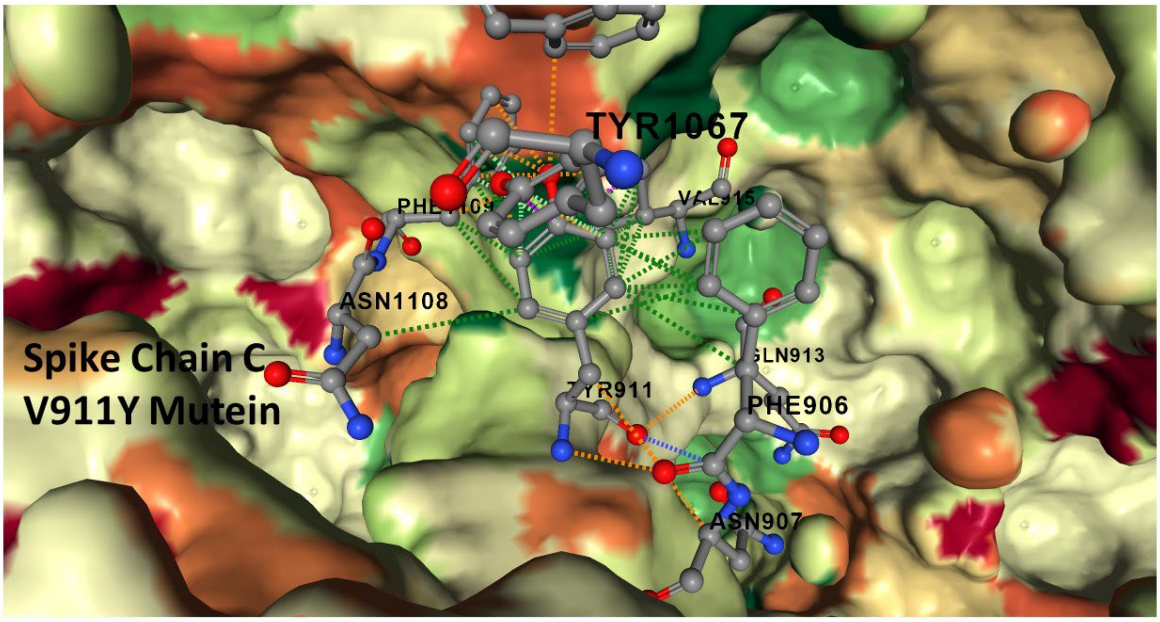
TYR1067
VAL915
ASN1108
Spike Chain C
V911Y Mutein
GLN913
TYR911
PHE906
ASN907

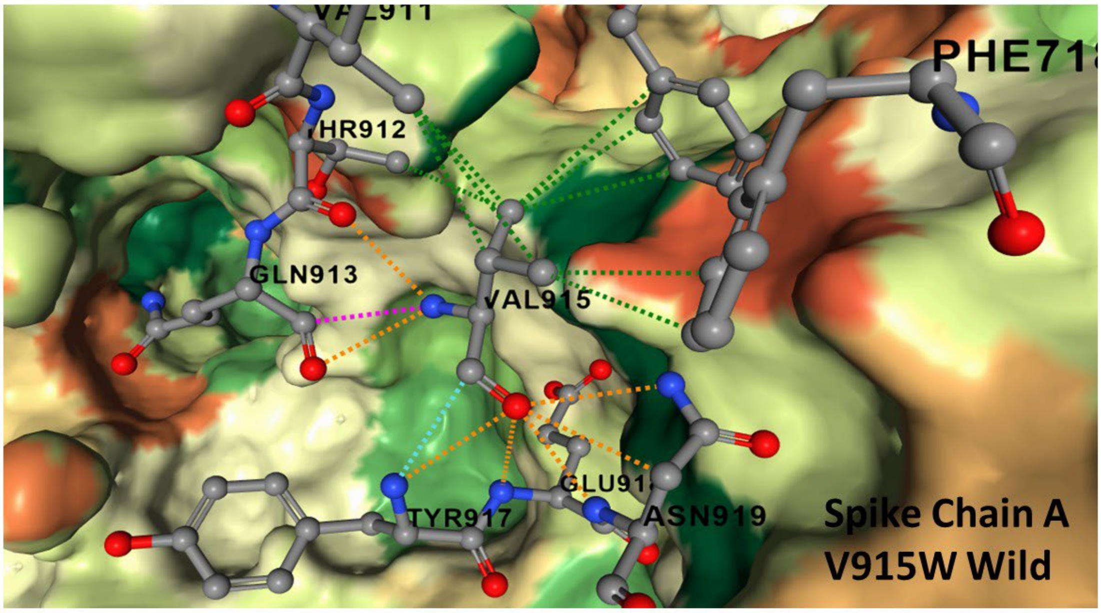
GLN913
VAL915
TYR917
ASN919
Spike Chain A
V915W Wild

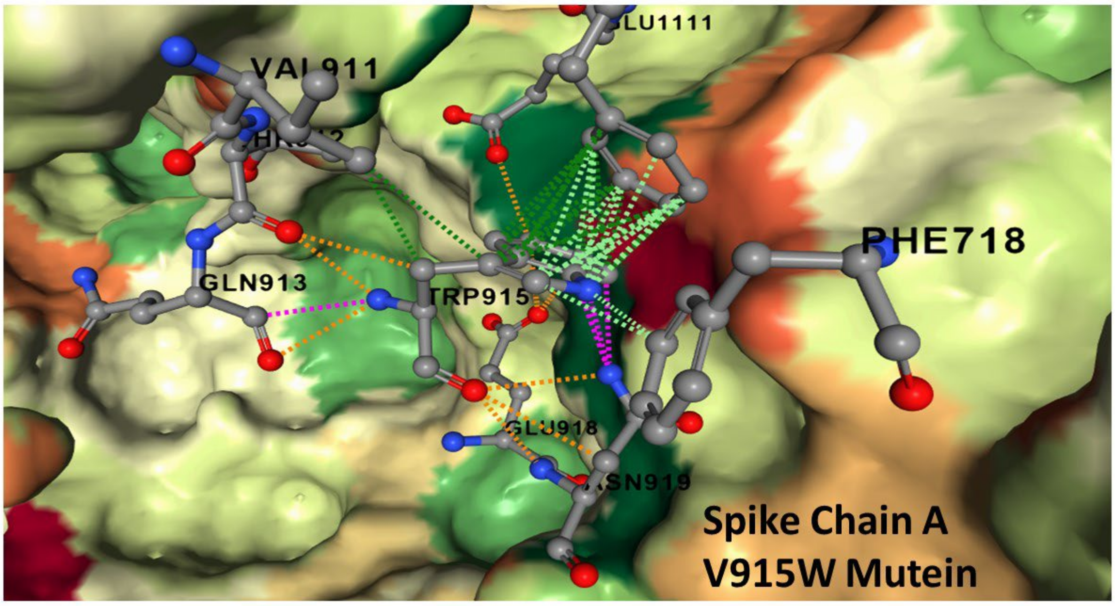
VAL911
GLN913
TRP915
PHE718
GLU918
Spike Chain A
V915W Mutein

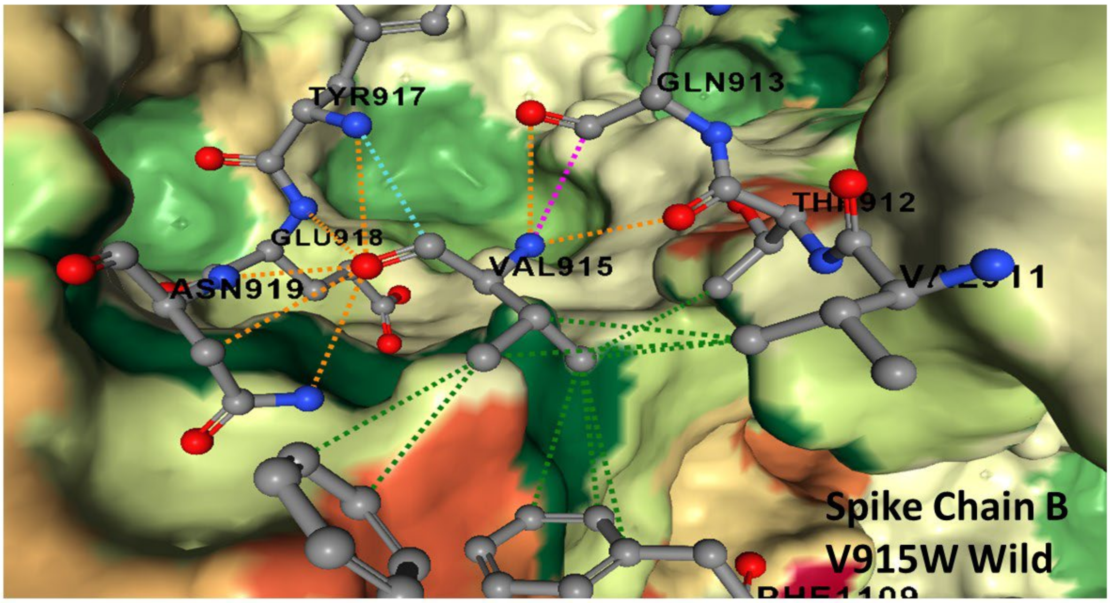
TYR917
GLN913
GLU918
ASN919
VAL915
Spike Chain B
V915W Wild

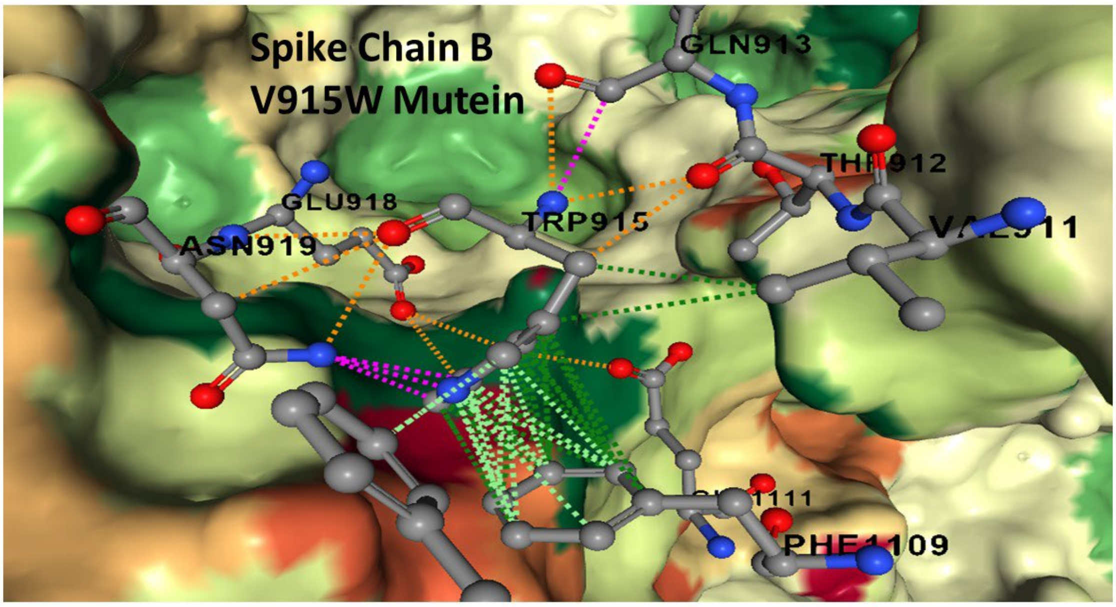
Spike Chain B
V915W Mutein
GLN913
GLU918
TRP915
ASN919
PHE1109

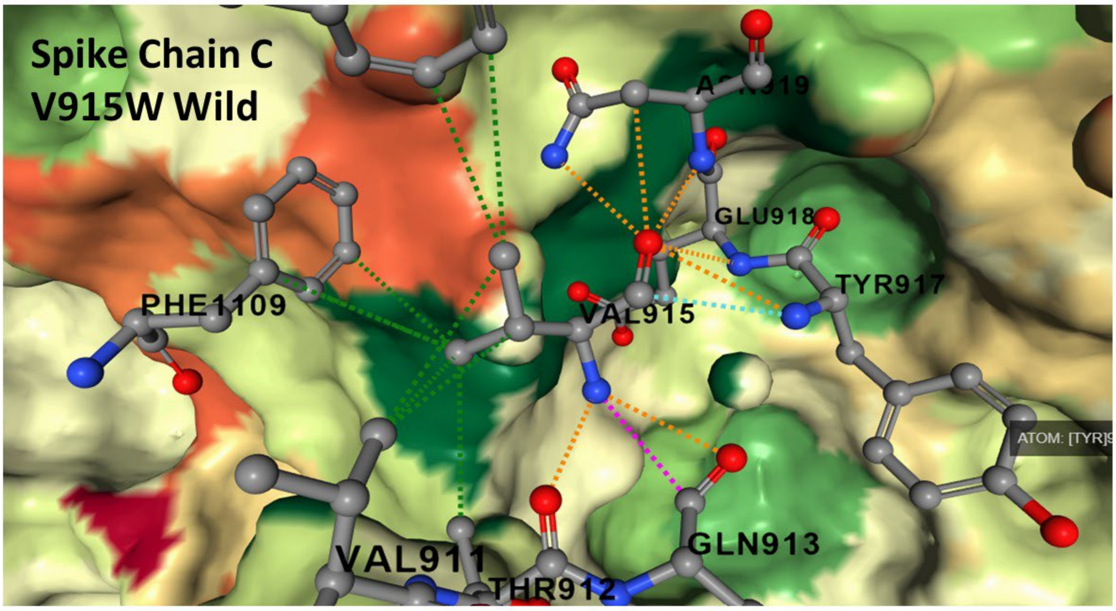
Spike Chain C
V915W Wild
GLU918
TYR917
PHE1109
VAL915
GLN913
VAL911
THR912

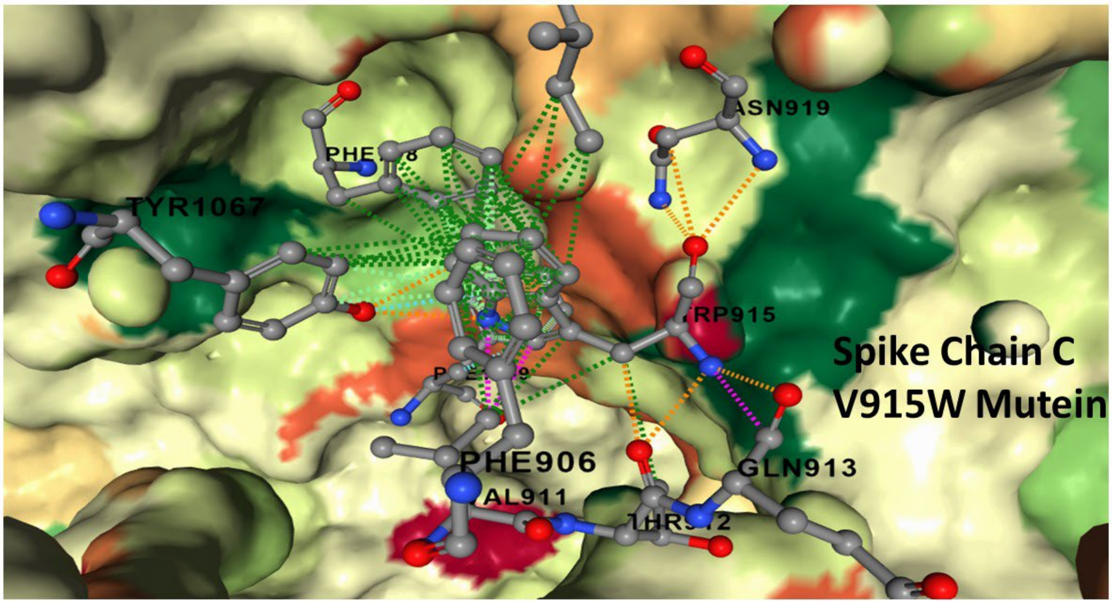
ASN919
TYR1067
RP915
Spike Chain C
V915W Mutein
PHE906
AL911
GLN913
