# Supplementary material for: Variant-specific deleterious mutations in the SARS-CoV-2 genome reveal immune responses and potentials for prophylactic vaccine development
Source: Front Pharmacol. 2023 Feb 7;14:1090717. doi: 10.3389/fphar.2023.1090717 (PMC9941545; doi:10.3389/fphar.2023.1090717)
Supplement: Supplementary file 4 [file Image3.pdf]

Effect of missense mutation on protein-protein interaction for Omicron variant (Spike Protein)

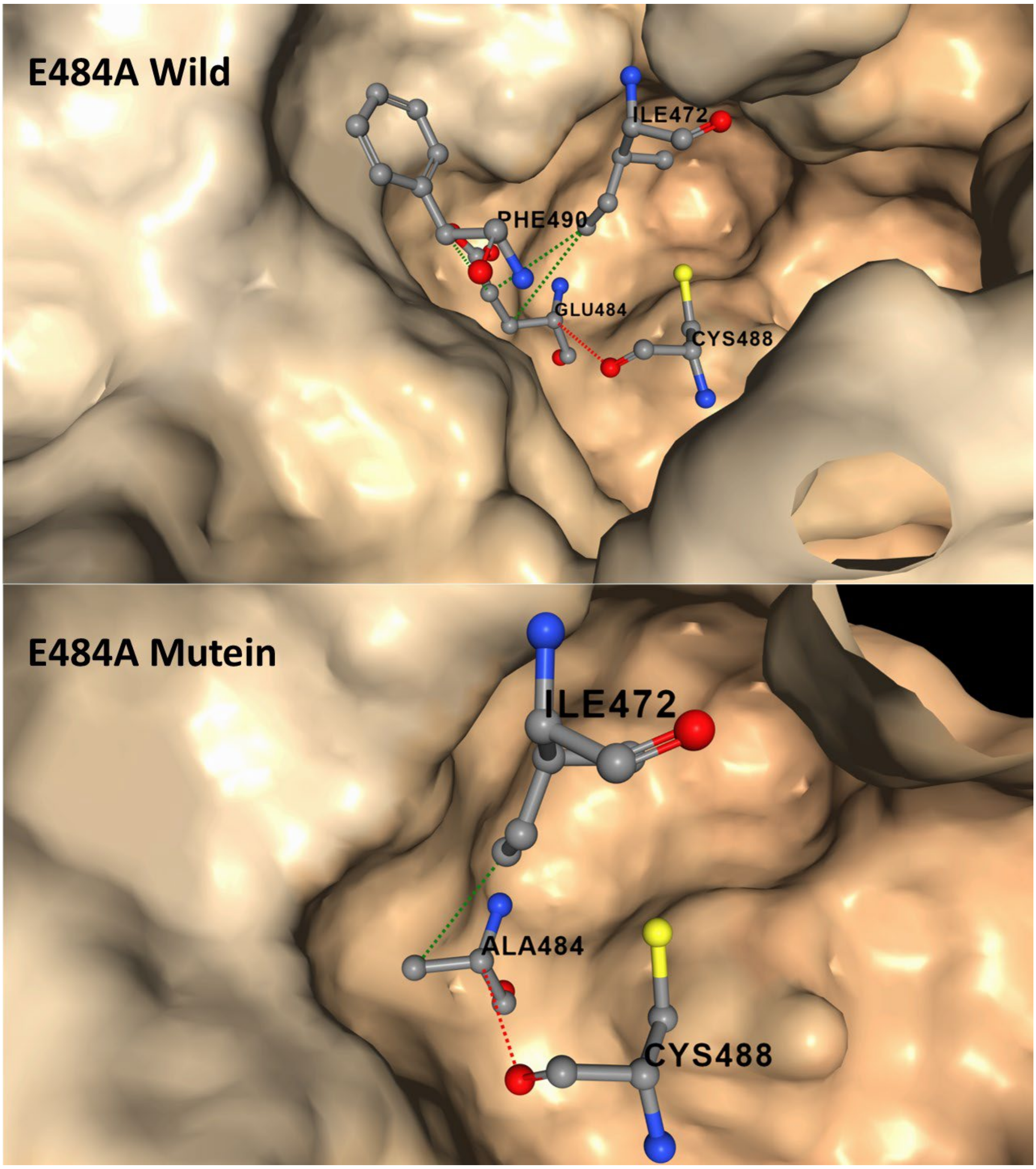
E484A Wild
ILE472
PHE490
GLU484
CYS488
E484A Mutein
ILE472
ALA484
CYS488

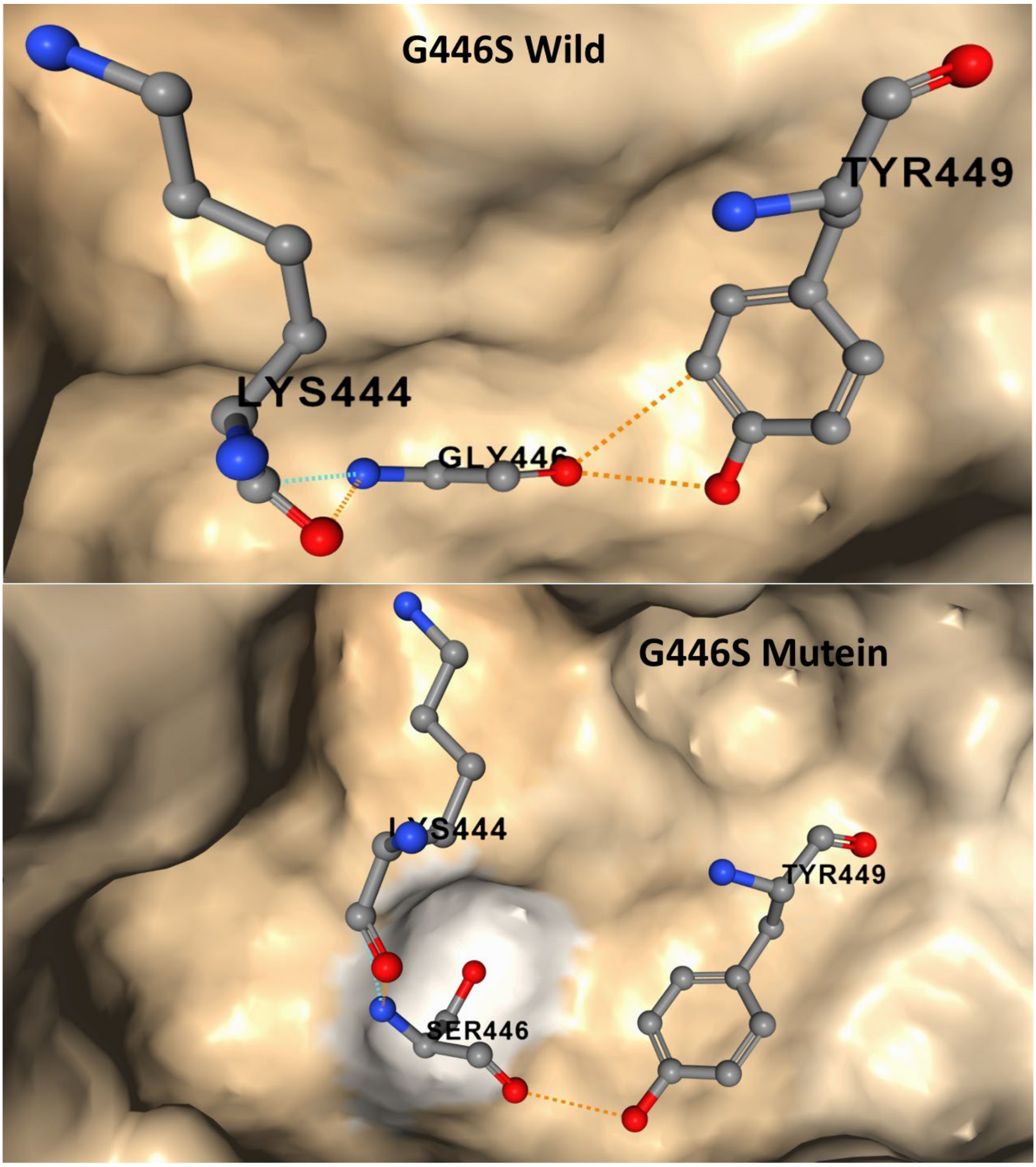

G446S Wild
TYR449
LYS444
GLY446
G446S Mutein
LYS444
TYR449
SER446

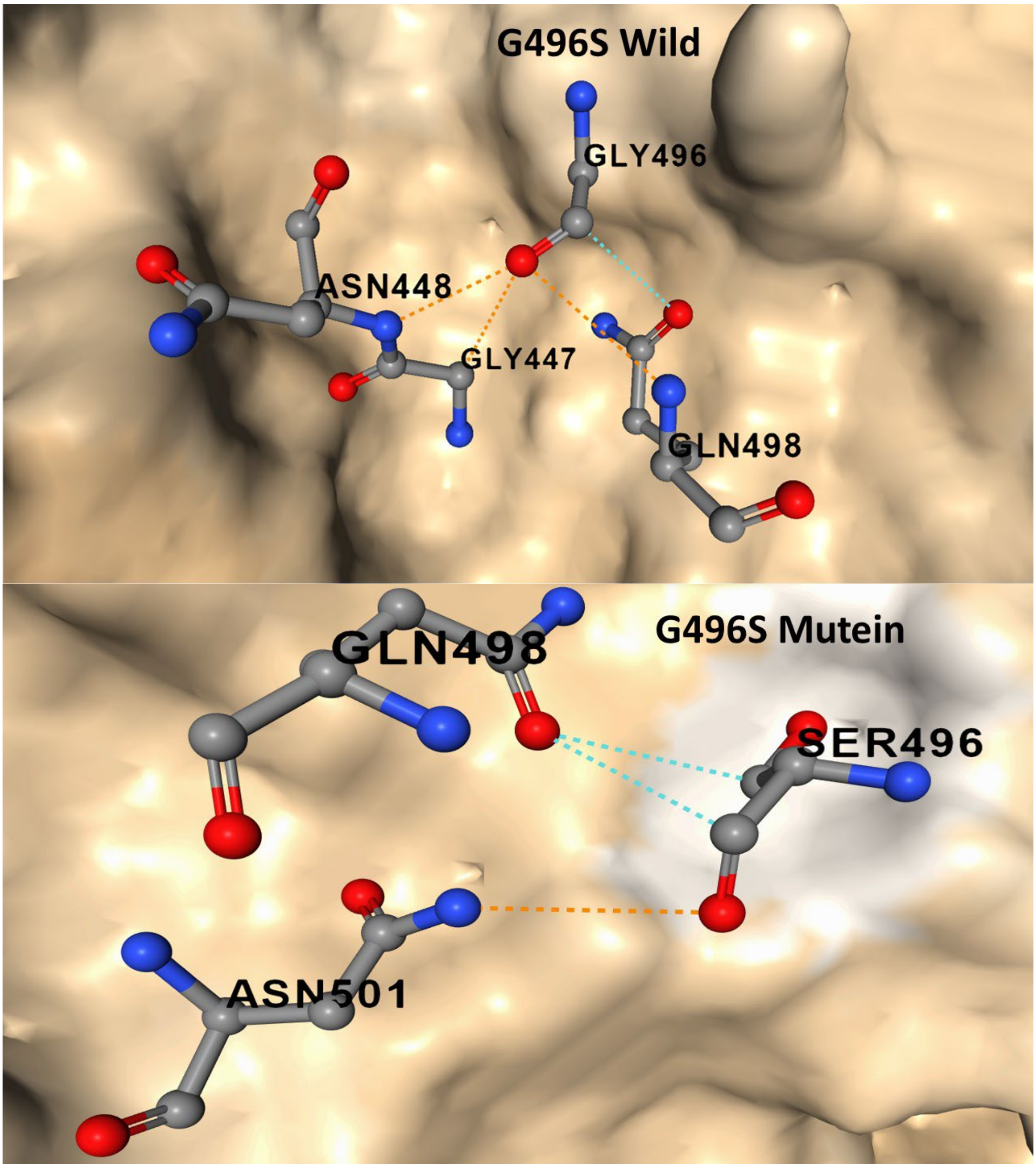
G496S Wild
GLY496
ASN448
GLY447
GLN498
GLN498
G496S Mutein
SER496
ASN501

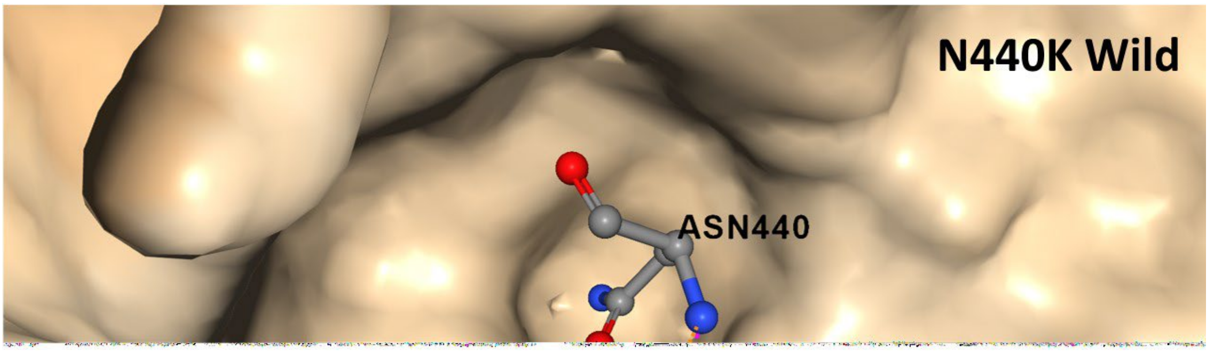
N440K Wild
ASN440

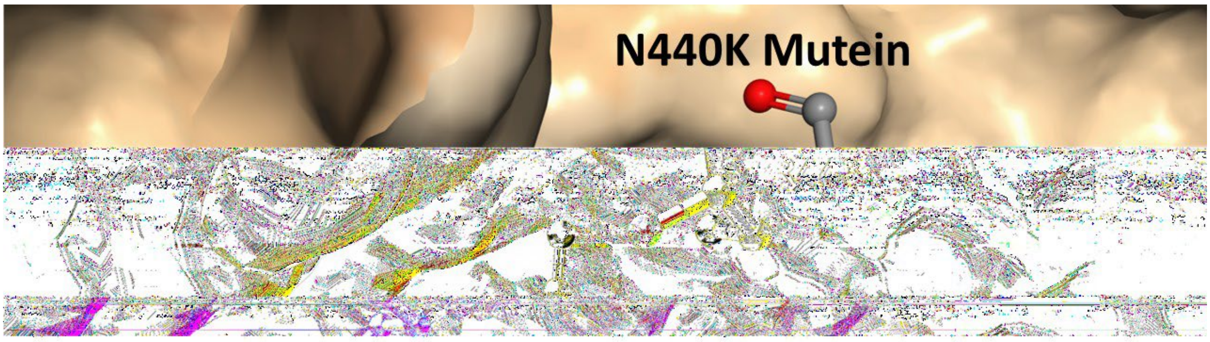
N440K Mutein

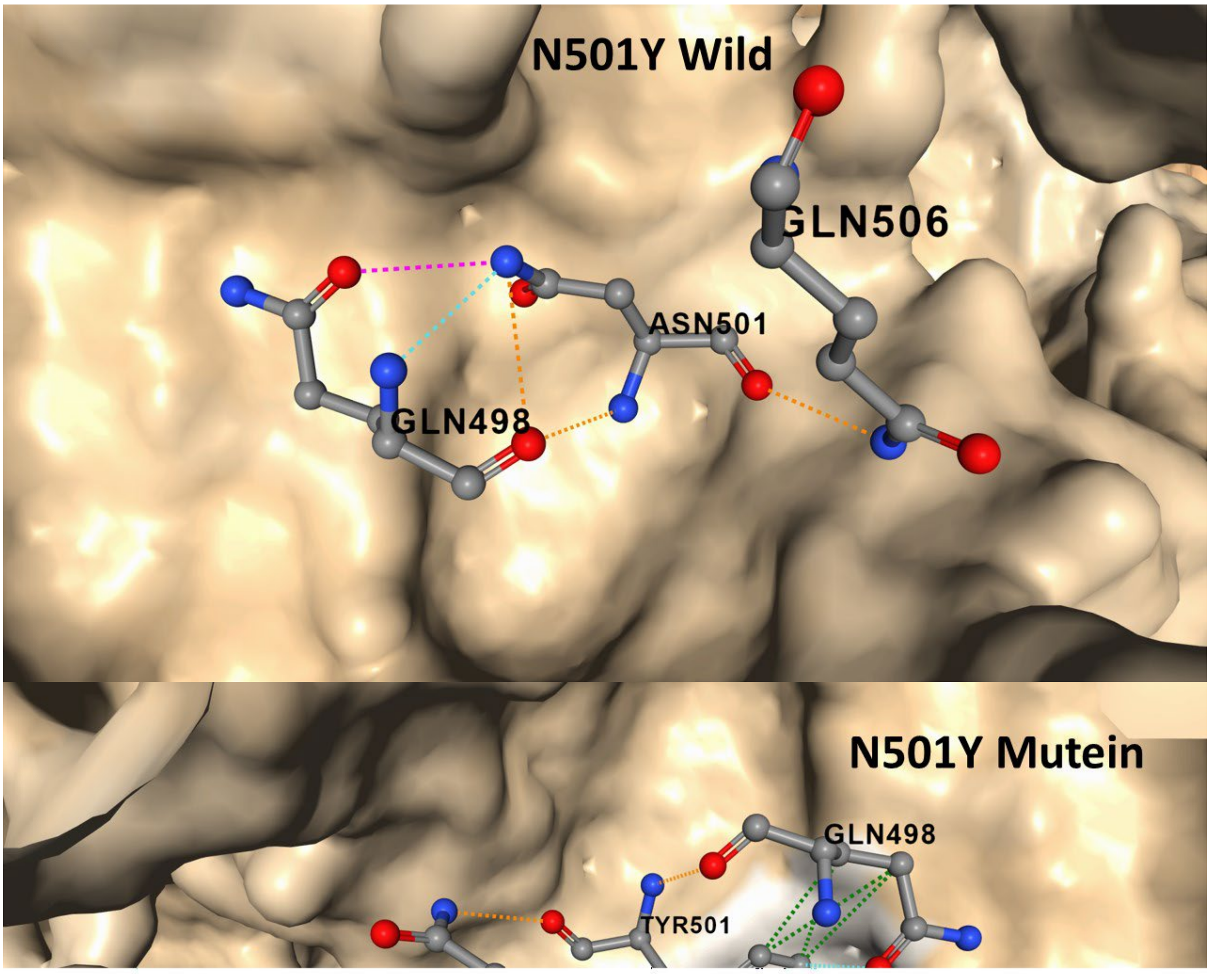
N501Y Wild
GLN506
ASN501
GLN498
N501Y Mutein
GLN498
TYR501

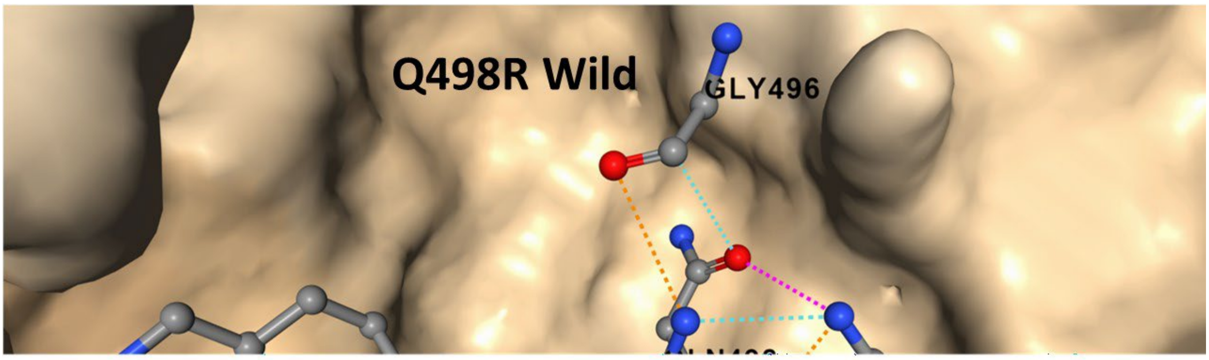
Q498R Wild
GLY496

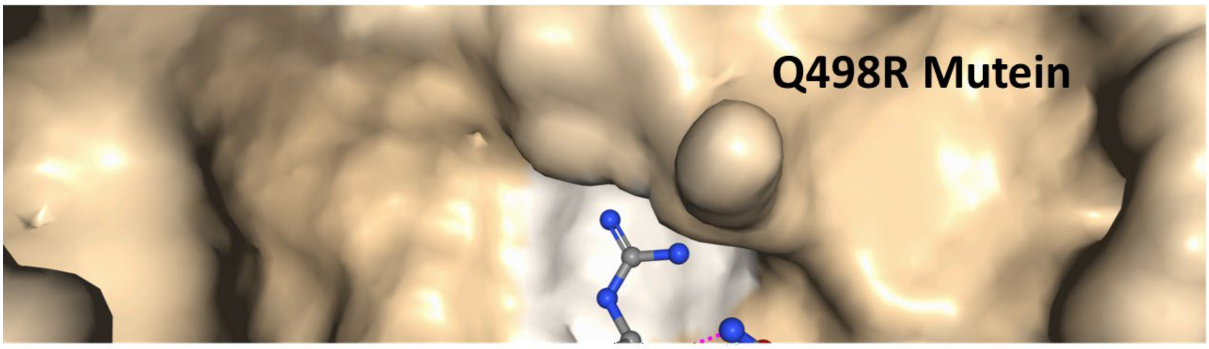
Q498R Mutein

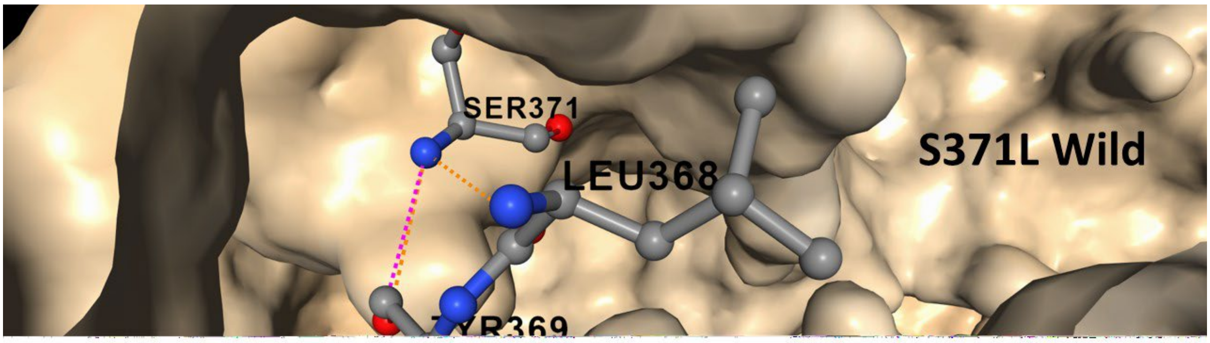
SER371
LEU368
S371L Wild

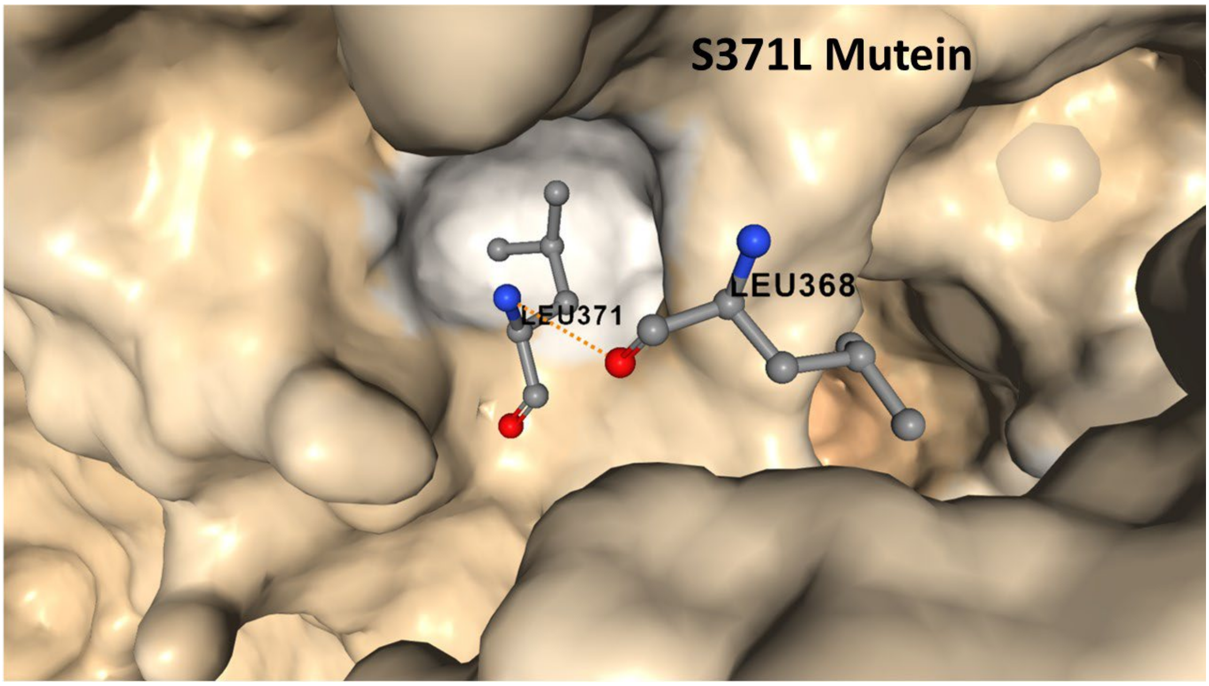
S371L Mutein
LEU368
LEU371

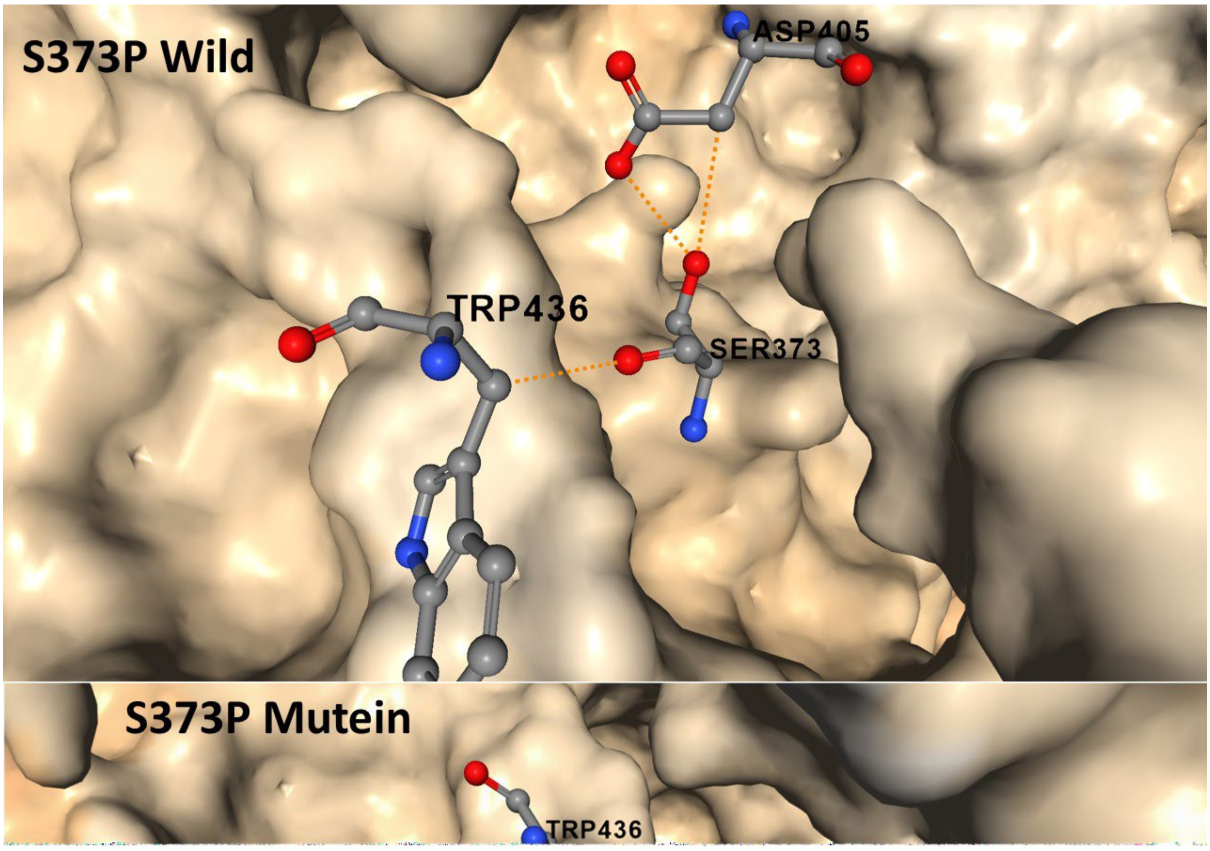
S373P Wild
ASP405
TRP436
SER373
S373P Mutein
TRP436

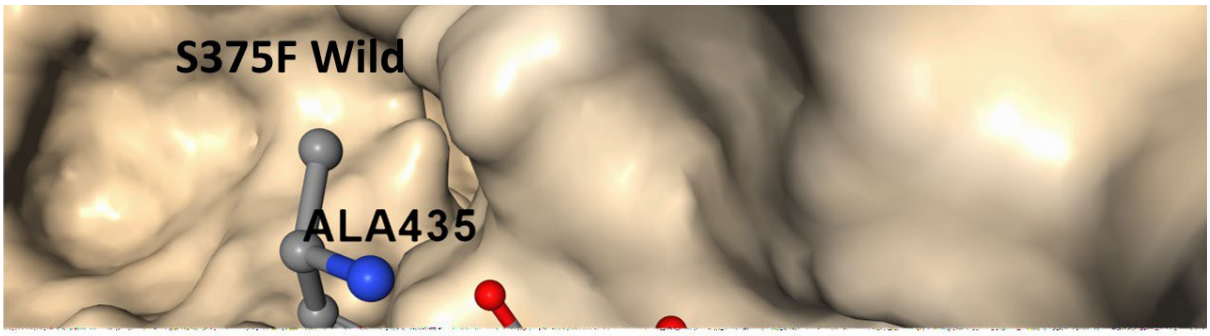
S375F Wild
ALA435

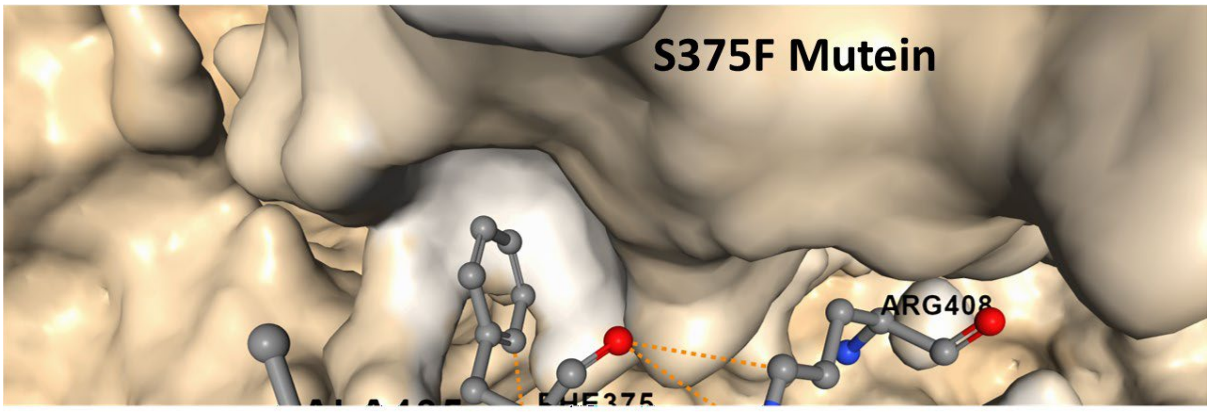
S375F Mutein
ARG408

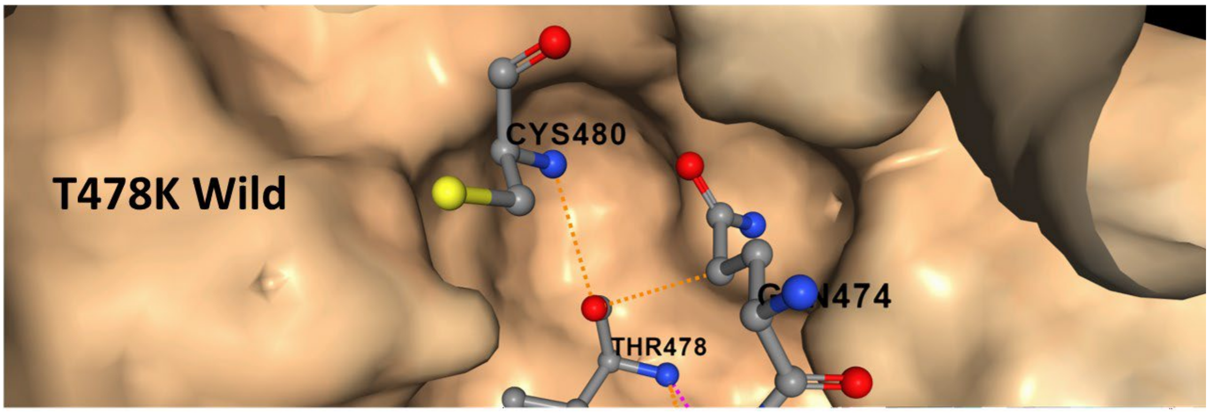
T478K Wild
CYS480
THR478

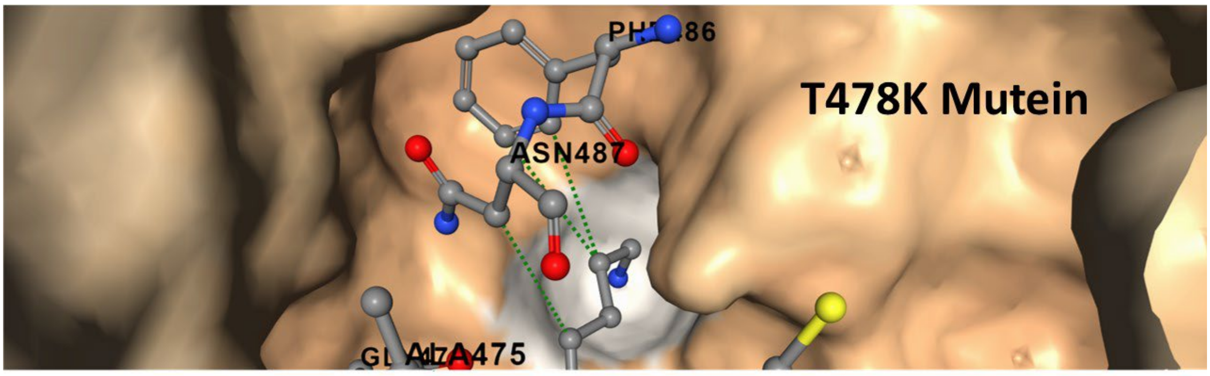
T478K Mutein
ASN487
