# Supplementary figures and images for: Variant-specific deleterious mutations in the SARS-CoV-2 genome reveal immune responses and potentials for prophylactic vaccine development
Source: Front Pharmacol. 2023 Feb 7;14:1090717. doi: 10.3389/fphar.2023.1090717 (PMC9941545; doi:10.3389/fphar.2023.1090717)

# B cell epitope prediction for Delta variant

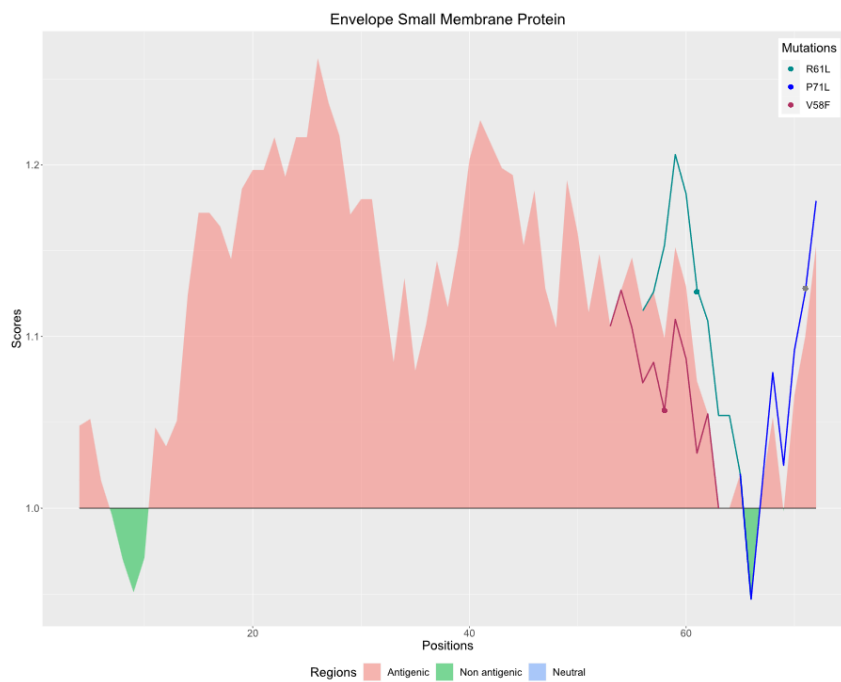

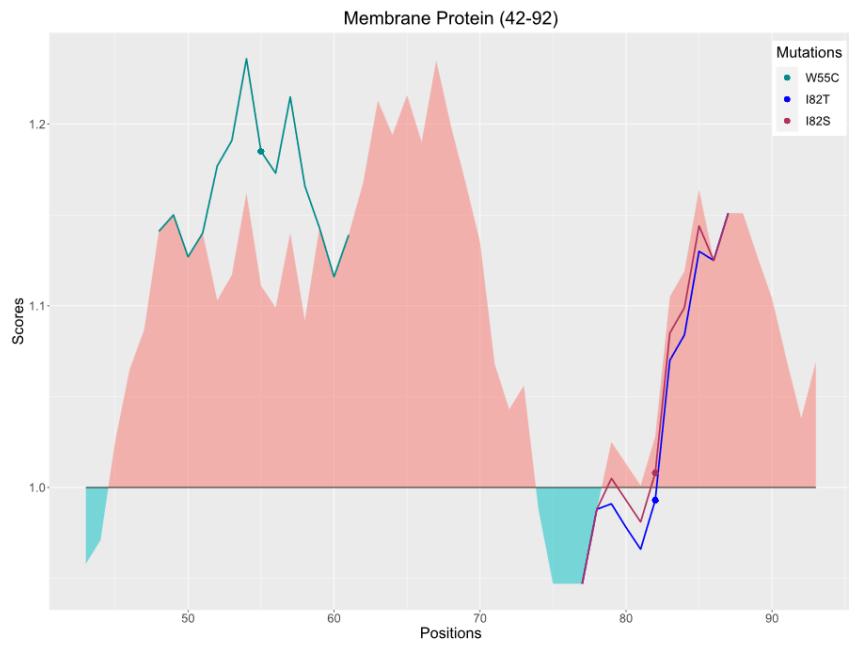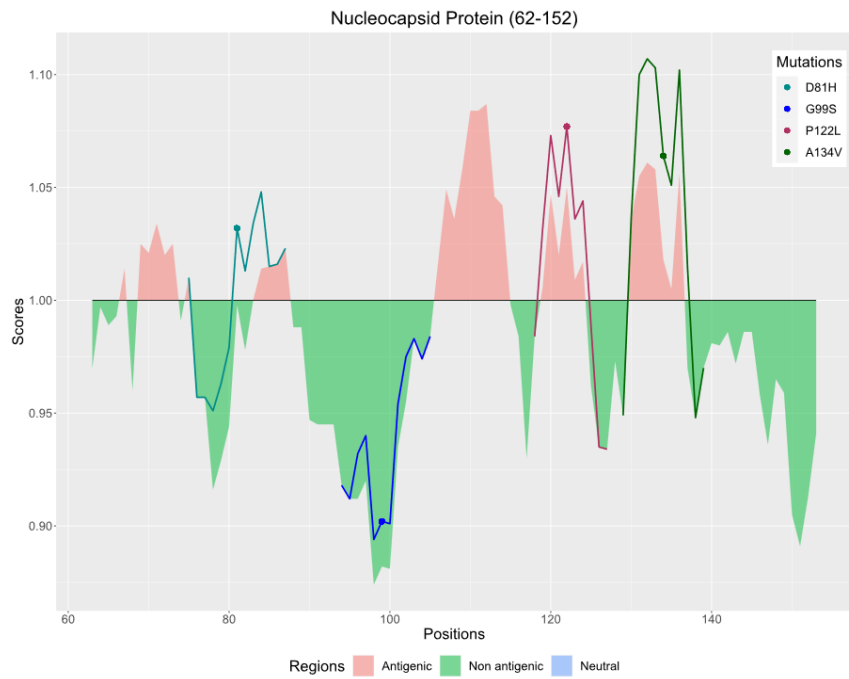

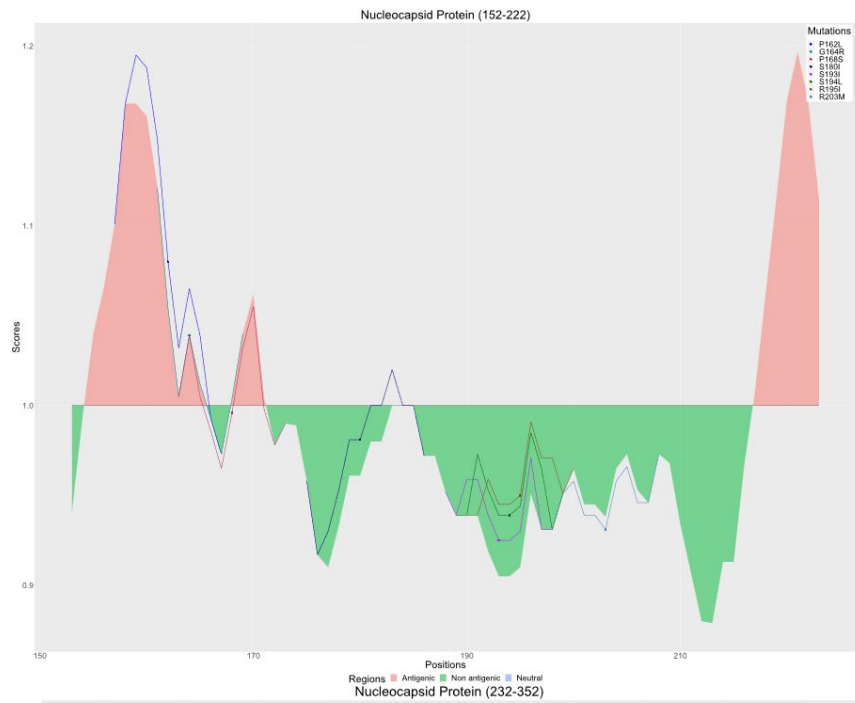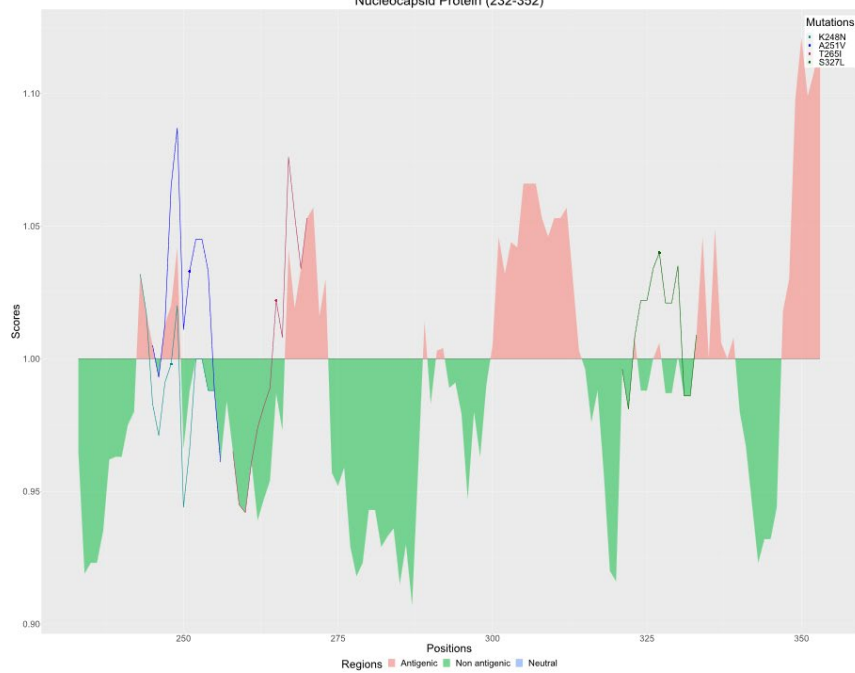

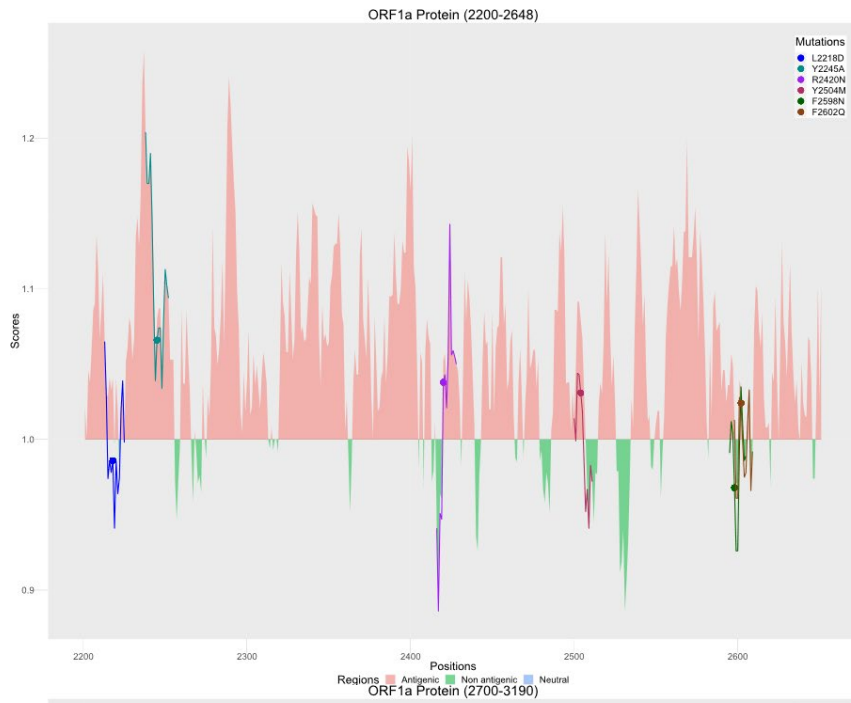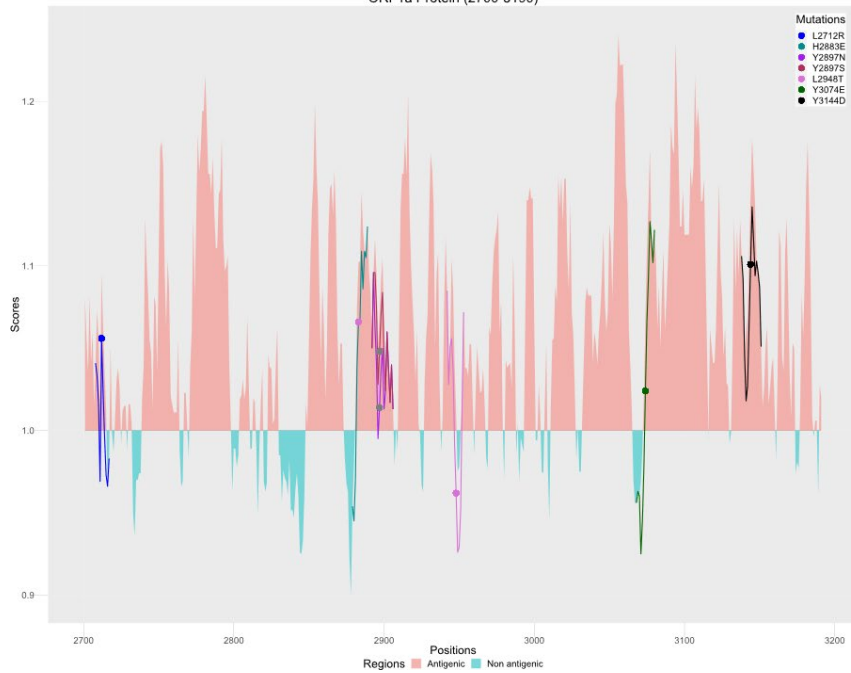

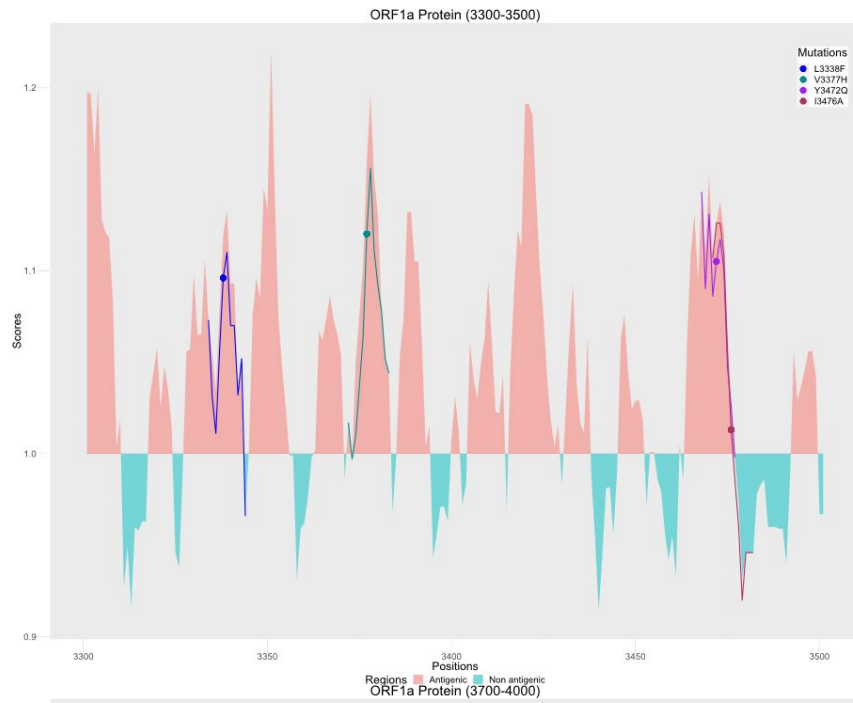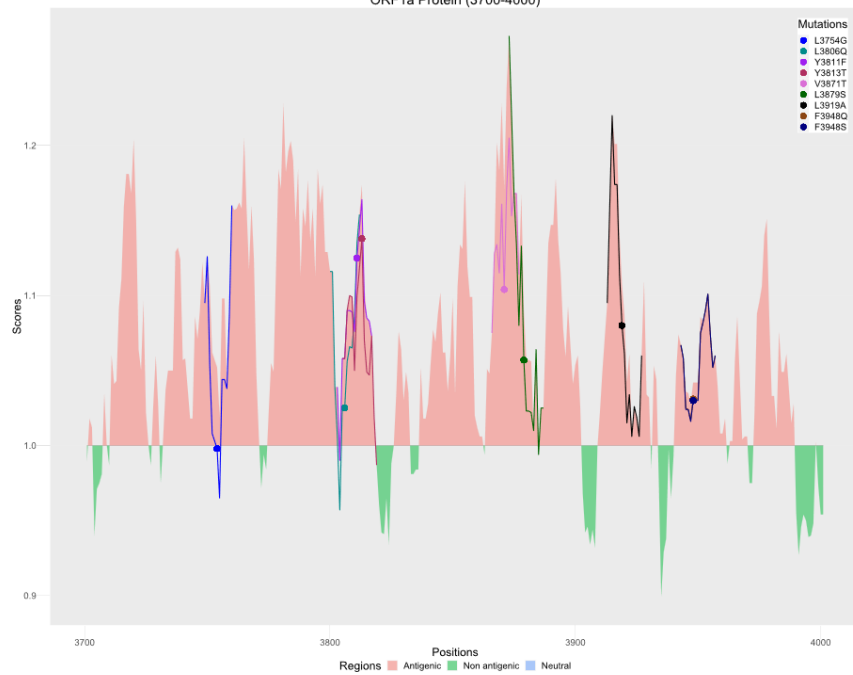

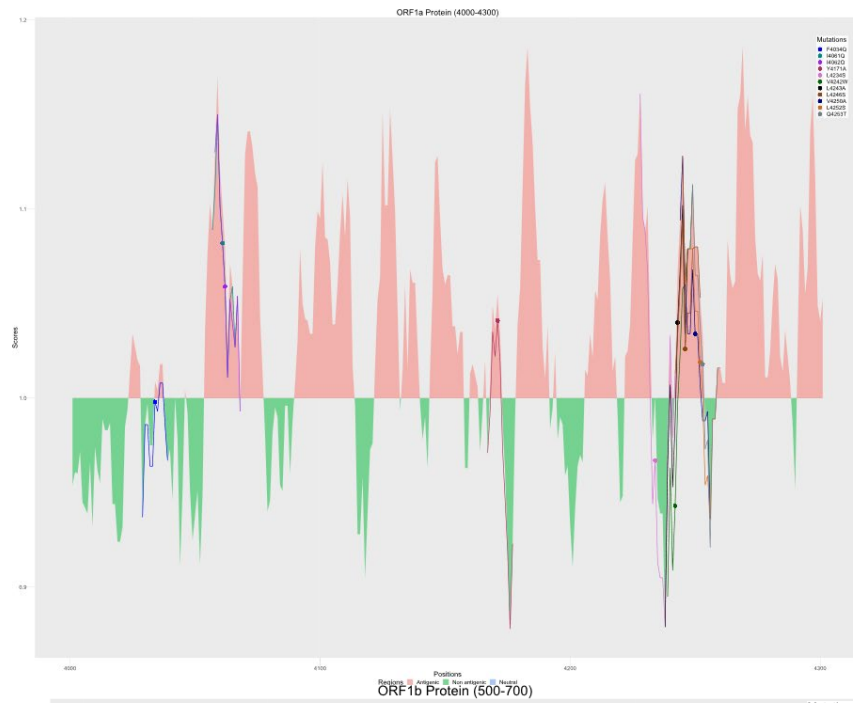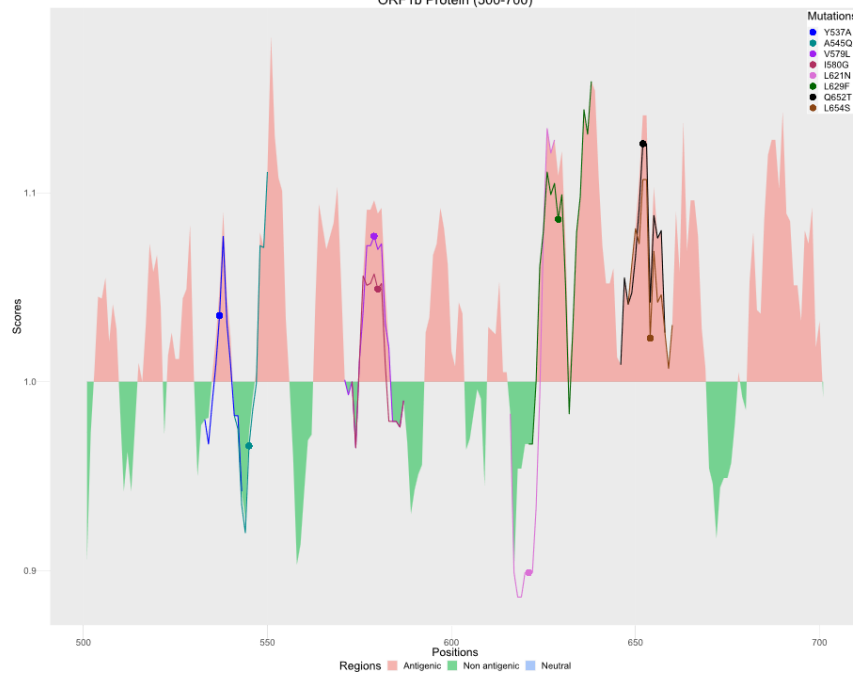

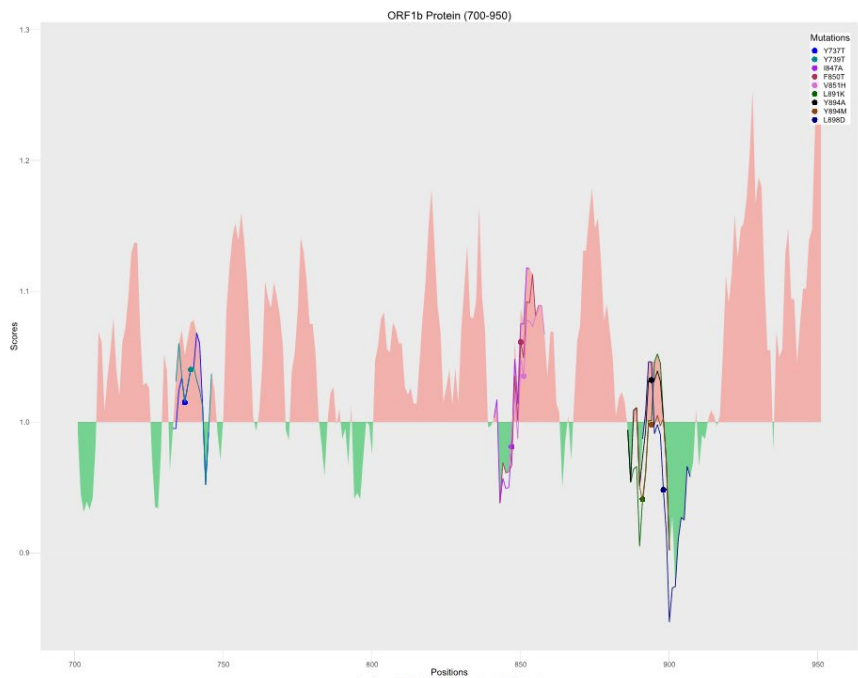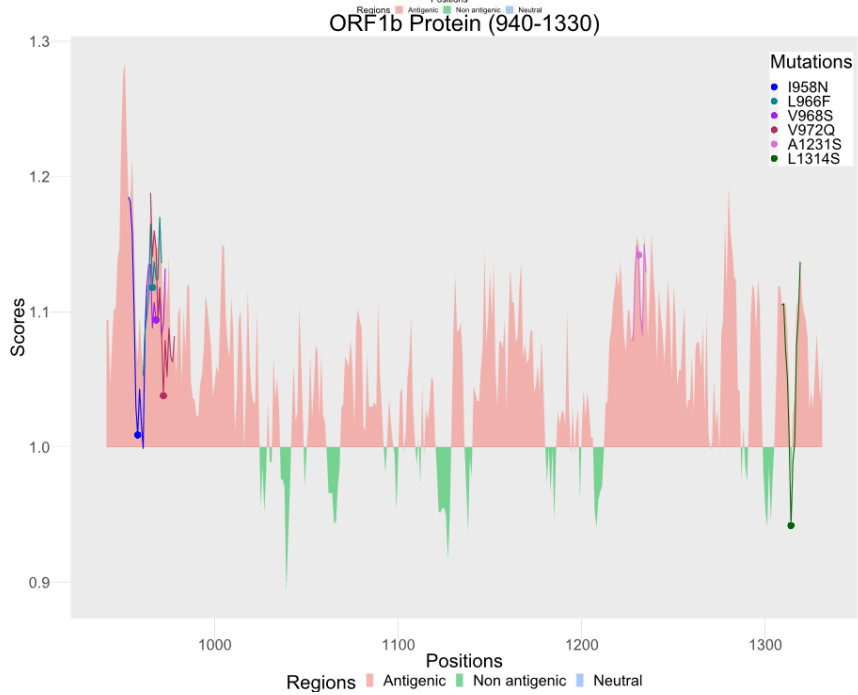

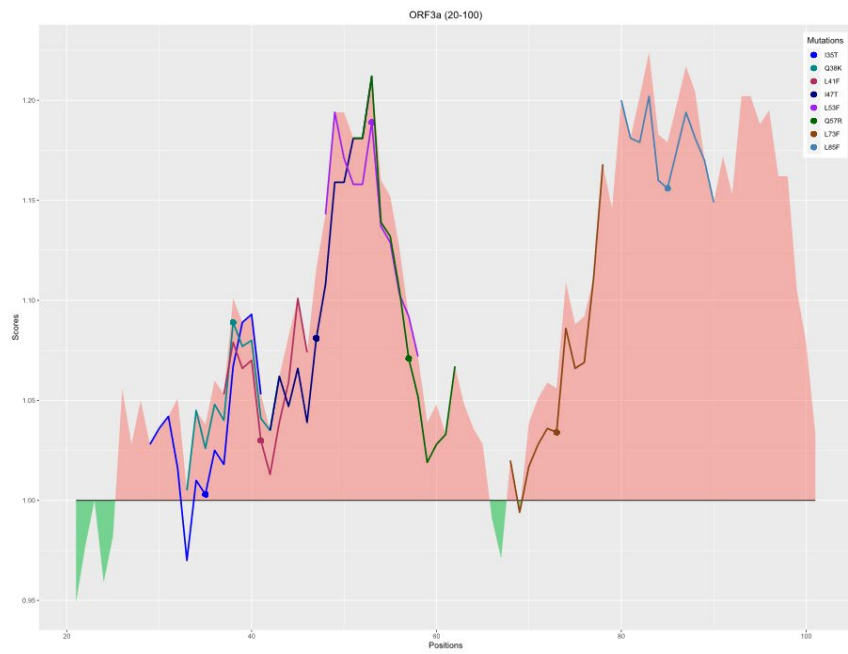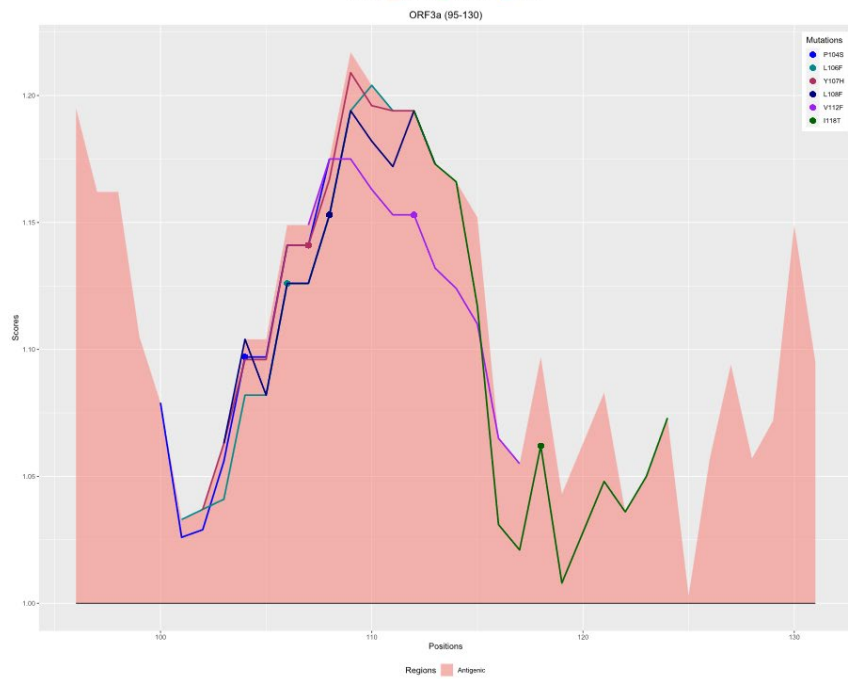

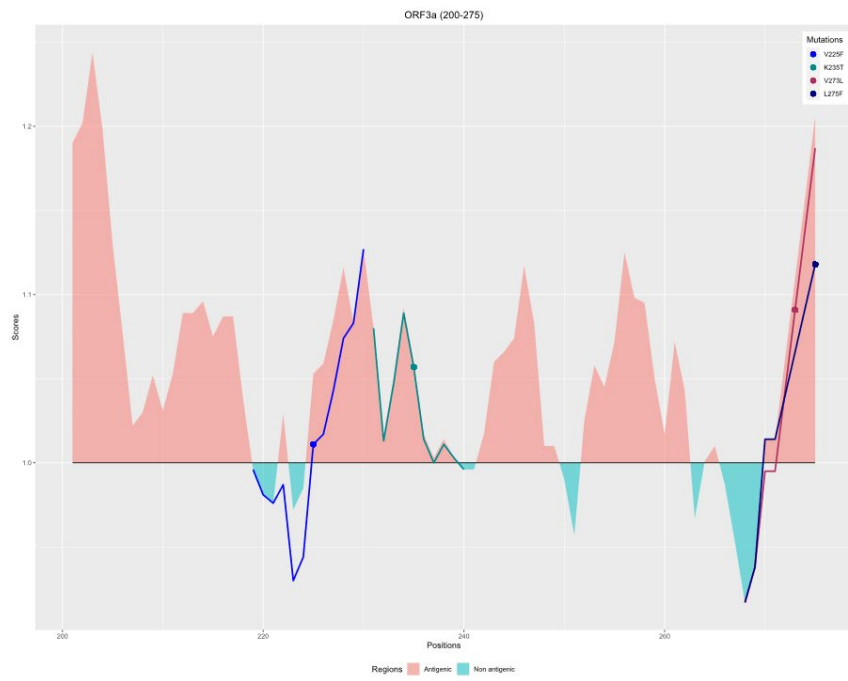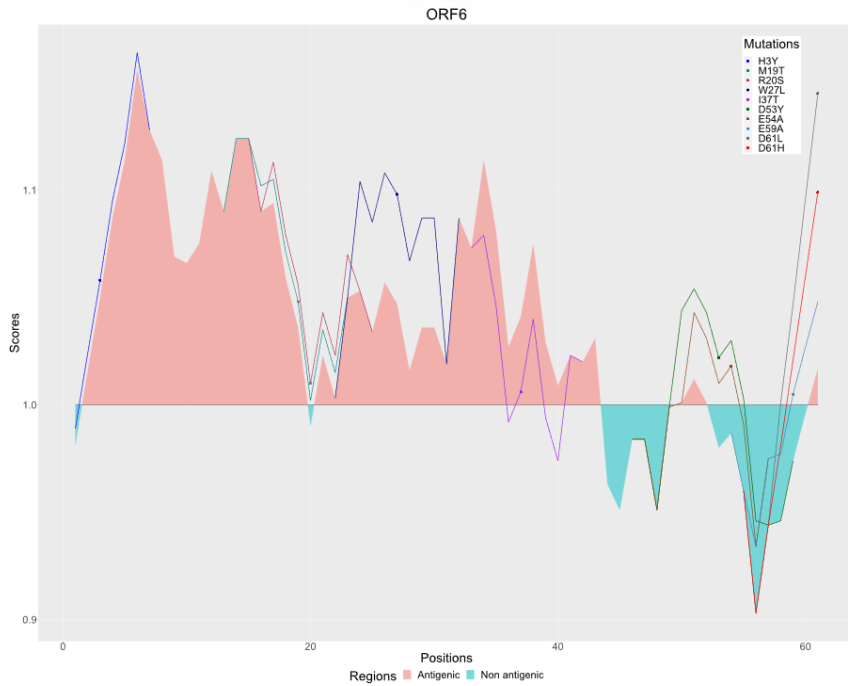

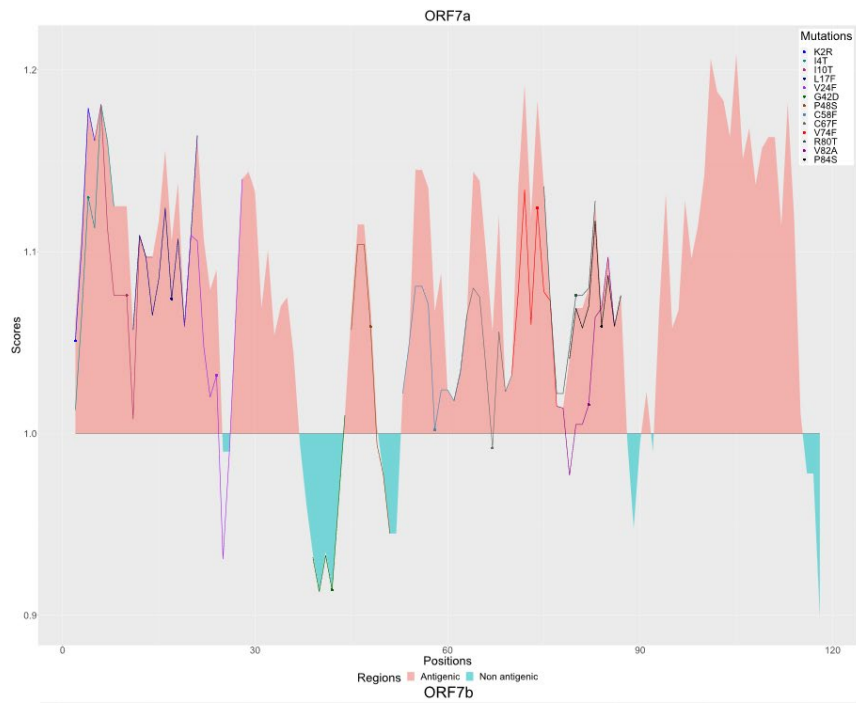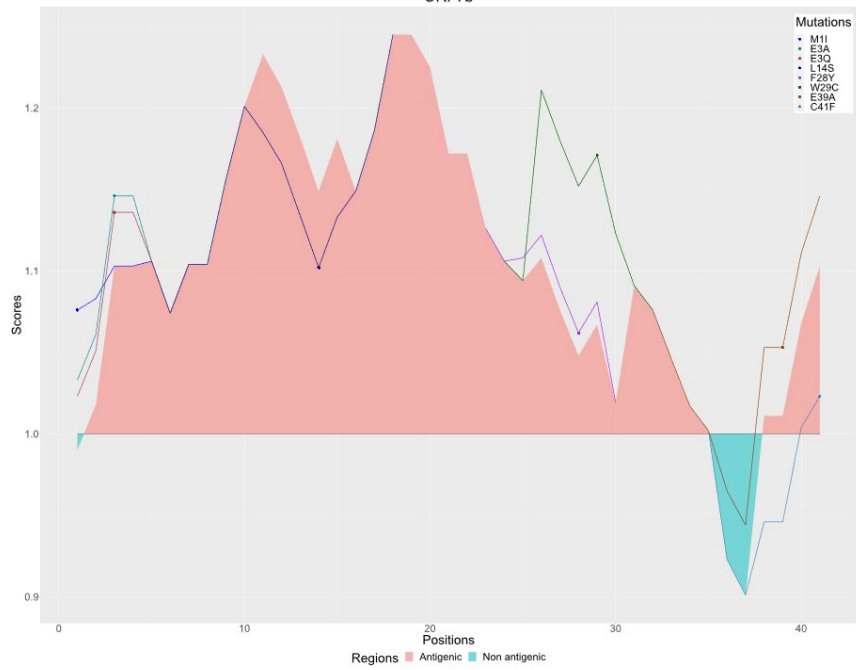

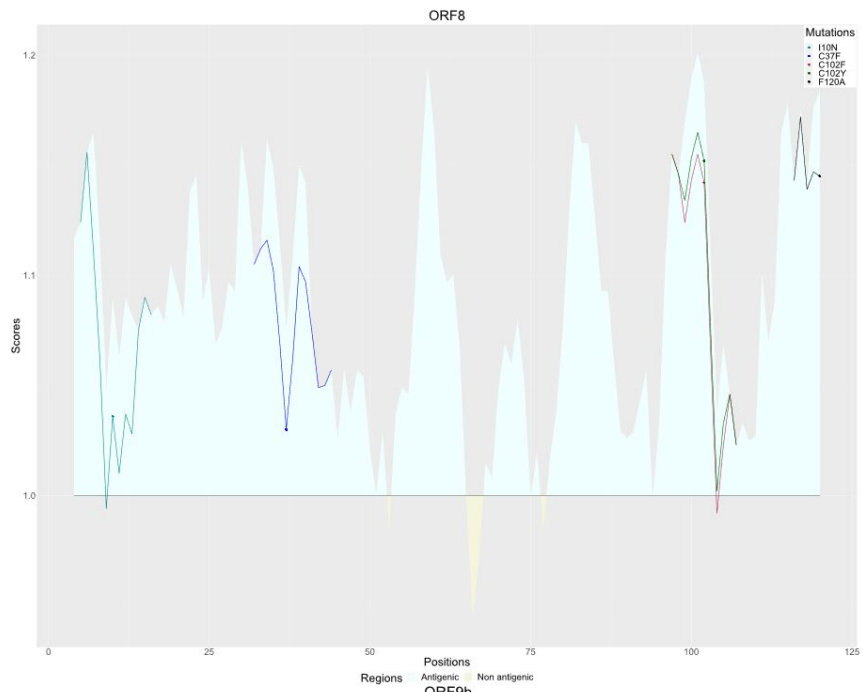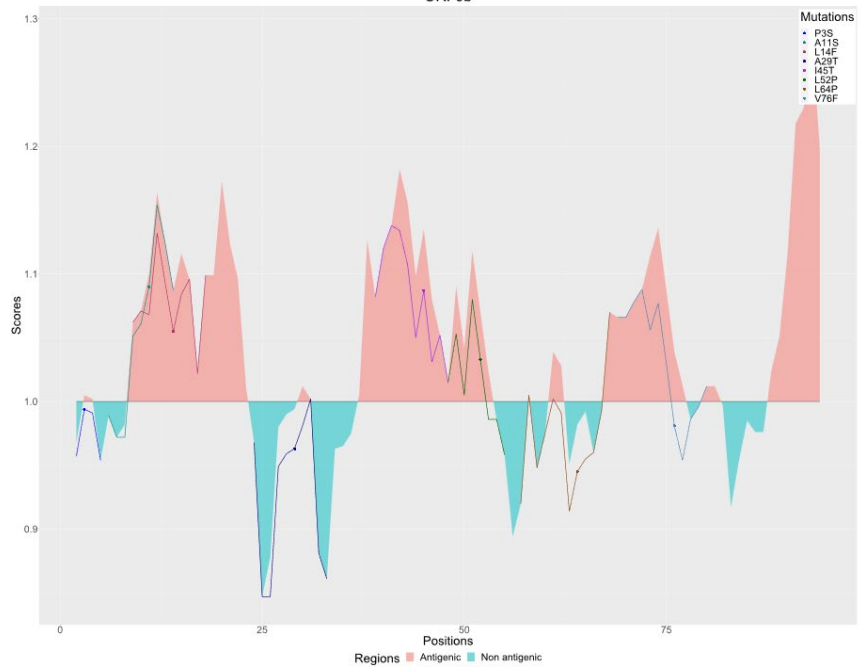

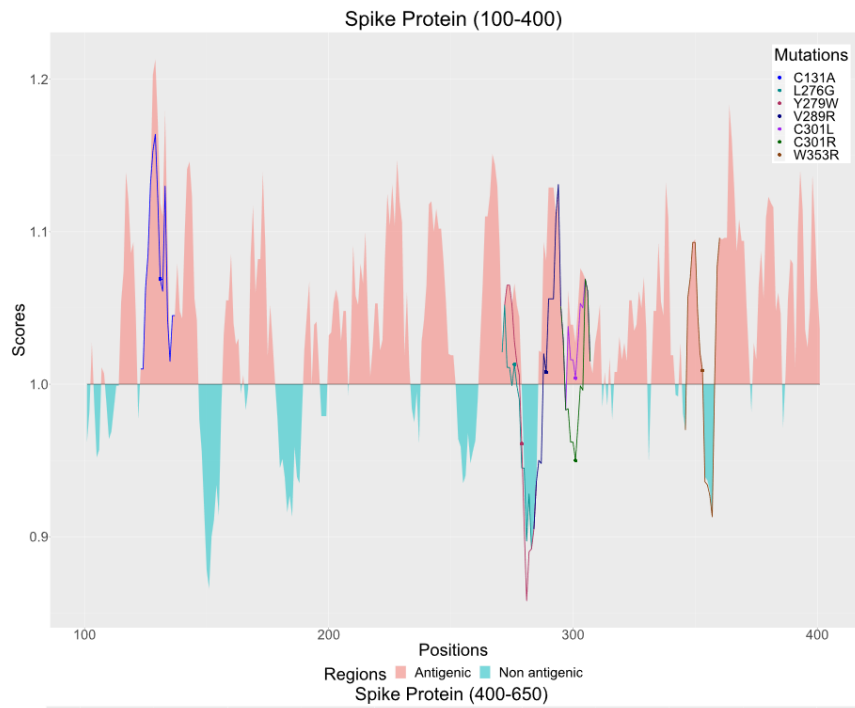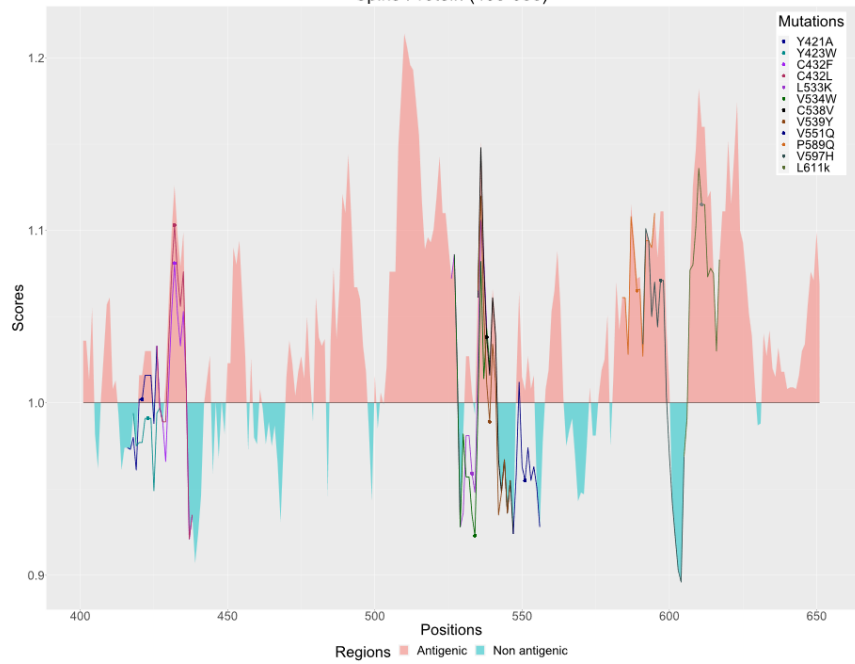

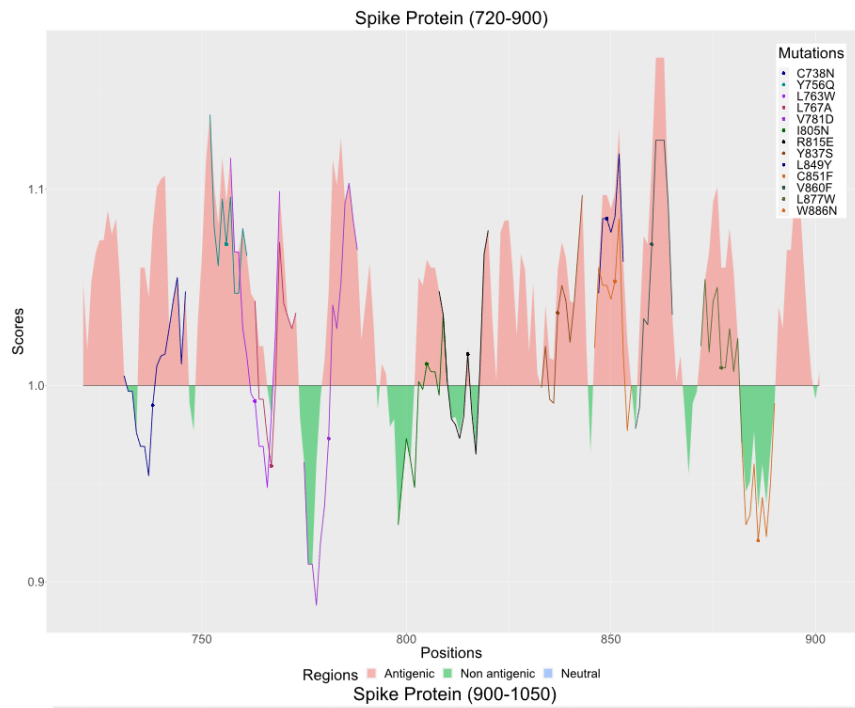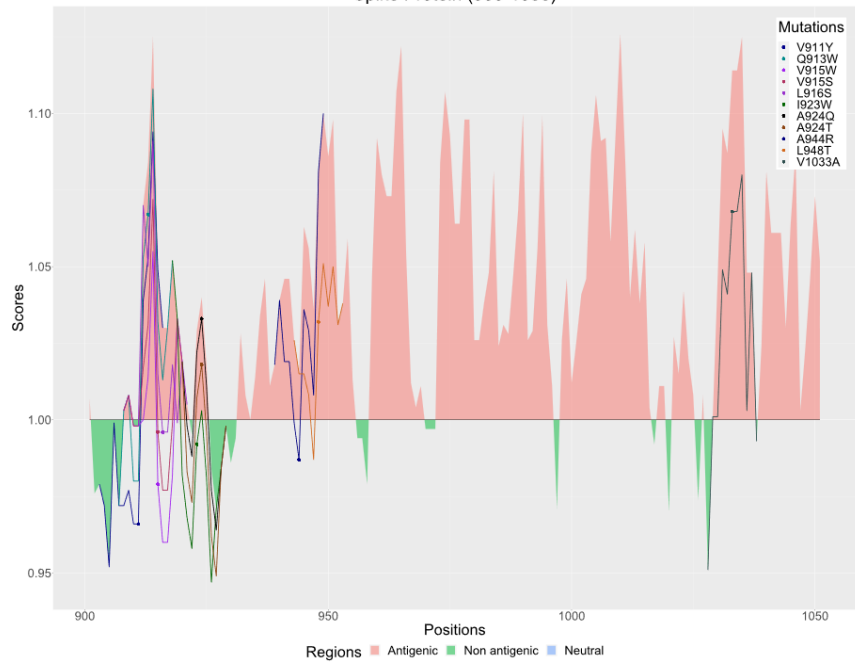

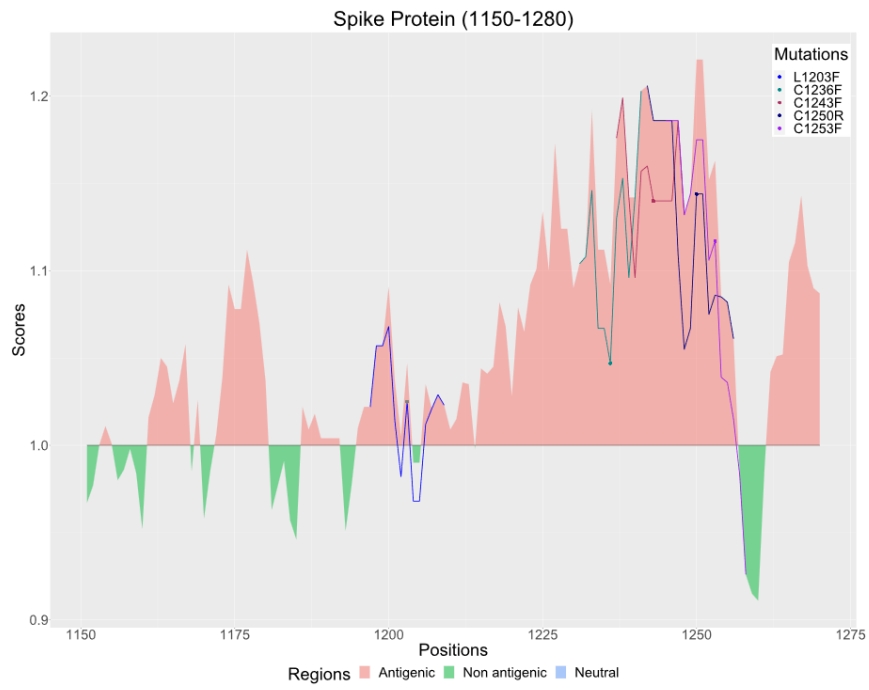

Supplement: Supplementary file 5 [file Image1.pdf]
